# Parental engagement in preventive parenting programs for child mental health: a systematic review of predictors and strategies to increase engagement

**DOI:** 10.7717/peerj.4676

**Published:** 2018-04-27

**Authors:** Samantha J. Finan, Brooke Swierzbiolek, Naomi Priest, Narelle Warren, Marie Yap

**Affiliations:** 1 School of Psychological Sciences, Monash Institute of Cognitive and Clinical Neurosciences, Monash University, Clayton, Victoria, Australia; 2 Centre for Social Research and Methods, Australian National University, Acton, Australian Capital Territory, Australia; 3 School of Social Sciences, Faculty of Arts, Monash University, Clayton, Victoria, Australia; 4 Melbourne School of Population and Global Health, University of Melbourne, Melbourne, Victoria, Australia

**Keywords:** Participation, Recruitment, Parent Engagement, Prevention, Parenting Program, Intervention

## Abstract

**Background:**

Child mental health problems are now recognised as a key public health concern. Parenting programs have been developed as one solution to reduce children’s risk of developing mental health problems. However, their potential for widespread dissemination is hindered by low parental engagement, which includes intent to enrol, enrolment, and attendance. To increase parental engagement in preventive parenting programs, we need a better understanding of the predictors of engagement, and the strategies that can be used to enhance engagement.

**Method:**

Employing a PRISMA method, we conducted a systematic review of the predictors of parent engagement and engagement enhancement strategies in preventive parenting programs. Key inclusion criteria included: (1) the intervention is directed primarily at the parent, (2) parent age >18 years, the article is (3) written in English and (4) published between 2004–2016. Stouffer’s method of combining *p*-values was used to determine whether associations between variables were reliable.

**Results:**

Twenty-three articles reported a variety of predictors of parental engagement and engagement enhancement strategies. Only one of eleven predictors (child mental health symptoms) demonstrated a reliable association with enrolment (*Stouffer’s p* < .01).

**Discussion:**

There was a lack of consistent evidence for predictors of parental engagement. Nonetheless, preliminary evidence suggests that engagement enhancement strategies modelled on theories, such as the Health Belief Model and Theory of Planned Behaviour, may increase parents’ engagement.

**Systematic review registration:**

PROSPERO CRD42014013664.

## Introduction

### Background

Mental health problems are a leading cause of disability in children and young people worldwide (World Health Organization, 2016a). Mental health problems can be defined as a dysregulation of mood, thought and/or behaviour, and categorized more broadly into internalizing or externalizing problems for children (American Psychiatric Association, 2013). In the current review, ‘mental health problems’ is used to encapsulate both internalizing and externalizing problems (American Psychiatric Association, 2013). Mental health problems are typically followed by subsequent adverse outcomes for children including psychological distress, functional impairment, exposure to stigma and increased risk of premature death (Patel et al., 2007).

The World Health Organization (WHO) has called for greater attention to be given to the prevention and promotion of mental health at all levels of society (WHO, 2016b). It is important here to differentiate between mental health promotion and prevention, as these terms are often confused or conflated. Health promotion is the development, progress or establishment of practices that increase overall health and wellbeing (Czeresnia, 1999), and is not addressed in the current review. Instead, the focus is on prevention, defined as “interventions directed to averting the emergence of specific diseases, reducing their incidence and prevalence in populations” (Czeresnia, 1999, p. 705). Despite varying definitions for different stages of prevention, here we follow Haggerty & Mrazek’s (1994) widely adopted stages of *universal, selective* and *indicated*. The goal of *universal* prevention is to target the public and deliver an intervention that can minimize potential risk and increase protective factors for mental health. *Selective* prevention is designed to deliver interventions to individuals whose risk of developing a mental health problem is higher than others in the population, while *indicated* prevention specifically targets persons at high risk.

### Parenting programs to prevent child mental health problems

Recent systematic reviews demonstrate that parenting behaviours (i.e., less warmth, and more inter-parental conflict) are associated with children’s and adolescents’ risk of mental health problems (Rothbaum & Weisz, 1994; Yap & Jorm, 2015; Yap et al., 2014). Hence, parenting programs that aim to modify parenting behaviours have the potential to prevent mental health disorders in children and adolescents. These programs, whether face-to-face or online, have shown promise in preventing both internalising disorders (Yap et al., 2016) and behaviour problems, as well as increasing other child competencies (Sandler et al., 2015; Sandler et al., 2011). Parenting programs can be defined as any intervention delivered to parents with the main objective of increasing parental knowledge, skills and confidence, whilst reducing the prevalence of mental health, emotional and behavioural problems in children and adolescents (Sanders et al., 2008). These programs assume that changing parenting behaviours will in turn alter a child’s risk of developing mental health problems. This assumption stems from theoretical underpinnings suggested by Sandler and colleagues (2011), that a parenting program improves parenting skills and parental self-efficacy, causing a reduction in barriers to effective parenting, and in turn facilitating long-term benefits for the child. Despite the potential benefits of preventive parenting programs, many studies examining the effectiveness of such programs have reported difficulties in engaging parents (Gross, Julion & Fogg, 2001; Ingoldsby, 2010; Morawska & Sanders, 2006; Orrell-Valente et al., 1999; Panter-Brick et al., 2014). Poor parental engagement, including parental uptake and ongoing engagement (attendance), could lead to both the effectiveness of these programs being under-reported and parents not adequately developing the key skills required to prevent mental health problems (Morawska & Sanders, 2006).

Parental engagement in preventive parenting programs has been defined inconsistently across studies (Gross, Julion & Fogg, 2001; Orrell-Valente et al., 1999). However, these definitions can generally be broken down into three discrete components. The first component, *initial engagement*, includes two phases: (1) parental *intent to enrol*, measured either through initial expression of interest rates, occurring prior to or separate from signing consent forms, or through a direct question (e.g., ‘Do you intend to enrol?’); and (2) actual *enrolment*, as described by the study, as the number of parents who enrolled in the program (e.g., number of parents who signed a consent form) (Dumas, Nissley-Tsiopinis & Moreland, 2007; McCurdy & Daro, 2001; Spoth et al., 1996). The second component, *ongoing engagement*, is measured by the proportion of parents attending at least one session or completing at least one module of a self-administered or online intervention, the total number of sessions attended by parents, or the number of parents who completed the program (Dumas, Nissley-Tsiopinis & Moreland, 2007; McCurdy & Daro, 2001; Spoth et al., 1996). The third component of engagement, *quality of engagement*, measures both what parents invest in and receive from the program (e.g., taking part in group discussions or completing homework tasks; Chacko et al., 2016; Orrell-Valente et al., 1999). This component (1) is determined by the type of activities parents are asked to take part in during sessions, specific to each program; and (2) is suggested to form part of the key mechanism for positive parenting change (Kazantzis, Deane & Ronan, 2000; Piotrowska et al., 2017), thus is more closely related to program outcomes than the other two components.

### Engaging parents in prevention

In two recent reviews, Ingoldsby (2010) and Chacko et al. (2016) attempted to collate research on parental engagement. Ingoldsby (2010) reviewed *ongoing engagement* and retention of families attending both intervention and indicated prevention programs designed to improve child mental health (child age range not specified). The main findings from Ingoldsby’s (2010) review included: (1) brief strategies implemented at the beginning of the program addressing families’ practical and psychological barriers effectively increased engagement in early sessions; and (2) strategies that were ongoing throughout the intervention, and focused on motivational interviewing, family systems and family stress, demonstrated longer-term increased engagement. More recently, Chacko and colleagues (2016) reviewed and discussed predictors of parental engagement, including the domains of attendance and attrition (*ongoing engagement*) and treatment adherence (*quality of engagement*). Chacko and colleagues (2016) concluded that at least 51% of parents drop out at some stage of the intervention, with this high level of attrition found to be somewhat influenced by lower socio-economic status (SES). Both reviews adopted inclusion and exclusion criteria that may have resulted in the exclusion of studies focusing on universal and selective prevention programs (i.e., Heinrichs et al., 2005; Spoth & Redmond, 2000), as well as those using open access recruitment methods.

### Engaging parents across children’s lifespan

During the developmental transition into adolescence, the corresponding changes in the parent–child relationship include increasing autonomy and time spent apart from the parent, and an increased importance of peer relations (Collins, 1990). In this context, parents may perceive that their role in their child’s mental health and well-being is no longer as important as when their child was younger. This could account for the low rates of engagement in preventive programs for parents of adolescents (Burke, Brennan & Roney, 2010). However, there is a substantial body of evidence to suggest that even when a child moves into adolescence, parents still play an important role in their child’s risk for both internalising (Yap et al., 2014) and externalising disorders (Sandler et al., 2011). Chacko and colleagues’ (2016) review only assessed parental engagement in programs for parents of children aged 2–12 years, finding no effect of child age. Hence, it remains unclear if parental engagement in programs differs depending on the age of the child, when considering the whole developmental period from birth through to late adolescence (0–18 years).

### Engagement enhancement strategies

In addition to studies that have examined predictors of parents engaging in a preventive parenting program, there is emerging research on the effectiveness of engagement enhancement strategies for parental engagement. Ingoldsby (2010) found that additional strategies implemented at the time of enrolment, including brief intensive engagement interventions that are both practical (e.g., providing transportation) and psychological (e.g., addressing beliefs about the treatment process) in nature, could increase parental attendance during early stages of the intervention. In another review of strategies to recruit any type of participant into a RCT, Caldwell et al. (2010) found that any strategy that increased a participant’s awareness of the health problem being studied increased recruitment, a finding that is in line with the Health Belief Model (Rosenstock, 1974). The Health Belief Model was proposed to explain and predict health-related behaviours, such as attending health care appointments. This model focuses on the attitudes and beliefs of individuals; for instance, if a parent has increased awareness of their child’s susceptibility to developing a mental health problem, they may be more likely to engage in a preventive parenting program. In addition to the Health Belief Model, the Theory of Planned Behaviour and Reasoned Action (Ajzen & Driver, 1991) may also be used to inform engagement enhancement strategies (McCurdy & Daro, 2001). The Theory of Planned Behaviour and Reasoned Action links an individual’s beliefs and attitudes about subjective norms (e.g., the perceived social pressure to perform or not perform a behaviour; Ajzen & Driver, 1991) and perceived behavioural control (e.g., a parent’s perceived ease or difficulty of performing a certain behaviour; Ajzen & Driver, 1991). For example, McCurdy & Daro (2001) posit that subjective norms, as determined by communications with others, can influence a parent’s decision to engage in a parenting program. Therefore, it appears there are additional strategies researchers can utilise during the initial engagement stage of a study to increase parental engagement across subsequent stages. Engagement enhancement strategies are defined in the current review as any methodology that looks to use evidence- or theoretically-based strategies to increase parental engagement.

### The current systematic review

The current review will extend on previous findings by reviewing both potential predictors of engagement as well as engagement enhancement strategies used. Specifically, the current review aims to delineate factors/strategies that can be applied across different types of parenting programs. Hence it focuses on the *initial engagement* and *ongoing engagement* components, but not *quality of parental engagement*, because the latter is related to program-specific components. Such a synthesis can inform researchers regarding the predictors of *initial* and *ongoing* parental engagement, and suggest some possible theoretical models that could be used in the development of engagement strategies to increase the uptake of evidence-based preventive parenting programs. To identify factors predicting parental engagement in programs where parents are the main target of intervention, the current review follows Yap and colleagues’ (2016) definition of a ‘preventive parenting program’: a program aimed at preventing child mental health problems through education and subsequent skill development of parent and primary caregivers, that specifically involves parents in more than 50% of the program. Additionally, the current review will include all programs specifically focused on the prevention of child mental health problems, across both childhood and adolescence (0–18 years), to explain the association between child age and parent engagement. This review aims to shed light on whether there is a more optimal time to promote parenting programs, and inform future parenting program design and implementation, with the ultimate goal of maximising parental uptake of preventive programs.

Specifically, this review aims to: (1) investigate the predictors of parental engagement in preventive parenting programs, across the *initial engagement* (intent to enrol and enrolment) and *ongoing engagement* (attendance) components. Of particular interest, we aim to examine whether parental engagement differs depending on the age of the child at the time of parent participation; and (2) explore whether any strategies used by researchers to increase parental engagement have been successful.

## Materials and Methods

### Search strategy

This review was conducted following the Cochrane Collaboration guidelines (Higgins & Green, 2008). The following electronic databases were searched: Cochrane Library, Informit online, Ovid MEDLINE, ProQuest, PsycINFO, PubMed and Scopus. The search was limited to studies written in English and articles published between 2004–2014. This publication date range was chosen to increase the likelihood that findings from this review will be more recent and relevant to current and future parenting programs. The initial search was conducted by the first author (SF) on the 12th of January 2015. To ensure the latest data was included in the review, an update search was conducted (also by SF) on the 21st of July 2016 to include articles published between January 2015–July 2016. Search terms included multiple terms for engagement, parents, programs, prevention, child and mental health (for a full list, refer to Supplemental Information 2).

Unpublished reports, dissertations and grey literature were sourced through Google Scholar and dissertation databases. Manual searching of reference lists of included studies was conducted to locate further relevant articles and dissertations of interest.

### Inclusion and exclusion criteria

Studies were included if they were controlled trials (randomised and non-randomised), cross-sectional, case-control, and longitudinal studies. Excluded studies included; therapy/treatment interventions (*note: all prevention interventions were eligible for inclusion)*, reviews or meta-analyses, qualitative studies, discussion papers, and papers published in languages other than English.

#### Population

Participants included parents who were defined as parents or primary caregivers (aged 18 or older) of children aged 0–18 years. This wide child age range was used to maximise variance and the number of eligible studies, to explore whether child age is associated with parental engagement. Parents were taking part in an intervention those designed to prevent the development of mental health problems in children, where parents took part in at least 50% of the intervention. Interventions could be either group or individual programs delivered face-to-face, via phone, mail or internet. Therefore, studies were excluded if they; compared diagnostic groups but did not include a normal (non-clinical) control group, and/or were evaluating therapy or treatment for children with existing depression or anxiety disorders

#### Intervention

The focus of this review includes the testing of different recruitment methodologies and the discovery of potential predictors of parental engagement. Therefore, the intervention of focus includes recruitment methodologies. Studies were excluded if they lacked adequate detail in describing recruitment methods and/or used non-specific measures (e.g., measure of general psychopathology).

#### Comparison

For RCT’s comparisons could include alternative recruitment methodologies or recruitment as usual, however, studies did not need to have a comparison group to be included in the *Stouffer’s p* analysis.

#### Outcomes

Studies were required to contain analyses of the predictors of parent engagement, and/or an evaluation of the effects of an engagement strategy on parents’ subsequent engagement in the parenting program (see Supplemental Information 2 for further detail about inclusion/exclusion criteria).

### Study selection and data extraction

**Figure 1:**
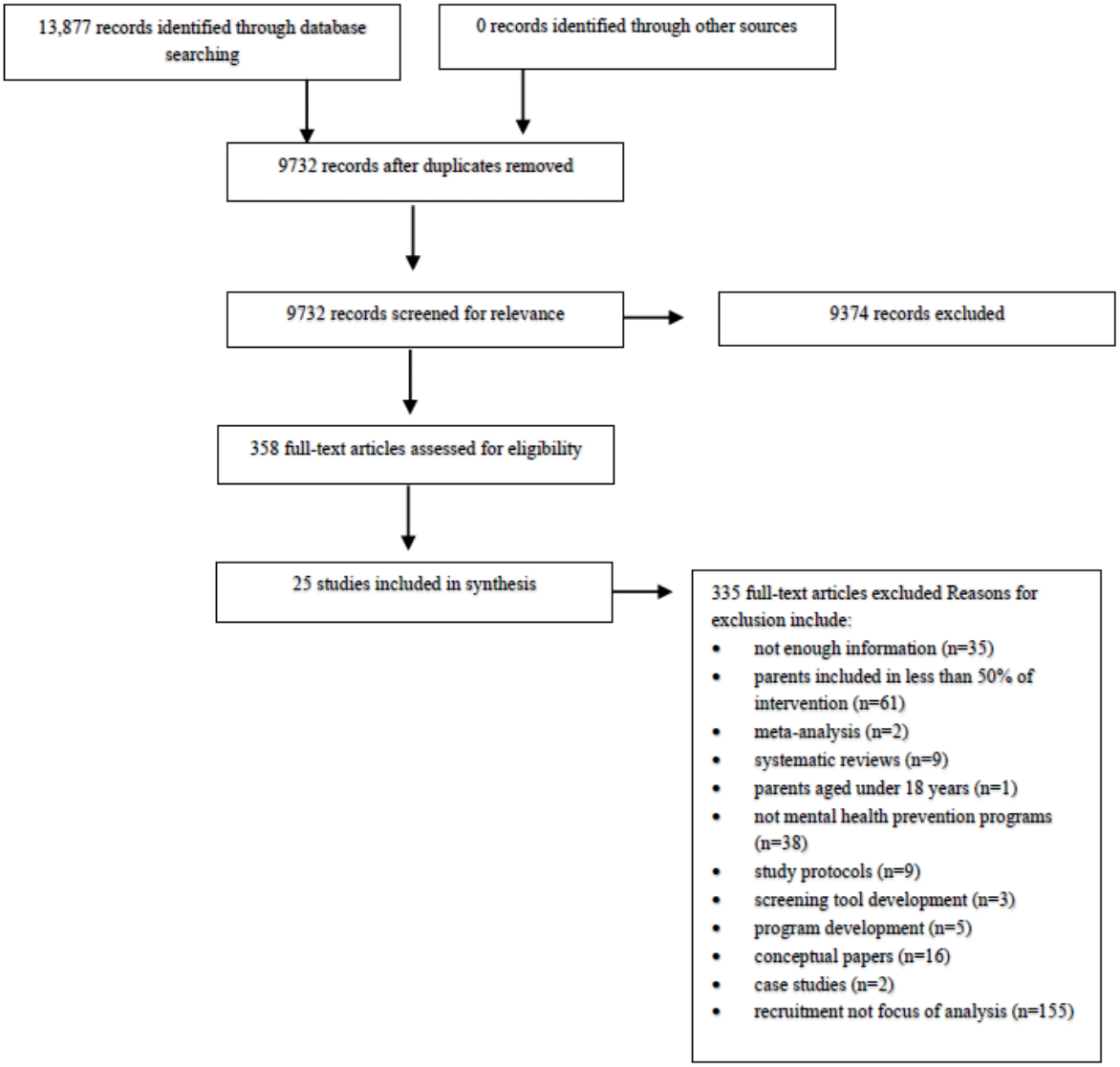
PRISMA diagram.

Titles and abstracts of identified studies were reviewed to determine if they met inclusion criteria. Full texts of articles that met inclusion criteria were assessed by the first author (SF). Thirty-five percent of these titles and abstracts were independently assessed by a second author (BS) to confirm inter-rater reliability of the inclusion criteria. Inter-rater reliability of inclusion criteria was 99.2%, with one additional article being included in the review. All reasons for exclusion are documented in the PRISMA diagram (Fig. 1). Disagreements were resolved through discussion and review of the extraction sheet by SF and BS, with involvement of other authors (NP and MY) when necessary.

### Coding of predictors

Engagement factors were identified as factors that could influence a parent’s engagement in preventive parenting programs. Categories were specified when two or more of the included studies examined the same engagement variable. The categories identified included: *parent age, gender of parent, parent education status, parent employment status, parent race/ethnicity, parental mental health status, child age, child gender, child mental health symptoms, family structure* and *one- or two- parent households*. Definitions for these eleven categories were based on consensus in the relevant literature (see Table 1 for definitions and examples of measures used). In addition, several other factors could not be coded into categories, including parenting behaviour measures and individual and neighbourhood socioeconomic status. Of the six studies which assessed parenting behaviours as a predictor, the types of parenting behaviours were too diverse to combine into a single meaningful category (Yap et al., 2014; Yap & Jorm, 2015). The eight different parenting behaviour categories included; discipline (*n* = 3), parent self-efficacy (*n* = 2), parent warmth (*n* = 1), positive parenting style (*n* = 2), negative attribution/conflict (*n* = 2), knowledge of school performance (*n* = 1), restrictive attitude to alcohol (*n* = 1) and parenting problems (*n* = 1). Those parenting behaviour categories with *n* > 1 were incomparable due to the use of different indicators, and/or they were used to predict different stages of engagement. Additionally, as recommended by Braveman and colleagues (2005), measurement of specific socioeconomic factors were assessed separately rather than combined as an overall socioeconomic position (SEP). This resulted in the following SEP-related categories: *parent education status, parent employment status, family structure* and *one- or two- parent households*.

**Table 1:** Engagement factors, definitions and example measures and items.

Theme	Definition	Example items	Example measures
Parent age	Parent’s stated age in years	• Please state your age	• Study specific
		• Categories i.e., ‘18–29′, ‘30–39′ years	
Gender of parent	Parent’s stated gender/sex	• Please select one ‘male’, ‘female’, ‘prefer not to answer’	• Study specific
Parent education status	Parent’s reported highest completed education	• Categories ‘8th grade’ to ‘professional degree’	• Study specific
		• Please state highest achieved education	
Parent employment status	Involvement in paid employment	• No. of hours in paid employment	• Study specific
		• Categories, i.e., ‘unemployed’, ‘part-time’, ‘full-time’	
Parent race/Ethnicity	Parent’s statement of belonging to a social group or identifiable culture	• Categories with different ethnic group listed i.e., ‘Australian’, ‘African American’	• Study specific
		• Immigration status	
Parent mental health status	Parent’s reported psychological and emotional well-being as operationalised by standardised measures	• ‘I found it difficult to relax’	• Depression, Anxiety, Stress Scales
		• ‘Feeling blue’ or ‘feeling no interest in things’	• Brief Symptom Inventory
Child age	Age of target child in either years or months	• ‘How old is your child?’	• Study Specific (Parent-report)
		• List of eligible ages	
Child gender	The gender/sex that the child is identified as	• Please select one ‘male’, ‘female’, ‘other’	• Study Specific (Parent-report)
Child mental health symptoms	Child’s reported severity of symptoms of psychological and emotional distress and/or a dysregulation of mood, thought and/or behaviour, with these being categorized more broadly into internalizing or externalizing problems for children (American Psychiatric Association, 2013).	• ‘Argues a lot’ and ‘too fearful or anxious’ on scale of 0 = not true, 1 = sometimes or somewhat true, 2 = exactly/often true	• Eyberg Child Behavior Inventory
			• Child Behaviour Checklist
			• Social Behaviour Questionnaire
Family structure	The ratio of children to adults living in the family home	• ‘How many adults live in your home?’ and ‘how many children live in your home?’	• Study Specific
One- or two- parent households	The number of parents living in the household	• Categories, i.e., ‘single parent’, ‘married’, ‘divorced’, ‘living with a partner’	• Study Specific

### Data analysis and Stouffer’s *p*

A meta-analysis to assess effect sizes was not possible due to differences in interventions, settings, predictor variables, and analytic methods. To compensate for this limitation, the Stouffer’s method (Stouffer et al., 1949) of combining *p*-values was used to synthesize the findings of many of the included studies, since it can be applied in cases where studies analyse data in a variety of ways. Stouffer’s *z* was calculated by dividing the sum of the *z*(*p*_*i*_) values by the square root of *k* (where *k* refers to the number of associations). Stouffer’s *z* s were calculated to determine the overall *p*-value of the associations reviewed for each combination of predictor category and engagement factor. If the resulting Stouffer’s *z* corresponded to a probability level less than 0.01, the null hypothesis of no effect was rejected. This methodology has been used in other systematic reviews, including by Yap and colleagues (2014; 2015), to assess whether associations between variables are reliable. For information on how *p*-values were extracted and selected for analysis, see Supplemental Information 2.

### Assessing risk of bias

Critical appraisal of quantitative studies was conducted using the Cochrane Risk of Bias Tool (Higgins & Green, 2008), which involved assessing for adequate sequence generation, allocation concealment, blinding of assessors to treatment conditions, the inclusion of intention to treat analyses and assessment of potential confounders. Risk of bias for all included studies was assessed by two authors (SF and BS) using a standardised, pilot-tested extraction sheet. Disagreements were resolved through discussion between SF and BS.

## Results

From 13,877 studies identified in the initial searches of published literature, 358 were full-text screened and 335 articles were excluded (see Fig. 1 for reasons). The remaining 23 articles were included, comprising 21 separate studies. These studies were organised into two categories: (1) studies that measured and described predictors of engagement and (2) studies that attempted to increase parent engagement using targeted engagement methods (see summary in Table 2 below, and Tables in Supplemental Information 3, Supplemental Information 4 and Supplemental Information 5 for detailed study characteristics and all extracted data). Separate articles from the same study that reported results on different categories were included in the current review. These studies have been summarised below with a mixture of narrative review and Stouffer’s *p* analysis. Due to many studies obtaining several unclear bias ratings, the quality of the included studies remains inconclusive. As illustrated in Table 3, the maximum number of low bias ratings for any individual study was three (Bjørknes, Jakobsen & Nærde, 2011; Bjørknes & Manger, 2013; Hellenthal, 2009). Refer to Table 3 for a summary of results from the risk of bias assessment (for more details, see Table in Supplemental Information 6).

**Table 2:** Summary of study results, overall participant numbers and percentage of engaged parents by stage of engagement.

Studies, identified by first author	Participants	Parenting intervention name^a^	Intent to enrol	Enrolment	Ongoing engagement	Engagement enhancement strategies (EES)	Main findings^b^
Aalborg et al. (2012)	Parents of adolescents aged 11–12 years (*n* = 614)	Strengthening Families Program: For Parents and Youth 10–14 (SFP) & Family Matters (FM)	n/a	n/a	SFP *M* = 5.2 (choice), *M* = 4.8 (assigned) FM *M* = 3.3 (choice), *M* = 3.4 (assigned)^q^	Parents were able to choose which program to attend versus being assigned to a program	**EES** Families who chose FM completed the program in a shorter period of time and those who chose SFP attended more sessions
Baker, Arnold & Meagher (2011)	Parents of preschool aged children (*n* = 106)	Incredible Years	n/a	48.1%^h^	61%^r^	n/a	**Enrolment** PR *p* > .01 1/2PH, PMHS, CMHS n/s **Attendance** 1/2PH *p* < .01 PR, PMHS, CMHS n/s
Bjørknes, Jakobsen & Nærde (2011)	Parents of children aged 3–9 years at risk of developing conduct problems (*n* = 96)	Parent Management Training—The Oregon Model	n/a	n/a	n/a	Strategies were recruitment via: (1) professionals from regular public services, (2) community information meetings, and (3) staff from the recruitment team	**EES** Information meetings were the most cost-effective strategy and the highest proportion of the sample was recruited via these meetings
Bjørknes & Manger (2013)	Parents of children aged 3–9 years at risk of developing conduct problems (*n* = 50, those offered intervention)	Parent Management Training—The Oregon Model	n/a	n/a	*M* = 10.75^q^ 66%^s^	See Bjørknes, Jakobsen & Nærde (2011)	Analysis was limited to child behaviour outcomes of those who attended more than 50% of intervention
Brody et al. (2006)	Parents of children aged 11 years (*n* = 172, those offered intervention)	Strong African American Families	n/a	n/a	65%^s^	n/a	**Attendance** FS *p* < .05 PMHS n/s
Byrnes et al. (2012)^c^	Parents of adolescents aged 11–12 years (*n* = 214)	Family Matters	47.2%^e^	61.0%^j^	n/a	Parents were able to choose which program to attend versus being assigned to a program	**Intent** P age, C sex n/s **Enrolment** P age *p* < .05 C sex n/s
Calam et al. (2008)^c^	Any parents that signed up to the study associated with a public TV broadcast of program (*n* = 723)	Driving Mum and Dad Mad	n/a	n/a	*M* = 5 (recruitment drive 1) *M* = 4.29 (recruitment drive 2)^q^	Standard condition; received weekly email reminding them to watch TV series. Enhanced condition: received emails plus self-help workbook and extra web support	**Attendance** CMHS *p* < .05 **ER** More parents maintained attendance in the standard condition versus the enhanced condition. However, both groups attended the same average number of sessions
Carpentier et al. (2007)^c^	Parents of children in 7th grade and under age of 15 years (*n* = 596)		65%^g^	62%^p^	*M* = 5.3^q^	Bilingual letter using Health Belief Model and cultural sensitivity, and follow up phone call	**Enrolment** 1/2PH, FS, PR, P ed, CMHS (internal and external symptoms) n/s **Attendance** 1/2PH, FS, PR, P ed, P occ, CMHS (internal and external symptoms) n/s **EES** Participation rates higher than reported rates of minority-focused trials which did not emphasize cultural sensitivity
Eisner & Meidert (2011)^c^	Parents of children in 1st grade (*n* = 257)	Triple P	n/a	31.3%^k^	18.6%^s^ 26.8%^t^	Practitioners were responsible for recruitment through schools	**Enrolment** FS *p* < .01 P oc *p* < .001 1/2PH, CMHS n/s **Attendance** FS *p* < .05 1/2PH, CMHS n/s **EES** Practitioner-led recruitment into parent training can achieve enrolment and participation rates that are comparable to researcher-led trials
Fleming et al. (2015)	Parents of children in 8th grade (*n* = 213)	Common Sense Parenting	n/a	70% (6-session version), 79% (8-session version)^i^	21% (CSP), 17% (CSP+)^s^	n/a	**Enrolment** PES *p* < .05 C age *p* < .05 1/2PH, P age, P sex, PR, C sex, CMHS (strengths and difficulties, child emotional symptoms) n/s **Attendance** C sex *p* < .01 1/2PH, P age, PR, P ed, C age, CMHS (conduct problems, emotional symptoms) n/s
Garvey et al. (2006)	Parents or legal guardians of children aged 2–4 years (*n* = 292)	The Chicago Parent Program	n/a	34.9%^p^	*M* = 4.3^q^	n/a	**Attendance** CMHS *p* < .05 1/2PH, P age, PR, P ed, P occ, PMHS (stress, depression) n/s
Heinrichs et al. (2005)	Parents of children aged 2.6–6 years (*n* = 282)	Triple P	n/a	31%^p^	89%^s^	n/a	**Enrolment** 1/2PH *p* < .05 FS, P age, POS n/s
Heinrichs (2006)	Parents of children aged 2.6–6 years (*n* = 197)	Triple P	n/a	36%^p^	*M* = 7.0 h^q^ 85%^s^	Two incentives for participants; (1) monetary incentives, and (2) group versus individual setting	**EES** Setting (group or individual) did not significantly affect engagement
Helfenbaum-Kun & Ortiz (2007)	Fathers of children aged 3–5 years (*n* = 39)	Incredible Years	n/a	85%^l^	30%^s^	(1) Parents recruited in Head Start parent meetings, (2) distribution of bilingual advertisements, and (3) father-only parent training groups	**EES** Initial interest was strong. However, attendance and dropout was high
Hellenthal^d^(2009)	Parents of children aged 2–12 years (*n* = 72)	Barkley (1997)’s Behavioural Parent Training	n/a	n/a	65.28%^s^	n/a	**Attendance** P age, P ed, PMHS, CMHS n/s
Mauricio et al. (2014)	Parents of children aged 11–14 years (*n* = 353)	Bridges to High School	n/a	n/a	n/r	n/a	**Attendance** PMHS *p* < .05 CMHS *p* < .05
Mian, Eisenhower & Carter (2015)^c^	Parents of children aged 11–71 months who were receiving nutritional assistance (*n* = 101)	Not named, once off anxiety prevention seminar	.6% (control) & 49% (ER)^f^	n/a	.4% (control group) & 13% (ER group)^s^	ER included: (1) community endorsement (letter from WIC program director), (2) follow up phone call, and (3) letter explaining how researchers had matched parents’ time preferences	**Intent** P occ *p* = .07 & *p* < .05 P age, P sex, PR, P ed, PMHS, C age, C sex, CMHS n/s **EES** ER was associated with both intent and attendance
Miller et al. (2011)	Parents of adolescents aged 11–12 years (*n* =614)	Strengthening Families Program: For Parents and Youth 10–14 (SFP) & Family Matters (FM)	n/a	n/a	SFP *M* = 5.2 (choice), *M* = 4.8 (assigned) out of 7 sessions FM *M* = 3.3 (choice), *M* = 3.4 (assigned) out of 4 booklets^q^	Parents were able to choose which program to attend versus being assigned to a program	**EES** Family who chose Family Matters completed the program in a shorter period of time and those who chose SFP attended more sessions
Nordstrom, Dumas & Gitter (2008)^d^	Parents of children aged 3–6 years (*n* = 347)	Parenting our Children to Excellence	62.2%^g^	33%^j^	56.5%^s^	n/a	**Intent** 1/2PH, P age, PR, P ed, P occ, C age, C sex, CMHS (ADHD, ODD) n/s **Enrolment** P age *p* < .004 CMHS (ODD) *p* < .001 1/2PH, PR, P ed, P occ, C age, C sex, CMHS (ADHD) n/s **Attendance** PES *p* = .026 1/2PH, P age, PR, P occ, C age, C sex, CMHS (ADHD, ODD) n/s
Plueck et al. (2010)	Parents of children aged 3–6 years (*n* = 2, 123)	Prevention Program for Externalising Problem Behaviour	n/a	63.8%^m^	*M* = 7.5^q^ 81.1%^s^	n/a	**Enrolment** CMHS (externalising symptoms) *p* < .044 1/2PH, P age, C age, C sex, CMHS (internalising symptoms) n/s **Attendance** 1/2PH, P age, C age, C sex, CMHS n/s
Reedtz et al. (2011)	Parents of children aged 2–8 years, who scored below 90th percentile on ECBI (*n* = 189)	Incredible Years	n/a	89.5%^n^	n/a	n/a	**Enrolment** CMHS *p* < .001 P ed n/s
Skärstrand et al. (2009)	Parents of children in grades 6–9 (*n* = 388)	Strengthening Families Program: For Parents and Youth 10–14	n/a	47%^p^	*M* = 5.19^q^	n/a	**Enrolment** FS, P age, P sex, PR, P ed, C sex, CMHS n/s **Attendance** PR *p* < .01 FS, P age, P ed, P occ, C sex, CMHS n/s
Winslow et al. (2009)	Divorced mothers of children aged 9–12 years (*n* = 325)	Not named, program of recently divorced mothers	n/a	73.%^l^	*M* = 12.1^q^	n/a	**Enrolment** CMHS *p* > .05 PR, PES, PMHS n/s **Attendance** PES *p* < .05 PR, PMHS, CMHS n/s

**Notes.**

Abbreviations P age Parent Age P sex Gender of Parent P ed Parent Education Status P occ Parent Employment/Occupation Status PR Parent Race/Ethnicity PMHS Parent Mental Health Status C age Child Age C sex Child Gender CMHS Child Mental Health Symptoms FS Family Structure 1/2 PH One or Two Parent Households n/a not applicable to study n/r not reported in published article n/s non-significant *p* value *p* < .05 significant *p*-value

^a^For more information about each parenting intervention, see Supplemental Information 4. ^b^Main findings column lists the findings for each of the 11 categories of predictors across three stages of engagement (for other predictors, see Supplemental Information 5), and/or the findings from studies that trialled enhanced recruitment methodologies. ^c^Studies that trialled enhanced recruitment methods and measured predictors of engagement. ^d^All studies included an RCT study design except for Nordstrom, Dumas & Gitter (2008) who employs a correlational study design and Hellenthal (2009) who employs a quasi-experimental design. **Intent to Enroll Rates** ^e^Agreed to participate (*n* = 1). ^f^Returned RSVP (*n* = 1). ^g^Asked question which pertained to intent i.e. do you intend to enroll? (*n* = 2). **Enrollment Rates** ^h^Attended first session (*n* = 1). ^i^Attended any session (*n* = 1). ^j^Returning consent form or completing baseline assessment (n=2). ^k^ Enrolled in parenting program (*n* = 1). ^l^ Agreed to participate (*n* = 2). ^m^Accepted invitation to complete pre-test (*n* = 1). ^n^Continued to participate post completion of pre-test (*n* = 1). ^p^Did not clearly define enrollment (*n* = 5). **Attendance Rates** ^q^Average number of sessions attended (*n* = 10). ^r^Total percentage of sessions attended by parents (*n* = 1). ^s^Percentage of parents that attended the minimum number of required sessions (between 50–100% of sessions offered) ( *n* = 10). ^t^ Percentage of parents that attended at least one session (*n* = 1).

**Table 3:** Summary of risk of bias for quantitative studies.

Studies, identified by first author	Selection bias	Performance bias	Detection bias	Attrition bias	Reporting bias	Other bias	Total *n* of low risk
Aalborg et al. (2012)	Unknown	**High**	Unknown	Unknown	Unknown	Unknown	0
Baker, Arnold & Meagher (2011)	Unknown	Unknown	Unknown	Unknown	Unknown	Unknown	0
Bjørknes, Jakobsen & Nærde (2011)	**Low**	**Low**	Unknown	**Low**	Unknown	Unknown	3
Bjørknes & Manger (2013)	**Low**	**Low**	Unknown	**Low**	Unknown	Unknown	3
Brody et al. (2006)	Unknown	Unknown	Unknown	Unknown	Unknown	Unknown	0
Byrnes et al. (2012)	Unknown	**High**	Unknown	Unknown	Unknown	Unknown	0
Calam et al. (2008)	Unknown	**Low**	**Low**	Unknown	Unknown	Unknown	2
Carpentier et al. (2007)	Unknown	Unknown	Unknown	**High**	Unknown	**High**	0
Eisner & Meidert (2011)	Unknown	Unknown	Unknown	Unknown	Unknown	Unknown	0
Fleming et al. (2015)	Unknown	Unknown	**Low**	Unknown	Unknown	Unknown	1
Garvey et al. (2006)	Unknown	Unknown	Unknown	Unknown	Unknown	Unknown	0
Heinrichs et al. (2005)	**High**	**High**	Unknown	Unknown	Unknown	Unknown	0
Heinrichs (2006)	Unknown	**High**	Unknown	Unknown	Unknown	Unknown	0
Helfenbaum-Kun & Ortiz (2007)	Unknown	Unknown	Unknown	Unknown	Unknown	Unknown	0
Hellenthal (2009)	**Low**	**Low**	**Low**	Unknown	Unknown	Unknown	3
Mauricio et al. (2014)	Unknown	Unknown	Unknown	Unknown	Unknown	Unknown	0
Mian, Eisenhower & Carter (2015)	Unknown	Unknown	Unknown	**Low**	Unknown	Unknown	1
Miller et al. (2011)	Unknown	**High**	Unknown	Unknown	Unknown	Unknown	0
Nordstrom, Dumas & Gitter (2008)	Unknown	Unknown	Unknown	**High**	Unknown	Unknown	0
Plueck et al. (2010)	Unknown	Unknown	Unknown	Unknown	Unknown	Unknown	0
Reedtz et al. (2011)	Unknown	Unknown	Unknown	Unknown	Unknown	Unknown	0
Skärstrand et al. (2009)	Unknown	Unknown	Unknown	Unknown	Unknown	Unknown	0
Winslow et al. (2009)	Unknown	Unknown	Unknown	Unknown	Unknown	Unknown	0

**Notes.**

Bold text indicates low bias rating.

### Study characteristics

#### Design

Of the 21 studies included, most involved universal prevention programs, and were conducted in the USA (see Table 4). The most common mental health problem targeted was externalising disorders (*n* = 13, i.e., conduct disorder; Baker, Arnold & Meagher, 2011; Bjørknes, Jakobsen & Nærde, 2011; Garvey et al., 2006; Heinrichs et al., 2005; Heinrichs, 2006; Helfenbaum-Kun & Ortiz, 2007; Hellenthal, 2009; Mauricio et al., 2014; Nordstrom, Dumas & Gitter, 2008; Plueck et al., 2010; Reedtz et al., 2011; Skärstrand et al., 2009; Winslow et al., 2009). Nineteen studies were randomised controlled trials (RCT), while one study employed a correlational study design (Nordstrom, Dumas & Gitter, 2008) and another a quasi-experimental design (Hellenthal, 2009). Although the inclusion criteria allowed for a broader range of study designs, only experimental trials met the additional inclusion criteria (i.e., studies assessing parent engagement). The included studies can be categorised into two not-mutually-exclusive groups: (1) studies measuring predictors of engagement (*n* = 17; Baker, Arnold & Meagher, 2011; Brody et al., 2006; Byrnes et al., 2012; Calam et al., 2008; Carpentier et al., 2007; Eisner & Meidert, 2011; Fleming et al., 2015; Garvey et al., 2006; Heinrichs et al., 2005; Hellenthal, 2009; Mauricio et al., 2014; Mian, Eisenhower & Carter, 2015; Nordstrom, Dumas & Gitter, 2008; Plueck et al., 2010; Reedtz et al., 2011; Skärstrand et al., 2009; Winslow et al., 2009), and (2) studies that evaluated engagement methodologies (*n* = 9; Aalborg et al., 2012; Bjørknes, Jakobsen & Nærde, 2011; Byrnes et al., 2012; Calam et al., 2008; Carpentier et al., 2007; Eisner & Meidert, 2011; Heinrichs, 2006; Helfenbaum-Kun & Ortiz, 2007; Mian, Eisenhower & Carter, 2015). Some studies had dual aims (i.e., evaluation of an engagement methodology and measurement of predictors; Calam et al., 2008; Carpentier et al., 2007; Eisner & Meidert, 2011; Mian, Eisenhower & Carter, 2015).

**Table 4:** Summary of study characteristics.

	Number of studies (*n*)	%
Participant characteristics		
*Type of prevention program*		
Universal	11	52.3
Selective	8	38.2
Indicated	2	9.5
*Country*		
USA	13	61.9
Europe	8	38.1
*Mean age of children at recruitment*		
Preschool (0–5 years)	8	38.1
Primary school (>5–11 years)	4	19.0
Adolescence (>11–18 years)	9	42.9
*Parent gender*		
>60% female	20	95.2
>60% male	1	4.8
Program characteristics		
*Focus of intervention*		
Prevention of substance use behaviours	3	14.3
Prevention of internalising disorders	1	4.8
Prevention of externalising disorders	13	61.9
Prevention of other mental health disorders	4	19.0
*Delivery format*^a^		
Group sessions (parent/family)	16	76.2
Individual sessions (parent/family)	2	9.5
Mix of group and home visits/phone calls	2	9.5
Work books	2	9.5
Technology-based program	1	4.8
*Total number of intervention sessions*^b^		
1 to 5	1	4.8
6 to 9	20	95.2
10 or more	4	19.0
*Direct intervention with child*		
Yes	6	28.5
No	15	71.5
Method characteristics		
*Design*		
Randomised controlled trials	19	90.5
Non-randomised experimental trials	2	9.5
*Aim*^a^		
Evaluated recruitment methodologies	9	42.8
Measuring predictors of engagement	17	80.9
*Recruitment methods*		
Mail out or generic advertisements	6	28.6
Mail out plus phone call	3	14.3
Mail out plus researchers spending time at centres	9	42.8
Personal invitations	1	4.8
Pre-screeners	2	9.5
*Stage of engagement measured*^a^		
Intent to enrol	6	28.5
Enrolment	18	85.7
Attendance	15	95.2

**Notes.**

a Percentage does not equal 100 because studies could fall into multiple categories.

b Five RCTs included two or more different versions of the parenting program being researched.

Therefore, the percentage does not equal 100 because the different versions of the programs could have different numbers of sessions.

#### Participants and recruitment methods

Participants in all studies were parents of children or adolescents; these parents were typically mothers or female caregivers. Only one study actively sought to engage fathers in a preventive parenting program (Helfenbaum-Kun & Ortiz, 2007). The average number of participants across all studies was *n* = 262, but ranged widely from 39 to 723 participants. These participants were recruited in several ways, with the most common method being the mail-out of a letter or advertisements by the recruiting organisation (i.e., day care centre, school, or medical facility; *n* = 18; Aalborg et al., 2012; Baker, Arnold & Meagher, 2011; Brody et al., 2006; Byrnes et al., 2012; Calam et al., 2008; Carpentier et al., 2007; Eisner & Meidert, 2011; Fleming et al., 2015; Garvey et al., 2006; Heinrichs et al., 2005; Heinrichs, 2006; Helfenbaum-Kun & Ortiz, 2007; Hellenthal, 2009; Mauricio et al., 2014; Nordstrom, Dumas & Gitter, 2008; Reedtz et al., 2011; Skärstrand et al., 2009; Winslow et al., 2009). Three of these studies also included a telephone follow-up after the letter had been sent out (Brody et al., 2006; Carpentier et al., 2007; Winslow et al., 2009). Nine studies used letters in conjunction with researchers spending time at the facilities to answer questions about the program and/or conducting a presentation at parent-teacher interview nights (Baker, Arnold & Meagher, 2011; Eisner & Meidert, 2011; Fleming et al., 2015; Garvey et al., 2006; Heinrichs et al., 2005; Heinrichs, 2006; Helfenbaum-Kun & Ortiz, 2007; Nordstrom, Dumas & Gitter, 2008; Skärstrand et al., 2009). One study (reported in two articles) recruited through personal invitation to the study, either through researcher or professional networks (Bjørknes, Jakobsen & Nærde, 2011; Bjørknes & Manger, 2013). Two studies used pre-screening measures to provide individualised feedback to parents and offered the program to those parents whose children were at increased risk for developing mental health problems (Mian, Eisenhower & Carter, 2015; Plueck et al., 2010). Only one study (Carpentier et al., 2007) explicitly used known psychological theories to guide their recruitment methods. That is, the Health Belief Model was utilised to construct a letter that was expected to be more motivating than a general recruitment letter or flyer.

#### Interventions

Inclusion criteria required that programs included in the current review be preventive; that is, studies either excluded participants with diagnosable difficulties identified through rigorous assessment (e.g., structured clinical interviews), or assumed that participants did not have current or previous clinically diagnosable disorders (e.g., recruited a community-based sample that was not rigorously screened). Studies were included if children were assessed as ‘at risk’ of developing a mental health disorder in the future, and coded as indicated (*n* = 2; Hellenthal, 2009; Plueck et al., 2010) or selective (*n* = 8; Brody et al., 2006; Bjørknes, Jakobsen & Nærde, 2011; Bjørknes & Manger, 2013; Carpentier et al., 2007; Mauricio et al., 2014; Mian, Eisenhower & Carter, 2015; Reedtz et al., 2011; Skärstrand et al., 2009; Winslow et al., 2009) prevention programs, based on each study’s chosen description. Seventeen different programs were evaluated in the 21 included studies (see Table in Supplemental Information 3 for details), of which 14 were face-to-face group programs, with the number of sessions ranging from 1 (Mian, Eisenhower & Carter, 2015) to 18 (Bjørknes, Jakobsen & Nærde, 2011; Bjørknes & Manger, 2013). Four of these programs had the target child or adolescent involved in the program (Aalborg et al., 2012; Brody et al., 2006; Byrnes et al., 2012; Carpentier et al., 2007; Fleming et al., 2015; Mauricio et al., 2014; Miller et al., 2011) and one included education sessions for the target child’s school teachers (Plueck et al., 2010). One group program also involved four weekly telephone check-ins with parents (Heinrichs et al., 2005; Heinrichs, 2006; Eisner & Meidert, 2011). These phone calls were voluntary and designed to increase the amount of therapeutic and intervention time for parents. One program involved parents working through four booklets at home with regular telephone calls to collect further data from participating parents (Aalborg et al., 2012; Byrnes et al., 2012; Miller et al., 2011). The remaining program was classified as a technology-assisted program and entailed parents who were enrolled in the first recruitment drive watching six 30-minute videos and parents recruited in the second recruitment drive watching five 60-minute videos (Calam et al., 2008).

#### Enrolment and ongoing engagement rates

Enrolment and ongoing engagement rates were difficult to synthesise across studies, due to: (1) differing definitions of enrolment, ongoing engagement and completion of programs, (2) an inability to obtain the number of eligible parents in some studies due to recruitment methods such as advertisements in newspapers, and (3) inadequate reporting of details about enrolment and ongoing engagement rates in some studies. For the articles with adequate reporting of the total number of eligible parents and subsequent enrolments (*n* = 14: Baker, Arnold & Meagher, 2011; Byrnes et al., 2012; Carpentier et al., 2007; Eisner & Meidert, 2011; Fleming et al., 2015; Garvey et al., 2006; Heinrichs et al., 2005; Heinrichs, 2006; Helfenbaum-Kun & Ortiz, 2007; Nordstrom, Dumas & Gitter, 2008; Plueck et al., 2010; Reedtz et al., 2011; Skärstrand et al., 2009; Winslow et al., 2009), the *actual enrolment* rates varied between 30% and 85%. The way studies measured ongoing engagement could be categorised into four groups: (1) average number of sessions attended by parents (*n* = 10: Aalborg et al., 2012; Bjørknes & Manger, 2013; Calam et al., 2008; Carpentier et al., 2007; Garvey et al., 2006; Heinrichs, 2006; Miller et al., 2011; Plueck et al., 2010; Skärstrand et al., 2009; Winslow et al., 2009); (2) percentage of parents that attended the minimum number of required sessions (*n* = 10: Brody et al., 2006; Eisner & Meidert, 2011; Fleming et al., 2015; Heinrichs et al., 2005; Heinrichs, 2006; Helfenbaum-Kun & Ortiz, 2007; Hellenthal, 2009; Mian, Eisenhower & Carter, 2015; Nordstrom, Dumas & Gitter, 2008; Plueck et al., 2010); (3) total percentage of sessions attended by parents (*n* = 1: Baker, Arnold & Meagher, 2011); and (4) percentage of parents that attended at least one session (*n* = 1: Eisner & Meidert, 2011).

Several studies (*n* = 4: Baker, Arnold & Meagher, 2011; Carpentier et al., 2007; Eisner & Meidert, 2011; Skärstrand et al., 2009) documented how many parents attended the first session and subsequently tracked these parents’ attendance across the program. For these studies, there was a trend for parents to engage in the first session and then not return, with the average number of sessions engaged in varying between three and seven. There appeared to be a trend for parents to engage in an average of four or five sessions when the program contained less than ten sessions (*n* = 4: Aalborg et al., 2012; Calam et al., 2008; Carpentier et al., 2007; Heinrichs, 2006), but the average number of sessions engaged in jumped to seven or eight sessions when the program contained ten sessions or more (*n* = 6: Bjørknes, Jakobsen & Nærde, 2011; Bjørknes & Manger, 2013; Garvey et al., 2006; Plueck et al., 2010; Skärstrand et al., 2009; Winslow et al., 2009).

### Synthesis of results: predictors of parental engagement across stages of engagement

Seventeen studies measured the factors that predict parent engagement (Baker, Arnold & Meagher, 2011; Brody et al., 2006; Byrnes et al., 2012; Calam et al., 2008; Carpentier et al., 2007; Eisner & Meidert, 2011; Fleming et al., 2015; Garvey et al., 2006; Heinrichs et al., 2005; Hellenthal, 2009; Mauricio et al., 2014; Mian, Eisenhower & Carter, 2015; Nordstrom, Dumas & Gitter, 2008; Plueck et al., 2010; Reedtz et al., 2011; Skärstrand et al., 2009; Winslow et al., 2009). Most studies focused on predictors that could be coded into categories in this review, but isolated studies also looked at how parent cognitions (i.e., parents’ thoughts and beliefs about themselves and the program; Nordstrom, Dumas & Gitter, 2008) and parent-recorded obstacles to engagement (i.e., need for child care, transportation costs) measured at pre-intervention, can predict parent engagement across a preventive parenting program trial (Mian, Eisenhower & Carter, 2015; Nordstrom, Dumas & Gitter, 2008). Although some of these findings appear promising, there is little consistent evidence across studies to support any predictors of parent engagement, at the time of this review. This inconsistency is demonstrated in Table 5, where only one of the 11 categories assessed yielded a significant Stouffer’s *p*, and only for the enrolment stage of engagement.

**Table 5:** Findings from Stouffer’s *p* calculations.

Themes/predictors of engagement	Stages of engagement		
	Intent	Enrolment	Ongoing engagement
***Parent age***			
*n* of studies	3	6	7
*n* of associations	3	6	7
*Stouffer’s p*	.163	.376	.098
***Gender of parent***			
*n* of studies	1	2	2
*n* of associations	1	2	2
*Stouffer’s p*	.500	.500	.500
***Parent race/ethnicity***			
*n* of studies	2	6	8
*n* of associations	2	7	8
*Stouffer’s p*	.500	.020	.156
***Parent education status***			
*n* of studies	2	6	9
*n* of associations	2	6	9
*Stouffer’s p*	.500	.250	.115
***Parent employment status***			
*n* of studies	2	4	5
*n* of associations	3	4	5
*Stouffer’s p*	.035	.061	.500
***Parent mental health status***			
*n* of studies	1	2	7
*n* of associations	1	2	8
*Stouffer’s p*	.500	.592	.361
***Child age***			
*n* of studies	2	3	4
*n* of associations	2	3	4
*Stouffer’s p*	.105	.354	.293
***Child gender***			
*n* of studies	4	5	7
*n* of associations	4	5	7
*Stouffer’s p*	.409	.176	.124
***Child mental health symptoms***			
*n* of studies	3	8	14
*n* of associations	5	13	19
*Stouffer’s p*	.541	**.004**	.028
***Family structure***			
*n* of studies	Nil	4	4
*n* of associations		4	4
*Stouffer’s p*		.122	.050
***One- or two- parent households***			
*n* of studies	1	7	8
*n* of associations	1	7	8
*Stouffer’s p*	.500	.328	.121

**Notes.**

Bold text indicates significance (*p* < .01).

#### Intent to enrol

Only four studies measured potential predictors of parents’ *intent to enrol* (Byrnes et al., 2012; Mian, Eisenhower & Carter, 2015; Nordstrom, Dumas & Gitter, 2008; Plueck et al., 2010). Stouffer’s *p* analyses indicated a lack of evidence to show a reliable association between all investigated predictors and parents’ *intent to enrol*. Additional factors associated with less *intent to enrol*, as found in these four studies, included: neighbourhood social burden as defined by the Department of Youth Welfare (Plueck et al., 2010), higher levels of neighbourhood unemployment (Byrnes et al., 2012) and teachers perceiving a higher need for assistance (Plueck et al., 2010). Conversely, one study found an association between the following predictors and a greater *intent to enrol*: a parent perceiving greater benefits for participation and fewer scheduling barriers (Nordstrom, Dumas & Gitter, 2008).

#### Enrolment

A total of 11 studies measured the predictors of parents enrolling in a preventive parenting program (Baker, Arnold & Meagher, 2011; Byrnes et al., 2012; Carpentier et al., 2007; Eisner & Meidert, 2011; Fleming et al., 2015; Heinrichs et al., 2005; Nordstrom, Dumas & Gitter, 2008; Plueck et al., 2010; Reedtz et al., 2011; Skärstrand et al., 2009; Winslow et al., 2009). Based on Stouffer’s *p* calculations, only 1 of the 11 categories (child mental health symptoms) demonstrated a reliable association with *enrolment* (Stouffer’s *z* = −2.63, *p* < .01). Studies found that higher levels of parent-reported child mental health symptoms were associated with greater parental *enrolment* (Nordstrom, Dumas & Gitter, 2008; Plueck et al., 2010; Reedtz et al., 2011; Skärstrand et al., 2009; Winslow et al., 2009). Additionally, isolated studies found the following predictors to be associated with increased *enrolment:* parents having more social supports, both individually and in the community, higher parental self-efficacy and higher perceived benefits of the program (Baker, Arnold & Meagher, 2011; Eisner & Meidert, 2011; Nordstrom, Dumas & Gitter, 2008).

#### Ongoing engagement

*Ongoing engagement* was the most commonly studied stage of engagement, with 15 studies assessing their predictors (Baker, Arnold & Meagher, 2011; Brody et al., 2006; Calam et al., 2008; Carpentier et al., 2007; Eisner & Meidert, 2011; Fleming et al., 2015; Garvey et al., 2006; Heinrichs et al., 2005; Hellenthal, 2009; Mauricio et al., 2014; Mian, Eisenhower & Carter, 2015; Nordstrom, Dumas & Gitter, 2008; Plueck et al., 2010; Skärstrand et al., 2009; Winslow et al., 2009). Despite the larger evidence base, Stouffer’s *p* analyses revealed no reliable associations with investigated predictors. The most commonly studied predictor, child mental health symptoms, was found in a limited number of studies to be significantly associated with parental *ongoing engagement* (Baker, Arnold & Meagher, 2011; Calam et al., 2008; Garvey et al., 2006; Hellenthal, 2009; Mauricio et al., 2014), but Stouffer’s *p* was not significant (Stouffer’s *z* =  − 1.91, Stouffer’s *p* = 0.028). Four of the 13 studies that measured child mental health symptoms (Baker, Arnold & Meagher, 2011; Calam et al., 2008; Garvey et al., 2006; Hellenthal, 2009) found higher levels of child mental health symptoms were associated with better ongoing engagement, while Mauricio and colleagues (2014) found higher levels of child externalising behaviours to be associated with poorer *ongoing engagement*.

### Synthesis of results: effects of engagement enhancement methods

Nine studies attempted to increase engagement through ‘engagement enhancement methods’ (Aalborg et al., 2012; Bjørknes, Jakobsen & Nærde, 2011; Byrnes et al., 2012; Calam et al., 2008; Carpentier et al., 2007; Eisner & Meidert, 2011; Heinrichs, 2006; Helfenbaum-Kun & Ortiz, 2007; Mian, Eisenhower & Carter, 2015). Five of the nine studies tested engagement enhancement methods using randomised controlled trials (Aalborg et al., 2012; Byrnes et al., 2012; Calam et al., 2008; Heinrichs, 2006; Mian, Eisenhower & Carter, 2015), while a further four used engagement enhancement methods to recruit all participants (Bjørknes, Jakobsen & Nærde, 2011; Bjørknes & Manger, 2013; Carpentier et al., 2007; Eisner & Meidert, 2011; Helfenbaum-Kun & Ortiz, 2007).

The studies that randomised participants into different engagement methods had varied results. Studies randomised parents into either paid versus unpaid conditions (Heinrichs, 2006), web-enhanced versus standard video viewing conditions (Calam et al., 2008), or enhanced recruitment (using personalised letters and phone calls) versus recruitment as usual (Mian, Eisenhower & Carter, 2015). Heinrichs (2006) randomised ‘parents’ and ‘child care centres’ into paid and non-paid conditions. Heinrichs found that parents’ intent to enrol was significantly increased when offered payment for attending sessions, however actual enrolment and attendance did not differ between paid and unpaid conditions. Additionally, although Heinrichs hypothesised that offering payment would increase engagement from migrant parents, the research demonstrated that the opposite was true: native-born parents were more likely to engage in the program when offered payment than those who were first-generation migrants (Heinrichs, 2006). In Mian and colleagues’ (2015) enhanced recruitment study for a once-off seminar, parents in the enhanced condition (including personalised letters and follow-up phone calls) were significantly more likely to intend to enrol, and this intent was found to be related to ongoing engagement. Furthermore, Calam and colleagues (2008) investigated whether an internet-enhanced version of a video-based program “Driving Mum and Dad Mad’ would affect parents’ ongoing engagement (recorded as number of videos watched). The internet-enhanced version included parents having access to a website with further information and activities related to each weekly video. Parents watched on average the same number of videos regardless of the condition they were assigned to.

Two RCT studies (reported in three articles) assessed the likelihood that ongoing engagement would increase if parents were given a choice of program (Aalborg et al., 2012; Byrnes et al., 2012; Miller et al., 2011). These studies used preventive parenting programs with significantly different presentation styles. The first, Family Matters (FM), required parents to complete four booklets at home with their adolescents, and families received weekly phone calls from the research team (Aalborg et al., 2012; Byrnes et al., 2012; Miller et al., 2011). The second, the Strengthening Families Program (SFP), required families to attend seven two-hour weekly group sessions at a medical facility (Aalborg et al., 2012; Byrnes et al., 2012; Miller et al., 2011). These studies found that compared to parents who were randomised to the corresponding no-choice condition, parents in the choice condition who chose FM completed the booklets over a significantly shorter period, and parents who chose SFP attended more sessions (Aalborg et al., 2012; Miller et al., 2011). In addition, parents who self-selected into the two different programs demonstrated some significantly different characteristics. Parents who chose the FM program were more likely to be educated, whereas parents who chose the SFP program described their adolescent’s behaviour problems as more severe (Miller et al., 2011). Miller and colleagues (2011) hypothesised that this difference between parents’ program choice could be because parents who rated their teenager’s behaviour problems as more severe felt they needed a more personalised level of intervention (SFP).

Finally, four studies used engagement enhancement methodologies to recruit all participants (Bjørknes, Jakobsen & Nærde, 2011; Bjørknes & Manger, 2013; Carpentier et al., 2007; Eisner & Meidert, 2011; Helfenbaum-Kun & Ortiz, 2007). One study discussed the different number and type of participants recruited from (1) local information meetings which included talks by cultural leaders, (2) public service professionals, and (3) the recruitment team’s personal and professional networks, and compared this to the number of hours required to recruit these participants (Bjørknes, Jakobsen & Nærde, 2011). The authors reported that local information meetings were the most successful recruitment approach, with 57% of their sample recruited through these meetings (Bjørknes, Jakobsen & Nærde, 2011). In addition, these meetings were also the most cost-effective and least time-intensive approach per participant (Bjørknes, Jakobsen & Nærde, 2011). Recruitment through public service professionals was seen to be the least effective recruitment approach, accounting for less than 15% of recruited participants (Bjørknes, Jakobsen & Nærde, 2011). Further analysis revealed that parents recruited from local information meetings and the recruitment team’s networks had significantly poorer Norwegian language skills (Bjørknes, Jakobsen & Nærde, 2011).

The remaining three studies that used enhanced strategies to recruit all participants included writing personal letters to parents in their own language, promoting programs via meetings at the schools or child care centres, and making phone calls to parents using experienced interpreters. These studies were found to have enrolment rates of 31.3% (Eisner & Meidert, 2011), 62% (Carpentier et al., 2007) and 85%, respectively (Helfenbaum-Kun & Ortiz, 2007). Eisner & Meidert (2011) reported similar levels of ongoing engagement in comparison to other preventive parenting programs that did not use enhanced recruitment strategies, whilst Carpentier and colleagues (2007) and Helfenbaum-Kun & Ortiz (2007) reported a large proportion of parents dropping out of the study, either before attending the first session or during the program.

## Discussion

This review aimed to synthesise the predictors of engagement and investigate the effectiveness of strategies employed to date to increase parental engagement. Due to the limited number of articles and the substantial variations in their methodologies, a meta-analysis was not conducted, therefore the findings discussed should be interpreted with caution. The following discussion will provide a summary of the evidence found, the limitations to this review and suggestions for future research.

### Summary of evidence

#### Predictors of parental engagement

The current review found limited consistent evidence for factors associated with parental engagement in preventive parenting programs. Interestingly, individual characteristics such as gender and indicators of socio-economic position (SEP; such as family structure, one- or two-parent households and parent education) appeared to have limited to no support in predicting parental engagement across all stages of engagement. This is consistent with Chacko and colleagues’ (2016) finding of limited support for socio-economic status (SES) in their larger review of all programs involving Behavioural Parent Training. Several potential reasons could account for this finding, including the different methods of measurement of SEP across studies, or a lack of variability in the parents engaging in these programs (i.e., only a small percentage of engaged parents come from low SEP backgrounds). Alternatively, it may be the factors associated with lower SEP, rather than SEP itself, that influence intent to enrol. For example, the level of neighbourhood disorganisation appeared to influence a parent’s *intent to enrol* in one study (Byrnes et al., 2012). Neighbourhood disorganisation theory posits that low neighbourhood SEP and residential instability will result in less use of treatment and preventive health care services (Shaw & McKay, 1942; Winstanley et al., 2008). Therefore, it is important to consider the external or societal factors, such as instability and chaos in work, housing, income, family and limited social supports within a community, which may limit a parent’s capacity to engage, in addition to parents’ internal motivation to enrol and attend (Evans & Kantrowitz, 2002).

A secondary aim of this review was to explore the association between the age of the target child and parental engagement. Despite the intention to include studies with a wide age range (0 to 18 years), only 4 included studies measured child age as predictors and no reliable association was found. These studies were also limited in that most included young children from 11 months to 6 years (Mian, Eisenhower & Carter, 2015; Nordstrom, Dumas & Gitter, 2008; Plueck et al., 2010), with Fleming and colleagues (2015) being the only study to include parents of adolescents (children in 8th grade).This finding is consistent with Chacko and colleagues’ (2016) review which examined programs for parents of children aged 2–12 years, and found no significant effect of child age. In addition, given the various definitions of engagement reported across the small number of included studies, it was not possible to provide even a qualitative comparison of patterns of parental engagement between pre-adolescent and adolescent studies. Further research is required to determine if the age of the target child influences a parent’s engagement in preventive parenting programs. This will have important implications for the timing of parenting program delivery, and the need for enhanced engagement strategies if programs are delivered at a stage of child development that is associated with lower rates of parental engagement.

Another possible reason why individual predictors (such as child age or family structure) did not appear to have reliable evidence for all three stages of engagement is that it may be an accumulation of factors, rather than individual standalone factors, that influence parents’ decision to engage. As posited by Evans, Li & Whipple (2013)’s cumulative risk theory, singular risk factors may not demonstrate causation; rather it is a more complex system of inter-related factors that affect parental engagement in preventive parenting programs.

Only one predictor, child mental health symptoms, was found to have reliable evidence in increasing *enrolment*. Parents with children who had increased child mental health symptoms were more likely to enrol. This association was not evident for *ongoing engagement*, suggesting that increased child mental health symptoms may lead a parent to enrol, but once the program has started they may drop out. For example, Mauricio and colleagues (2014, included in this review) found that parents who reported that their child had more externalising behaviours were more likely to enrol or self-select into the parenting program. However, this same group of parents were more likely to terminate their engagement mid-way through the program (Mauricio et al., 2014). This pattern of findings highlights the need to not only examine the different phases of parent engagement separately when trying to identify potential predictors, but also the need for targeted engagement strategies for each distinct phase.

#### Engagement enhancement methods

Despite the difficulties in comparing different engagement enhancement methods used by researchers, the current review found two studies that provide preliminary support for a range of methods modelled on the Health Belief Model and the Theory of Planned Behaviour and Reasoned Action, which could increase parents’ *intent to enrol* and *enrolment*. The methods found to effectively increase parents’ *intent to enrol* and *enrolment* included individualised letters and follow-up phone calls. The Health Belief Model (Rosenstock, 1974) posits that ‘cues to action’ such as reminders, letters and phone calls serve as ways to activate ‘readiness’ in participants and increase the likelihood that they may act. In addition, via the personalised phone calls, researchers can assist parents in overcoming perceived barriers while correcting parents’ misperceptions of susceptibility and severity, where they exist.

Further, the Theory of Planned Behaviour and Reasoned Action (Ajzen, 1991) proposes that people are more likely to agree to partake in healthy behaviours when other respected members of society endorse these programs. Bjorknes and colleagues (2011) applied this principle when recruiting through local community meetings, and found an increase in enrolment from participants who attended the meetings, compared to more traditional methods of recruitment, i.e., researchers’ professional networks. In addition, participants recruited through local community meetings had significantly poorer Norwegian language skills (Bjørknes, Jakobsen & Nærde, 2011). This result could suggest that (1) parents with poorer Norwegian language skills may be more likely to take part because the trusted local leaders were present (and seen to be endorsing the program); and/or (2) lower language skills could serve as a proxy variable for other factors known to influence service access and utilisation, including acculturation, discrimination, past trauma and migration experience (Gee, Walsemann & Takeuchi, 2010). Ensuring these factors are measured in future studies among immigrant populations is an important area of work.

In contrast, engagement enhancement methods, such as individualised letters and phone calls (during recruitment), local meetings, and researchers being available at recruitment sites, appear to be less effective at increasing parents’ *ongoing engagement* in sessions (i.e., Bjørknes, Jakobsen & Nærde, 2011; Mian, Eisenhower & Carter, 2015). This finding is consistent with Ingoldsby’s (2010) review of indicated prevention and early intervention programs, which found engagement interventions that explicitly addressed barriers were effective in increasing initial engagement, but less effective for long-term retention. However, the current review found that parents were more likely to engage in a preventive parenting program if they felt the program was structured to provide them with more perceived control over when and where they engaged in the program. This was demonstrated by the two RCTs which allowed parents to choose the type of program format, which in turn, increased their overall ongoing engagement for both programs (Aalborg et al., 2012; Miller et al., 2011). This finding is consistent with the Theory of Planned Behaviour’s ‘perceived behavioural control’ dimension. Perceived behavioural control refers to a parent’s perceived ease or difficulty of performing the behaviour, which in this case is engaging in the program chosen (Ajzen & Driver, 1991). If the parent perceives the program to be easy to engage in/complete and the parent’s attitude toward the program is favourable, they are more likely to perform the behaviour of engaging in the program in an ongoing manner. Therefore, there is a need for programs that are tailored specifically to different subgroups of parents, providing a range of options to suit parents’ perceived needs and interests.

### Recommendations for future research

#### Clearer definitions and reporting

Based on the Health Belief Model (Rosenstock, 1974), the reduction of perceived barriers, such as providing child care for young children, should increase parents’ engagement in preventive parenting programs. However, many studies included in the current review failed to provide clear and consistent definitions of parental engagement, and there was inadequate reporting of strategies used within programs to increase engagement. Consequently, the effect of provisions of services, such as food, child care and transportation, could not be disentangled. In addition, 32 articles were excluded from this review due to a lack of reporting on how researchers recruited parents (for example, ‘parents were recruited through schools in the area’). Future research should consider clearer definitions of engagement and improved reporting of within-program strategies used to increase ongoing engagement. This will allow for the effectiveness of the provision of these amenities to be further evaluated.

#### Development of engagement strategies based on theories of behaviour

Some of the engagement enhancement strategies reviewed here have shown promise for increasing parents’ intent and enrolment in preventive parenting programs. These strategies could be further developed by reviewing the Health Belief model and the Theory of Planned Behaviour and Reasoned Action. Simple strategies, such as personalised recruitment phone calls or letters, could be easily accommodated into the recruitment methodology of most studies. For example, Carpentier and colleagues (2007) used the Health Belief Model to create a letter that increased parents’ perceived susceptibility, severity and understanding of the potential benefits of the program, and achieved 62% enrolment (of eligible families) in their program. Researchers should also consider the benefits of engaging community leaders to both assist in adapting the programs to be more appropriate and relevant, and to host local meetings to promote the program. These meetings have several benefits including increasing parents’ knowledge of the availability of programs, whilst simultaneously demonstrating legitimacy of these programs through the endorsement of local community leaders (Ajzen & Driver, 1991).

#### Adaptation of programs based on parent need

With the increase in the development of preventive parenting programs, there has been a corresponding increase in the different levels of intensity and formats of program delivery. For example, the current review included studies that delivered parenting programs via individual and group sessions, as well as via booklets, online methods and videos. Enhanced ongoing engagement has been demonstrated where parents could self-select which program they engaged in (Aalborg et al., 2012; Byrnes et al., 2012; Miller et al., 2011). Importantly, these researchers demonstrated that different types of parents selected different programs, suggesting one size does not fit all. Parents who took part in a face-to-face group program typically rated their children’s externalising behaviours as more severe and perhaps felt they required more in-depth and individualised support (Miller et al., 2011). These findings suggest that allowing parents to choose from different intervention intensity levels to match their needs, may help to increase engagement. One possible solution is to provide preventive parenting programs as part of a ‘stepped care approach’. This approach could include programs with different levels of intensity as well as different delivery modalities (e.g., self-directed, group, and individual; Sanders et al., 2000). Practitioners and researchers could direct parents to the appropriate level of assistance, based on both parent preferences and an assessment of the child’s level of risk (e.g., universal, selective, or indicated prevention programs; Haggerty & Mrazek, 1994). This stepped care approach has been modelled through the multilevel system of Triple-P interventions (Sanders, 2008; Sanders et al., 2000) and has demonstrated effectiveness in certain populations (Nowak & Heinrichs, 2008).

### Strengths and limitations of this review

To our knowledge, this is the first systematic review of studies with the primary outcome of measuring and predicting parent engagement in programs that are specifically focused on the prevention of child mental health problems. Unlike other reviews that focused on reviewing literature for one specific type of parenting programs (e.g., Behavioural Parent Training; Chacko et al., 2016), this review did not place any restrictions on the type of preventive parenting program or type of mental disorder the program aimed to prevent. Additionally, we placed no restrictions on the age of the child at the time the program was delivered. Employing wider inclusion criteria allowed us to draw together the sparse literature and develop recommendations both for increasing parental engagement, and for the reporting of such research. However, even with this wide inclusion criteria, only 21 studies were identified. This limited our ability to draw firm conclusions and as such, all findings stemming from this review should be viewed as preliminary in nature.

Furthermore, some limitations of our findings should be noted. Firstly, there were not enough studies included in this review that consistently defined variables (both predictors of engagement, and stages of parental engagement), and that employed similar methods of analysis, to permit a meta-analysis to estimate effect sizes. As such, the Stouffer’s *p* analysis was adopted to estimate the reliability of associations between investigated predictors and parental engagement. Nonetheless, the Stouffer’s *p* method is unable to weight studies according to sample sizes (Darlington & Hayes, 2000). Furthermore, there has been a shift within the academic community away from reporting *p*-values as an indicator of significant results (Thomas & Pencina, 2016). This is due to the prevalent misuse of *p*-values to arbitrarily divide studies into significant and non-significant, which was not the intention of the founders of statistical inference (Sterne & Smith, 2001). Effect size measures along with confidence intervals have also been demonstrated to be more clinically relevant than a stand-alone *p*-value (Thomas & Pencina, 2016). In light of this, the quantitative results of this review should be interpreted with caution, and be considered instead as hypothesis-generating findings to guide future research.

As observed in other reviews (Chacko et al., 2016; Ingoldsby, 2010; Yap et al., 2016), study quality could not be accurately assessed due to poor reporting, particularly the selective reporting of ongoing engagement measures. No studies included in the current review could be considered to have low risk of bias across all six domains assessed, hence the results should be interpreted cautiously. All studies included in the current review were either RCT’s or experimental trials, resulting in an inability to assess if parents’ engagement differed for open access versus RCTs of preventive parenting programs. Many of the studies included in this review did not have a rigorous measure of the child’s current or previous mental health diagnoses. Given that mental health issues are common and potentially under-diagnosed in community-based samples (McManus et al., 2009; McManus et al., 2016), it is possible that included studies did not have truly preventive samples. Nonetheless, by including these community-based samples, this review may provide a more ecologically valid review of the potential predictors of parental engagement in prevention programs. Future studies employing rigorous diagnostic assessments and excluding data from participants with past or current diagnoses are required to verify whether predictors of parental engagement may differ across various populations. Finally, included studies consisted of articles written in English and published during or after 2004, therefore the current review findings may not generalise to studies published outside these dates or to literature published in other languages.

## Conclusion

This article aimed to synthesise current literature, to enable future researchers to better understand the factors that influence parental engagement in preventive parenting programs. One key finding is that despite much speculation and assumptions in the field about the predictors of parental engagement, the results of the empirical literature are mixed. The equivocal evidence base is largely due to inadequate reporting and standardisation of engagement definitions, and of the methodologies used to increase parental engagement. This includes limited measurement and analysis of how the age of the target child may affect parents’ engagement in preventive parenting programs. Such limitations need to be addressed in future research if the pervasive challenge of poor parental engagement in preventive parenting programs is to be overcome. Nonetheless, there is preliminary evidence that engagement enhancement methods which are consistent with theories such as the Health Beliefs Model and the Theory of Planned Behaviour and Reasoned Action, may increase parents’ intent to enrol and actual enrolment (e.g., personalised letters and phone calls). Furthermore, increasing parents’ perceived control (e.g., providing a choice of programs) may increase ongoing engagement in the program. Further research is required to verify the effectiveness of incorporating such methods in engaging parents in programs designed to reduce child mental health problems.

##  Supplemental Information

10.7717/peerj.4676/supp-1 Supplemental Information 1 PRISMA checklist

10.7717/peerj.4676/supp-2 Supplemental Information 2 Search strategy, inclusion and exclusion criteria, decision rules, *p*-value selection rules

10.7717/peerj.4676/supp-3 Supplemental Information 3 Descriptive summary of included studies

10.7717/peerj.4676/supp-4 Supplemental Information 4 Descriptive summary of parenting programs included in review

10.7717/peerj.4676/supp-5 Supplemental Information 5 Descriptive summary of included studies by themes as related to the three stages of engagement

10.7717/peerj.4676/supp-6 Supplemental Information 6 Descriptive summary of risk of bias of included quantitative studies

10.7717/peerj.4676/supp-7 Supplemental Information 7 Rationale of systematic review

## Additional Information and Declarations

### Data Availability

The following information was supplied regarding data availability: The research in this article did not generate any data or codes that are not included in the supplementary materials. This article is a systematic review of existing articles.
