# Supplementary material for: Parental engagement in preventive parenting programs for child mental health: a systematic review of predictors and strategies to increase engagement
Source: PeerJ. 2018 Apr 27;6:e4676. doi: 10.7717/peerj.4676 (PMC5926551; doi:10.7717/peerj.4676)
Supplement: Supplemental Information 1 [file peerj-06-4676-s001.doc]

| **Section/topic** | **#** | **Checklist item** | **Reported on page #** |
| --- | --- | --- | --- |
| **TITLE** | | |  |
| Title | 1 | Identify the report as a systematic review, meta-analysis, or both. *Title identifies article as a systematic review of predictors and strategies to increase engagement* | p.1 |
| **ABSTRACT** | | |  |
| Structured summary | 2 | Provide a structured summary including, as applicable: background; objectives; data sources; study eligibility criteria, participants, and interventions; study appraisal and synthesis methods; results; limitations; conclusions and implications of key findings; systematic review registration number. *Please see abstract for structured summary* | p.2 |
| **INTRODUCTION** | | |  |
| Rationale | 3 | Describe the rationale for the review in the context of what is already known. *Introduction includes details about child mental health problems and their effect on children and society and how prevention is one possible solution. Preventive parenting programs have shown promise in preventing both internalising disorders (Yap et al., 2016) and behaviour problems, as well as increase other child competencies (Sandler et al., 2011; 2015). Despite the potential benefits of preventive parenting programs, many studies examining the effectiveness of such programs have reported difficulties in engaging parents (Ingoldsby, 2010; Gross et al, 2001; Orrel-Valente, Piderhughes et al., 1999; Panter-Brick et al., 2014). There are two recent reviews completed by Ingoldsby (2010) and Chacko et al. (2016) that begin to look at the subject of engagement in parenting programs, Importantly Ingoldsby (2010) reviewed ongoing engagement and retention of families attending both intervention and indicated prevention programs designed to improve child mental health (child age range not specified). Notably this review did not include “…studies that focused on family enrolment unless the investigators also clearly hypothesised that the intervention would improve ongoing engagement or retention” (p.631). Universal and selective prevention programs were also not included in this review. While Chacko and colleagues (2016) reviewed and discussed predictors for parent engagement, within the domains of recruitment attrition, attendance (ongoing engagement) and treatment adherence (quality). This review included studies researching one type of parenting programs for parents of children aged 2-12 years (Behavioural Parent Training, BPT). This review also included a large number of studies because it examined multiple primary outcomes (including SES and child age), but did not focus on the factors influencing initial engagement. Another limitation of this review is that it did not review other types of evidence-based programs that have also reported engagement challenges (Heinrichs et al., 2005; Spoth & Redmond, 2000). Therefore, the current review looks to extend of these studies by including programs specifically focused on the prevention of child mental health problems across childhood and adolescence (0-18 years). Specifically, this review focuses on the initial engagement and ongoing engagement components of parental engagement.* | p.5-7 |
| Objectives | 4 | Provide an explicit statement of questions being addressed with reference to participants, interventions, comparisons, outcomes, and study design (PICOS). *Aims and Questions as follows: Specifically, this review aimed to: 1) investigate the predictors of parental engagement in preventive parenting programs, across the initial engagement (intent to enrol and enrolment) and ongoing engagement (attendance) components. Of particular interest, we aim to examine whether parental engagement differs depending on the age of the child at the time of parent participation; and 2) explore if any strategies used by researchers to increase parental engagement have been successful.* | p.7 |
| **METHODS** | | |  |
| Protocol and registration | 5 | Indicate if a review protocol exists, if and where it can be accessed (e.g., Web address), and, if available, provide registration information including registration number. ***Systematic review registration:*** *PROSPERO CRD42014013664* | p.2 |
| Eligibility criteria | 6 | Specify study characteristics (e.g., PICOS, length of follow-up) and report characteristics (e.g., years considered, language, publication status) used as criteria for eligibility, giving rationale*. This review was conducted following the Cochrane Collaboration guidelines (Higgins & Green, 2009). Controlled trials (randomised and non-randomised), cross-sectional, case-control, and longitudinal studies were considered for inclusion. Articles were required to include participants who were defined as parents or primary caregivers of children aged 0-18 years. This wide child age range was used to maximise variance and the number of eligible studies, to explore whether child age is associated with parental engagement. Parents had to be 18 years or older. Interventions were those designed to prevent the development of mental health problems in children, where parents took part in at least 50% of the intervention. Interventions could be either group or individual programs and delivered face-to-face or via phone, mail or internet. To be included in this review, the articles were required to contain: 1) analysis of the predictors of parent engagement, or 2) an evaluation of the effects of an engagement strategy on parents’ subsequent engagement in the parenting program. For more information see S1* | p.7-9, S1 |
| Information sources | 7 | Describe all information sources (e.g., databases with dates of coverage, contact with study authors to identify additional studies) in the search and date last searched. *The following electronic databases were searched: Cochrane Library, Informit online, Ovid MEDLINE, ProQuest, PsycINFO, PubMed and Scopus. The search was limited to studies written in English and articles published between 2004-2014. This publication date range was chosen to increase the likelihood that findings from this review will be more recent and relevant to current and future parenting programs. The initial search was conducted on the 12^th^ of January 2015. To ensure that the latest data was included in the review, an update search was conducted on the 21^st^ of July 2016 which included articles published between January 2015-July 2016. A set of search terms were developed after consultation with a Post-Graduate Librarian Liaison. All terms within each concept were combined with OR and each concept was combined with AND. Search terms were truncated and explored to ensure all associated terms were included.* | p.8 |
| Search | 8 | Present full electronic search strategy for at least one database, including any limits used, such that it could be repeated. *A full description of the search strategy for the MEDLINE database is listed below (this was adapted and modified as necessary for other databases):*   1. *Participat* OR engag* OR involve* OR uptake OR retention OR attrition OR recruit* OR enrol* OR dropout OR non-compliance OR adherence OR screen* OR evaluat* OR effect OR barrier* OR treat** 2. *Parent* OR guardian* OR caregiver* OR carer OR mother OR father OR dad Or mum OR mom OR famil** 3. *Program OR train* OR group* OR intervention OR behav* management* 4. *Prevent** 5. *Child* OR adolesce* OR teen* OR juvenile OR young person OR youth* 6. *Mental health OR internal* OR external* OR conduct OR anxiety OR depress* OR emotion** 7. *1 AND 2 AND 3 AND 4 AND 5 AND 6* | p.8 |
| Study selection | 9 | State the process for selecting studies (i.e., screening, eligibility, included in systematic review, and, if applicable, included in the meta-analysis). *Titles and abstracts of identified studies were reviewed to determine if they met inclusion criteria (see S1 for more detail about the inclusion/exclusion criteria* | p.8-9,  Figure 1 |
| Data collection process | 10 | Describe method of data extraction from reports (e.g., piloted forms, independently, in duplicate) and any processes for obtaining and confirming data from investigators. *). Full texts of articles that appeared to meet inclusion criteria were assessed by the first author (SF). Thirty-five percent of these titles and abstracts were independently assessed by a second author (BS) to check for inter-rater reliability of the inclusion criteria. Inter-rater reliability of inclusion criteria was 99.2%, with one additional article being included in the review. All reasons for exclusion of potentially relevant studies are documented in the PRISMA diagram (Figure 1). Importantly, studies were excluded if 1) engagement and/or retention were not primary outcomes of the study (n= 155) and/or 2) the studies had insufficient information to determine their definition of engagement (n= 32). Data extraction from all included studies was conducted by two authors (SF and BS) using a standardised, pilot-tested extraction sheet. Disagreements were resolved through discussion, with involvement of other authors when necessary.* | p.8-9, S1 |
| Data items | 11 | List and define all variables for which data were sought (e.g., PICOS, funding sources) and any assumptions and simplifications made. *Engagement factors were identified as factors that could influence a parent’s engagement in preventive parenting programs. Themes were specified when two or more of the included studies examined the same engagement variable. The themes identified included: parent age, gender of parent, parent education status, parent employment status, parent race/ethnicity, parental mental health status, child age, child gender, child mental health symptoms, family structure and one- or two- parent households.*  *P-values were also sort from all studies, these values have been recorded in supplementary materials.*  *Assumptions:*  *Two hierarchies were created because for intent to enrol Dependent Variables (DV), the informant is the parent in all cases; and for all other stages of engagement (enrolment and attendance), the informant is the researchers in all cases. As many of the Independent Variables (IV) being measured are self-reported demographics (i.e. age in years, number of hours spent in paid employment), it was assumed that these factors would be more valid and reliable if reported by parents. Additionally, most of the included studies relied on parent-reported data as the IV. Therefore, parent-reported IV’s were prioritised over teacher- and researcher-reported IV’s. A total of three potential reporter combinations for intent to enrol and other stages of engagement were subsequently formed. For intent to enrol, the combinations in descending order of preference are: 1) DV reported by parent – IV reported by parent, 2) DV reported by parent – IV reported by teacher, and 3) DV reported by parent – IV reported by researcher. For other stages of engagement, the combinations in descending order of preference are: 1) DV reported by researcher – IV reported by parent, 2) DV reported by researcher – IV reported by teacher and 3) DV reported by researcher – IV reported by researcher* | P.9-10 ,S1-4 |
| Risk of bias in individual studies | 12 | Describe methods used for assessing risk of bias of individual studies (including specification of whether this was done at the study or outcome level), and how this information is to be used in any data synthesis.  *Critical appraisal of quantitative studies was based on the Cochrane Risk of Bias Tool (Higgins & Green, 2009), which involved assessing for adequate sequence generation, allocation concealment, blinding of assessors to treatment condition, the inclusion of intention to treat analysis and assessment of potential confounders. Risk of bias for all included studies was assessed by two authors (SF and BS) using a standardised, pilot-tested extraction sheet. Disagreements were resolved through discussion.*  *Due to many studies obtaining several unclear bias ratings, the quality of the included studies remains inconclusive. As demonstrated in Table 2, the maximum number of low bias ratings for any individual study was three (Bjorknes, Jakobsen & Naerde, 2011; Bjorknes & Manger, 2013; Hellenthal, 2009). See Table 2 for a summary of results from the risk of bias assessment (for more details, see Table S6).* | p.11 |
| Summary measures | 13 | State the principal summary measures (e.g., risk ratio, difference in means).  *To partly compensate for this limitation, the Stouffer’s method (Stouffer et al., 1949) of combining p-values was identified as an appropriate method for synthesizing the findings of many of the included studies. Stouffer’s p tests are a method of combining significance levels found in multiple studies, rather than assessing effect magnitude* | p.9-10 |
| Synthesis of results | 14 | Describe the methods of handling data and combining results of studies, if done, including measures of consistency (e.g., I^2^) for each meta-analysis.  *Data extraction from all included studies was conducted by two authors (SF and BS) using a standardised, pilot-tested extraction sheet. Disagreements were resolved through discussion, with involvement of other authors when necessary. Engagement factors were identified as factors that could influence a parent’s engagement in preventive parenting programs. Themes were specified when two or more of the included studies examined the same engagement variable. A meta-analysis was not possible due to substantial differences in interventions, settings, predictor variables, and analytic methods. To partly compensate for this limitation, the Stouffer’s method (Stouffer et al., 1949) of combining p-values was identified as an appropriate method for synthesizing the findings of many of the included studies. Stouffer’s p tests are a method of combining significance levels found in multiple studies, rather than assessing effect magnitude*. | p.13-15 for data see S2-4 |

Page 1 of 2

| **Section/topic** | **#** | **Checklist item** | **Reported on page #** |
| --- | --- | --- | --- |
| Risk of bias across studies | 15 | Specify any assessment of risk of bias that may affect the cumulative evidence (e.g., publication bias, selective reporting within studies). *Table 2 and S6 have a complete breakdown of all risk of assessment.* | p.11-12 |
| Additional analyses | 16 | Describe methods of additional analyses (e.g., sensitivity or subgroup analyses, meta-regression), if done, indicating which were pre-specified. *Stouffer’s P on analysis completed* | p.13-15 |
| **RESULTS** | | |  |
| Study selection | 17 | Give numbers of studies screened, assessed for eligibility, and included in the review, with reasons for exclusions at each stage, ideally with a flow diagram. *See figure 1 for details. From more than 13,000 studies identified in the initial searches of published literature, 358 were full-text screened and 333 articles were excluded (see Figure 1 for reasons). The remaining 23 articles were included, which involved 21 separate studies*. | p.11,  Figure 1 |
| Study characteristics | 18 | For each study, present characteristics for which data were extracted (e.g., study size, PICOS, follow-up period) and provide the citations. *See table 3 and S2, 3 ,4 for complete details. In the text drtails included: Of the 21 studies included, most involved universal prevention programs, and were conducted in the USA (see Table 3). The most common mental health problem targeted was externalising disorders (n=13, i.e. conduct disorder). 19 studies were randomised controlled trials (RCT), while two were non-randomised experimental trials. Although the inclusion criteria allowed for a broader range of study designs only experimental trials met the additional inclusion criteria i.e. studies assessing parent engagement. The included studies can be categorised into two not-mutually-exclusive groups: 1) studies measuring predictors of engagement (n=17), and 2) studies that evaluated engagement methodologies (n=8). Some studies had dual aims, i.e. evaluation of an engagement methodology and measurement of predictors.* | p. 11-13, table 3 and S2, 3, 4 |
| Risk of bias within studies | 19 | Present data on risk of bias of each study and, if available, any outcome level assessment (see item 12).  *Due to many studies obtaining several unclear bias ratings, the quality of the included studies remains inconclusive. As demonstrated in Table 2, the maximum number of low bias ratings for any individual study was three (Bjorknes, Jakobsen & Naerde, 2011; Bjorknes & Manger, 2013; Hellenthal, 2009). See Table 2 for a summary of results from the risk of bias assessment (for more details, see Table S6).* | P 11, table 2, S6 |
| Results of individual studies | 20 | For all outcomes considered (benefits or harms), present, for each study: (a) simple summary data for each intervention group (b) effect estimates and confidence intervals, ideally with a forest plot. | n/a |
| Synthesis of results | 21 | Present results of each meta-analysis done, including confidence intervals and measures of consistency. | n/a |
| Risk of bias across studies | 22 | Present results of any assessment of risk of bias across studies (see Item 15).  *Due to many studies obtaining several unclear bias ratings, the quality of the included studies remains inconclusive. As demonstrated in Table 2, the maximum number of low bias ratings for any individual study was three (Bjorknes, Jakobsen & Naerde, 2011; Bjorknes & Manger, 2013; Hellenthal, 2009). See Table 2 for a summary of results from the risk of bias assessment (for more details, see Table S6).* | P 11, table 2, S6 |
| Additional analysis | 23 | Give results of additional analyses, if done (e.g., sensitivity or subgroup analyses, meta-regression [see Item 16]). | n/a |
| **DISCUSSION** | | |  |
| Summary of evidence | 24 | Summarize the main findings including the strength of evidence for each main outcome; consider their relevance to key groups (e.g., healthcare providers, users, and policy makers).  *The key findings that will be discussed include:*   - *Limited consistent evidence for predictors of parental engagement in preventive parenting programs.* - *Limited evidence to assess the association between the age of the target child and parental engagement in preventive parenting programs.* - *Preliminary evidence that enhanced recruitment methods that are consistent with health behaviour theories (i.e. Health Belief Model and Theory of Planned Behaviour) may increase parents’ initial levels of engagement (intent to enrol and enrolment) in programs.* - *Further research required to more accurately define and assess engagement, as well as the methodology used to increase parental engagement.* | p. 11-16 |
| Limitations | 25 | Discuss limitations at study and outcome level (e.g., risk of bias), and at review-level (e.g., incomplete retrieval of identified research, reporting bias).  *However, limitations of our findings should be noted. Firstly, there were not enough studies included in this review that consistently defined variables (both predictors of engagement, and stages of parental engagement), and that employed similar methods of analysis, to permit a meta-analysis to estimate effect sizes. As such, the Stouffer’s p analysis was adopted to estimate the reliability of associations between investigated predictors and parental engagement. Nonetheless, the Stouffer’s p method is unable to weight studies according to sample sizes (Darlington & Hayes, 2000). Furthermore, there has been a shift within the academic community away from reporting p-values as a demonstration of significant results (Thomas & Pencina, 2016). This is due to the prevalent misuse of p-values to arbitrarily divide studies into significant and non-significant, which was not the intention of the founders of statistical inference (Sterne & Smith, 2001). Effect size measures along with confidence intervals have also been demonstrated to be more clinically relevant than a stand-alone p-value (Thomas & Pencina, 2016). These limitations indicate that the quantitative results of this review should be interpreted with caution, and be considered as hypothesis-generating findings to guide future research.* | p. 21-22 |
| Conclusions | 26 | Provide a general interpretation of the results in the context of other evidence, and implications for future research.  *This review found limited consistent evidence of factors associated with parental engagement in preventive parenting programs. Interestingly, individual characteristics such as gender and indicators of SEP (family structure, one- or two-parent households and parent education) appeared to have limited to no support in predicting parental engagement across all stages of engagement.*  *Only one predictor, child mental health symptoms, was found to have reliable evidence in increasing enrolment. Parents with children who had increased child mental health symptoms were more likely to enrol. This association was not evident for attendance, suggesting that increased child mental health symptoms may lead a parent to enrol, but once the program has started they may drop out.*  *Despite the difficulties in comparing the different engagement enhancement methods used by researchers, the current review found preliminary support for a range of methods modelled on the Health Belief Model and the Theory of Planned Behaviour and Reasoned Action, which could increase parents’ intent to enrol and enrolment.*  *Recommendations for future research: clearer definitions and reporting, development of engagement strategies based on health behaviour theories and adaptation of programs based on parent need* | p. 16-23 |
| **FUNDING** | | |  |
| Funding | 27 | Describe sources of funding for the systematic review and other support (e.g., supply of data); role of funders for the systematic review.  *This research did not receive any specific grant from funding agencies in the public, commercial, or not-for-profit sectors. Samantha Finan is supported by Australian Government Research Training Program (RTP) Scholarship for her candidature in the Doctor of Psychology in Clinical Psychology at Monash University. Dr Naomi Priest is supported by the ANU Centre for Social Research and Methods, ANU; Dr Marie Yap is supported by a Career Development Fellowship (1061744) from the National Health and Medical Research Council.* | p.23-24 |

*From:*  Moher D, Liberati A, Tetzlaff J, Altman DG, The PRISMA Group (2009). Preferred Reporting Items for Systematic Reviews and Meta-Analyses: The PRISMA Statement. PLoS Med 6(7): e1000097. doi:10.1371/journal.pmed1000097

For more information, visit: **www.prisma-statement.org**.

Page 2 of 2
