# Supplementary material for: Parental engagement in preventive parenting programs for child mental health: a systematic review of predictors and strategies to increase engagement
Source: PeerJ. 2018 Apr 27;6:e4676. doi: 10.7717/peerj.4676 (PMC5926551; doi:10.7717/peerj.4676)
Supplement: Supplemental Information 2 [file peerj-06-4676-s002.docx]

# Online Supplement 1: **Search Strategy, Inclusion and Exclusion Criteria, Decision Rules, *p*-value Selection Rules**

# Search strategy

# All terms within each concept were combined with OR and each concept was combined with AND. Search terms were truncated and explored to ensure all associated terms were included. A full description of the search strategy for the MEDLINE database is listed below (this was adapted and modified as necessary for other databases):

# Participat* OR engag* OR involve* OR uptake OR retention OR attrition OR recruit* OR enrol* OR dropout OR non-compliance OR adherence OR screen* OR evaluat* OR effect OR barrier* OR treat*

# Parent* OR guardian* OR caregiver* OR carer OR mother OR father OR dad OR mum OR mom OR famil*

# Program OR train* OR group* OR intervention OR behav* management

# Prevent*

# Child* OR adolesce* OR teen* OR juvenile OR young person OR youth

# Mental health OR internal* OR external* OR conduct OR anxiety OR depress* OR emotion*

# 1 AND 2 AND 3 AND 4 AND 5 AND 6

# Study characteristics

Include if:

1. Longitudinal
2. Cross-sectional
3. Case-control
4. Cohort study
5. Peer-reviewed full-text journal article
6. Dissertation

Exclude if:

1. Therapy/treatment intervention (*note: all prevention interventions are eligible for inclusion)*
2. Review or meta-analysis
3. Qualitative
4. Discussion paper
5. Language other than English

# Main aim of article

Include if:

1. Recruitment strategies

Exclude if:

1. Prevention of mental health

**Age of parent**

Include if:

1. Parent >18 years

Exclude if:

1. Parent <18 years

# Age of child

Include if:

1. Child <18 years (as described by the World Health Organisation (WHO), a child is defined as anyone under the age of 18 years)

Exclude if:

1. Pre-natal classes
2. Child >18 years

# Dependent variable

Include if:

1. Includes description of recruitment method
2. Reports on number of parents engaging at different stages of engagement (intent, enrolment, ongoing engagement)

Exclude if:

1. Lacks adequate specificity of recruitment methods

# Independent variables

Include if:

1. Variables are potentially factors in parents’ degree of engagement

Exclude if:

1. Lacks adequate specificity (e.g., composite measure, measure of general psychopathology)
2. Study which compares diagnostic groups but does not include a normal (non-clinical) control group
3. Study is evaluating therapy or treatment for children with existing depression or anxiety disorders

# Decision hierarchies

Due to the large variation of reporters of predictor and outcome variables in the included papers, the following hierarchies were developed as an index of the quality of the evidence based on the informants of the variables of interest. Two hierarchies were created as the dependent variable (DV) informant for intent to enrol will always be the parent and for all other stages of engagement (enrolment and ongoing engagement) the informant will always be the researchers.

As many of the independent variables (IV) being measured are self-reported demographics (i.e. age in years, number of hours spent in paid employment), it was assumed that these factors would be more valid and reliable if reported by parents. Additionally, most of the data reported in the studies includes parent-reported data as the IV. Therefore, parent-reported IV’s were prioritised over teacher- and researcher-reported IV’s.

## Hierarchy of reporter combinations for intent to enrol

Based on the above hierarchies, there are 3 IV-DV reporter combinations, which were ordered in the following hierarchy:

1. DV reported by parent – IV reported by parent
2. DV reported by parent – IV reported by teacher
3. DV reported by parent – IV reported by researcher

## Hierarchy of reporter combinations for enrolment and ongoing engagement

Based on the above hierarchies, there are 3 IV-DV reporter combinations, which were ordered in the following hierarchy:

1. DV reported by researcher – IV reported by parent
2. DV reported by researcher – IV reported by teacher
3. DV reported by researcher – IV reported by researcher

## Hierarchy for extracting parenting variable (IV)

1. Combined paternal and maternal parenting
2. Maternal parenting only
3. Paternal parenting only

Mother-report of parenting was extracted over father-report of parenting; these decisions were made as a reflection of most studies in parenting program research, which have recruited and relied primarily on mothers.

# *P*-value selection rules

1. If both bivariate correlations and regression coefficients (controlling for covariates etc.) are reported, select the former
2. If non-significant *p*-values are not quoted, we allocate the association a conservative one-tailed *p*-value of 0.5
3. If significant *p*-values are not quoted, we allocate the association the minimum *p*-value required to indicate significance as stated by the study (i.e. if *p*<.05 then *p*=.05 allocated, or if *p*<.01 then *p*=.01 allocated)
4. Unless stated otherwise in the study we will assume two-tailed significance. This is due to the exploratory nature of many of the studies being reviewed
5. If a study reports data for overall mental health symptom measures, as well as separate symptom subscales, overall measures will be selected over individual subscales, as they tend to have better psychometric properties
6. If a study reports data at pre-test screening and then at subsequent time points, the pre-test screening will be selected over additional time points; this will lead to a truer measure of the factors pre- intent, enrolment or attendance to the program, that could have an effect on the stages of engagement
