# Supplementary material for: Parental engagement in preventive parenting programs for child mental health: a systematic review of predictors and strategies to increase engagement
Source: PeerJ. 2018 Apr 27;6:e4676. doi: 10.7717/peerj.4676 (PMC5926551; doi:10.7717/peerj.4676)
Supplement: Supplemental Information 3 [file peerj-06-4676-s003.docx]

### Online Supplement 2: Descriptive Summary of Included Studies

## Table 2a

## *Characteristics of studies testing recruitment methodologies*

| Study | Country | Parenting program | Recruitment methodology | Inclusion and exclusion criteria | Total sample enrolled (*n*=) | Measures of engagement | Diagnostic tools |
| --- | --- | --- | --- | --- | --- | --- | --- |
| Aalborg (2012) and Miller (2011) | USA | Family Matters (FM) &  Strengthening Families Program: For Parents and Youth 10-14 (SFP) | Mail out or generic advertisement | Parents were eligible for study if: 1) they had an adolescent aged 11-12 years, 2) they spoke functional English, and 3) their adolescent was not previously or currently engaged in substance use treatment | 614 | **Intent:** not defined **Enrolment:** parents who showed initial interest in study and signed consent form  **Ongoing engagement:** number of sessions attended or number of books completed | SFP; number of sessions attended  FM; time taken to complete booklets, number of booklets completed, time spent on phone with health professionals |
| Bjorknes (2011) and Bjorknes (2013) | Norway | Parent Management Training - The Oregon Model (PMTO) | Personal invitations | Parents were eligible for the study if they: 1) had a child aged 3-9 years who was at risk of developing conduct problems, and 2) were mothers identified as refugees from Somali and Pakistan and were currently living in Norway | 96 | **Intent:** not defined **Enrolment:** parents who were eligible and signed consent form  **Ongoing engagement:** 1) number that started program or waitlist, and 2) number that attended 10+ sessions | **Labour intensiveness:** days spent per strategy by recruitment team **Sociodemographic variables:** mother's ethnic origin, mother's age, child's age and gender, number of children in family, number of years mother had lived in Norway, education, employment status and if family received public financial support, mother's language skills **Parenting practices:** PPI; self-report questionnaire tapping parents’ discipline of young children **Child behaviour factors:** Eyberg Child Behaviour Inventory (ECBI) and Teacher Report Form (TRF), Social Skills Rating System (SSRS) |
| Byrnes (2012) ^a^ | USA | Family Matters (FM) &  Strengthening Families Program: For Parents and Youth 10-14 (SFP) | Mail out or generic advertisement | Parents were eligible for the study if: 1) they had an adolescent aged 11-12 years, 2) spoke functional English, and 3) adolescent was not previously or currently engaged in substance use treatment | 214 | **Intent:** whether parent agreed to participate in study as indicated by scheduling a baseline interview  **Enrolment:** whether family enrolled in study as indicated by signing of consent form and completing face-to-face baseline interview  **Ongoing engagement**: not measured/defined | **Neighbourhood disorganisation:** gathered from 2000 census data on population and housing **Neighbourhood SES:** gathered from 2000 census data including rates of overall unemployment, persons below the poverty line, households receiving public assistance, high school dropouts and female-headed households  **Residential instability:** proportion of residents who have moved in past five years  **Sociodemographic variables:** ethnicity, parent’s level of education, parent’s age and youth's gender |
|  |  |  |  |  |  |  |  |
| Calam (2008) ^a^ | UK | Driving Mum and Dad Mad | Mail out or generic advertisement | Nil reported | 723 | **Intent:** not defined **Enrolment:** not defined  **Ongoing engagement:** defined as dichotomous variable of watching all or less than all episodes, also measured average number of episodes viewed by parents | **Sociodemographic variables:** Family Background Questionnaire (FBQ)  **Child behaviour factors**: Eyberg Child Behaviour Inventory (ECBI)  **Parenting practices:** Parenting Scale (PS), Parental Anger Inventory (PAI), PPC Problem scale, Parenting Tasks Checklist  **Parent behaviour factors:** Depression Anxiety Stress Scale (DASS), Relationship Quality Index (RQI) |
| Carpentier (2007)^a^ | USA | Bridges to High School | Mail out plus phone call | Parents were eligible for the study if: 1) child was in 7th grade, under 15 years of age and enrolled in one of five recruitment schools, 2) one biological parent was of Mexican descent, and 3) spoke either English or Spanish | 596 (initial enrolment analyses)  353 (program enrolment into intervention condition) | **Intent:** not defined **Enrolment:** enrolment in program was indicated as completion of initial home visit session **Ongoing engagement:** family attendance was measured by number of sessions where at least one family representative was present | **Sociodemographic variables:** family language preference, number of hours worked per week, family aggregate income, number of children in home, single parent status, family education level (assessed through combining individual caregivers’ education levels)  **Child behaviour variables:** Child Behaviour Checklist (CBCL), Grade Point Average (GPA)  **Variables assessed for both parent and adolescent:**  Acculturation Rating Scale for Mexican Americans, familism  **Group environment:** Moos Group Environment Scale |
| Eisner (2011) ^a^ | Switzerland | Triple P | Practitioner-led dissemination. Mail out plus researchers spending time at centres | Nil reported | 257 | **Intent:** not defined  **Enrolment:** agreement to take part in study, signed informed consent  **Ongoing engagement:** parents who attended at least one session (participation), parents who attended all 4 sessions (completion) | **Sociodemographic variables:** single parent, dual-earner family, number of children, language, International Socio-Economic Index of Occupational Status (ISEI), neighbourhood networks  **Parenting practices:**  Alabama Parenting Questionnaire  **Parent behaviour variables:** previous service utilisation, course climate (assessed by program providers)  **Child behaviour variables:**  Externalising Problem Behaviour subscale of the Social Behaviour Questionnaire |
| Heinrichs (2006) | Germany | Triple P | Mail out plus researchers spending time at centres | Parents were required to: 1) have a basic understanding of the German language, and 2) have a child aged 2.6-6 years attending one of the kindergartens advertising the trial | 197 | **Intent:** initial enrollers were parents who listed contact details and/or booked in the initial session time  **Enrolment:** included the final recruited sample  **Ongoing engagement:** assessed in hours of intervention received | **Sociodemographic variables:** parent and child age, parent education status, parent occupational status, marital status, formal relationship to child  **Child behaviour factors:** Child Behaviour Checklist 1^1/2^-5 (CBCL) |
| Helfenbaum- Kun (2007) | USA | Webster-Stratton’s Incredible Years program | Mail out plus researchers spending time at centres | Fathers were eligible for the study if: 1) they had a child between 3-5 years enrolled at a head start centre, 2) spoke English or Spanish, and 3) parents were married and resided together, or unmarried and resided together for at least the past year | 39 | **Intent:** not defined **Enrolment:** agreeing at the recruitment sessions to take part in the study **Ongoing engagement:** number of sessions attended | **Child behaviour variables:** Eyberg Child Behaviour Inventory (ECBI), teacher-report of the Intensity scale of the Sutter-Eyberg Student Behaviour Inventory-Revised (SESBI-R)  **Parent behaviour variables:** Parenting Scale, Block Child Rearing Practices Report (CRPR), Dyadic Adjustment scale (DAS), Child-Care Task Checklist (CCTC), Parenting Alliance Measure (PAM) |
| Mian (2015) ^a^ | USA | Program not named: once-off anxiety prevention seminar | Pre-screeners | Parents were eligible for the study if: 1) children were receiving nutritional assistance at children’s hospital and aged 11-71 months, 2) aged at least 18 years themselves, 3) spoke English or Spanish, and (4) had a child considered high-risk according to one or more of following; elevated child anxiety symptoms, elevated parent anxiety symptoms, or child exposure to a potentially traumatic event | 101 | **Intent:** not assessed before enrolment **Enrolment:** parents who were eligible and requested to be contacted  **Ongoing engagement:** parents were asked if they planned to attend and to reply via RSVP card or phone call | **Sociodemographic variables:** risk; parent’s highest level of education, family income, English language proficiency, and parent immigrant status **Child behaviour variables:** Brief Infant-Toddler Social and Emotional Assessment (BITSEA), Life Events Checklist  **Parent behaviour variables:** parent service preferences (service format, type, topic, incentives, service characteristics), Beck Anxiety Inventory (BAI), Parent Satisfaction Survey |
|  |  |  |  |  |  |  |  |

*Notes:*

*^a^ Studies also report on predictors of engagement*

Table 2b

*Characteristics of studies measuring predictors of engagement*

| Study | Country | Parenting program | Recruitment methodology | Inclusion and exclusion criteria | Total sample enrolled (*n*=) | Measures of engagement | Diagnostic tools |
| --- | --- | --- | --- | --- | --- | --- | --- |
| Baker (2011) | USA | Webster-Stratton’s Incredible Years program | Mail out plus researchers spending time at centres | Parents needed to have a child attending a preschool classroom in the childcare centre advertising study | 106  193 agreed to participate, however current study was concerned with intervention condition subset (who gave informed consent) of 106 children. Of these 51 actually enrolled (i.e. attended at least 1 session) | **Intent:** not defined **Enrolment:** assessed as a dichotomous variable (never participated in program or participated in at least one program session)  **Ongoing engagement:** measured for parents that attended at least one session, and calculated as a percentage of sessions attended | **Sociodemographic variables**: socioeconomic status (high or low income based on childcare centre child attended), single parenthood  **Child behaviour factors:** Teacher's Report Form (TRF)  **Parent behaviour factors:** Parent Satisfaction Ratings, Brief Symptom Inventory (BSI), Parent Social Support (PSS) |
| Brody (2006) | USA | Strong African American Families (SAAF) | Mail out plus phone call | Parents needed to: 1) have an 11-year-old child in a school participating in the research, and 2) be of African American ethnicity | 322  150 families in the control counties and 172 families in the intervention counties. Since the present study addresses family participation in the intervention, the analyses include only those participants randomly assigned to the prevention group | **Intent:** not defined **Enrolment:** not defined **Ongoing engagement:** defined as the total number of sessions that each family attended (dosage) | **Sociodemographic variables:** ratio of children to adults in household, perceived economic stress (Money for Necessities subscale of Family Resource Scale), overall family risk score  **Child behaviour factors:** youth unconventionality  **Parent behaviour factors:** Centre for Epidemiologic Studies Depression Scale (CES-D), level of involvement in religious activities, communicative parenting - 4 indicators including: 1) involved-vigilant parenting, 2) adaptive racial socialisation (Racial Socialisation Scale), 3) communication about sex (Parental Communication About Sex Scale), and 4) clear communication of expectations about alcohol use  **Variables assessed for both parent and adolescent:**  Interaction Behaviour Questionnaire (IBQ) |
| Fleming (2015) | USA | Common Sense Parenting (CSP) | Mail out or generic advertisement. Where possible, researchers spent time at evening school events | Parents were eligible for the study if: 1) child was in 8th grade attending a school involved in the study, and 2) parents spoke English | 321 enrolled  213 in sample for analyses pertaining to predictors of enrolment; 157 for attendance/retention analyses | **Intent:** not defined **Enrolment:** attended at least one session **Ongoing engagement:** percentage of possible sessions attended (retention) | **Sociodemographic variables**: race, ethnicity, whether parent lived with a spouse or significant other, parents’ and children’s gender and age, SES - measured using household income and parent education  **Child behaviour factors**: self-reported academic performance, Strengths and Difficulties Questionnaire (SDQ) for internalising behaviours **Parent behaviour factors:** Alabama Parenting Questionnaire (APQ), parent-child affective quality |
| Garvey (2006) | USA | The Chicago Parent Program (CPP) | Mail out plus researchers spending time at centres | Parents eligible for study if they were: 1) parent or legal guardian of a 2-4 year-old child enrolled in a participating day care centre, and 2) able to speak English | 292 | **Intent:** not defined **Enrolment:** percentage of parents in target population who consented and completed baseline **Ongoing engagement:** operationalised as dose and engagement  1) dose; percent of intervention sessions attended  2) engagement; degree to which parents actively participated in sessions | **Sociodemographic variables:** parent age, parent education level, parity, employment status, economic disadvantage, marital status, child sex **Child behaviour factors:** Eyberg Child Behaviour Inventory (ECBI) parent-report and teacher-report, Caregiver-Teacher Report Form (CTRF)  **Parent behaviour factors**: Non-Participation Questionnaire, Toddler Care Questionnaire (TCQ), Everyday Stressor Index (ESI), Centre for Epidemiologic Studies Depression Scale (CESD), travel time to attend the intervention |
| Heinrichs (2005) | Germany | Triple P | Mail out plus researchers spending time at centres | Parents were eligible for the study if they: 1) had a child between 2.6-6.0 years of age, and 2) could speak German | 282 | **Intent:** not defined **Enrolment:** parents who completed consent and baseline assessment **Ongoing engagement:** choosing to attend program once allocated to intervention or control | **Sociodemographic variables:** teacher ratings - age of parents, family status, parent occupation, number of people in the family, estimated social status of the family, whether family was on welfare **Child behaviour factors:** Child Behaviour Checklist for ages 1.5-5 years, Kaufman Assessment Battery for Children (K-ABC)  **Parent behaviour factors:** videotaped parent-child interaction task, family non-participation survey |
| Hellenthal (2009) | USA | Barkley (1997)’s Behavioural Parent Training (BPT) program | Mail out or generic advertisement | Parents were eligible for the study if they: 1) lived in the community, and 2) had a child between 2-12 years | 72 | **Intent:** not defined **Enrolment:** agreed via telephone to take part in the group **Ongoing engagement:** attending 4 or more sessions | **Sociodemographic variables**: age, income, education and cultural factors, including race  **Child behaviour factors:** Disruptive Behaviour Stress Inventory (DBSI), Ohio Scales  **Parent behaviour factors:** Social Provisions Scale (SPS), Alabama Parenting Questionnaire (APQ), Family Environment Scale – Form R, Parent Sense of Competence Scale (PSOC), Therapy Attitude Inventory (TAI) |
| Mauricio (2014) | USA | Bridges to High School | Mail out or generic advertisement | To be eligible parents needed to: 1) have a child between the ages of 11-14 years, and 2) speak English or Spanish | 292  542 families in the RCT, 353 were assigned to treatment. 292 of 353 attended 1 or more sessions | **Intent:** measured after first home interview, single item on 5-point Likert scale  **Enrolment:** not defined  **Ongoing engagement:** attendance status recorded at each session | **Sociodemographic variables:** family income, comprised of wages, child support, state and federal assistance; number of biological children; self-reported education level  **Child behaviour factors:** Externalising subscale of Child Behaviour Checklist (CBCL), GPA average  **Parent behaviour factors:**  Critical Events subscale of the Barriers to Treatment Participation Scale, Centre for Epidemiologic Studies Depression Scale, Acculturation Rating Scale for Mexican-Americans-II, Mexican-American Cultural Values Scale, Moos Group Environment scale  *Parent perceptions of participation benefits;* Multicultural Inventory of Parenting Self-Efficacy, Small and Kerns' Parental Monitoring scale, Extrinsic Motivation subscale of the Parent Motivational Practices Scale |
| Nordstrom (2008) | USA | Parenting our Children to Excellence (PACE) | Mail out plus researchers spending time at centres | Day care centres needed to serve: 1) a minimum of 35 families with children between the ages of 3-6 years at the time of recruitment, and 2) an economically and ethnically diverse population | 114  347 completed telephone survey; 216 stated intent to enrol; 114 actually enrolled | **Intent:** parents were asked; "Do you intend to enrol in the parenting program that is now offered or will be offered soon at your child's preschool or day-care?" and responses were rated on a 4-point Likert scale  **Enrolment:** parents were considered enrolled when they returned the registration form or contacted the day-care centre to register  **Ongoing engagement:** parent attendance at each session was recorded on attendance logs, and summed across sessions to produce a composite score (ranging from 0-8) | **Sociodemographic variables:** child’s age and gender, parent’s age, parent ethnicity, employment status, highest level of education, marital status, yearly income  **Child behaviour factors:** Disruptive Behaviour Disorders Rating Scale  **Parent behaviour factors:**  Parenting Sense of Competence Scale, The Raising Young Children Scale, Parenting Possibilities Questionnaire and the Family Stories measure, Obstacles to Engagement Scale |
| Plueck (2010) | Germany | Prevention Program for Externalising Problem Behaviour (PEP) | Pre-screeners | Parents were eligible if they had a child aged 3-6 years in local preschool, who, after completing the screening task, was defined as being at risk for developing more severe externalising problems | 155  155 accepted invitation to pre-test. However only 74 consented for actual PEP program  2123 used for screening/intent to enrol analysis. 91 and 74 for enrolment and attendance analyses, respectively | **Intent:** defined as parents who submitted their screening survey and gave consent versus those who did not  **Enrolment:** defined as those who were eligible for the group program and agreed to attend  **Ongoing engagement:** number of sessions attended | **Socioeconomic variables:**  age and gender of child, parent’s language, teacher’s assessment of parent’s decision not to participate. SES was estimated as the mean of education and profession of both parents (classified as high, medium or low)  **Child behaviour factors:** Child Behaviour Checklist, parent- and teacher-rated global questions assessing child’s problems;  (1) “How much do you feel bothered/burdened by the child's behaviour?"  (2) Do you think you or the child need(s) professional help because of the burden?"  **Parent behaviour factors:** PEP-Screen |
| Reedtz (2011) | Norway | Webster-Stratton’s Incredible Years program | Mail out or generic advertisement | Parents were eligible if: 1) they had a child aged between 2-8 years, and 2) child scored below 90th Percentile on ECBI Intensity subscale | 189 | **Intent:** not defined **Enrolment:** volunteering to participate in the study and fill out pre-test survey  **Ongoing engagement**: not defined | **Sociodemographic variables:** child’s gender, age, number of children the parents had, target child’s birth order, parent’s birth year, marital status, employment status, education level  **Child behaviour factors:** Eyberg Child Behaviour Inventory (ECBI)  **Parent behaviour factors:** PSOC, PSI-Short Form, PPI adapted from the Oregon Social Learning Centre’s Discipline Questionnaire, reasons for participation and help-seeking behaviour |
| Skarstrand (2009) | Sweden | Strengthening Families Program: For Parents and Youth 10-14 (SFP) | Mail out plus researchers spending time at centres | School’s inclusion criteria: 1) having grades 6-9 at school, and 2) not having age-integrated classes  Parents’ inclusion criteria: child aged between 12-14 years | 388  388 responded, however only 200 agreed to participate in part 1, and 115 agreed to participate in both part 1+2 of program | **Intent:** not defined **Enrolment:** if parent consented to program and attended at least one session in part 1 of the program **Ongoing engagement:** taking part in at least one session in part 1 and at least one session in part 2 | **Sociodemographic variables:** gender, age, education, gender of target child, living with target child, working full-time, born in Sweden  **Parent behaviour factors:** parent emotional warmth and responsiveness to child’s needs, rule-setting, perception of norm-breaking behaviours, knowledge of school performance, parents’ attitude towards adolescents and alcohol |
| Winslow (2009) | USA | Program for mothers who have recently divorced (unnamed) | Mail out or generic advertisement, phone calls | Parents were eligible for the study if: 1) divorce decree was granted within the previous two years, 2) the primary residential parent was female, 3) at least one child aged 9-12 years lived with the mother most of the time, 4) the mother and children living at home were not receiving mental health treatment, 5) the mother had not re-partnered, 6) custody was expected to remain stable during the trial, 7) the family lived within a 1-hour drive of the intervention delivery site, 8) the mother and target child spoke and read English, 9) the child was not receiving special education services, and 10) if child was diagnosed with ADHD, s/he was taking medications | 325  321 used for logistic regression analysis | **Intent:** not defined **Enrolment:** mother agreed to participate in parenting intervention at the recruitment visit  **Ongoing engagement:** mothers who did not attend any sessions or dropped out before program completion were considered not retained. Mothers who completed the program were considered retained | **Sociodemographic variables:** maternal race/ethnicity, months since divorce, maternal education, income-to-needs ratio: dividing the mother's report of her annual household income by the US Census Bureau's official poverty threshold for the year in which income was reported  **Child behaviour factors:** age 4-18 version of the Child behaviour Checklist (CBCL)  **Parent behaviour factors:** child-report of Parenting Behaviour Inventory (CRPBI), Parent-Adolescent Communication Scale, inconsistent discipline subscale of CRPBI, Oregon Social Learning Centre ratio of appropriate to inappropriate discipline and follow-through scales, Psychiatric Epidemiology Research Interview (PERI) Demoralisation scale, Negative Life Events Scale |
