# Supplementary material for: Parental engagement in preventive parenting programs for child mental health: a systematic review of predictors and strategies to increase engagement
Source: PeerJ. 2018 Apr 27;6:e4676. doi: 10.7717/peerj.4676 (PMC5926551; doi:10.7717/peerj.4676)
Supplement: Supplemental Information 4 [file peerj-06-4676-s004.docx]

### Online Supplement 3: Descriptive Summary of Parenting Programs Included in Review

Table 3

*Descriptive Summary of Parenting Programs Included in Review*

| Program name | Aim | Session format | Number of sessions | Child involved | Reference |
| --- | --- | --- | --- | --- | --- |
|  |  |  |  |  |  |
| Barkley (1997)’s Behavioural Parent Training (BPT) program | Prevention of conduct disorders | Group | 8 group sessions (1.5 hours per session) | No | Hellenthal (2009) |
| Bridges to High School | Increase protective factors and decrease risk factors associated with academic engagement and mental health | Group and individual | 9 group sessions (2 hours per session) and 2 home visits  9 group sessions (2 hours per session) and 2 home visits | Yes  Yes | Carpentier (2007)  Mauricio (2014) |
| Chicago Parent Program (CPP) | Demonstrate positive child behaviours and reduce negative behaviours | Group | 11 group sessions (2 hours per session) and 1 booster session two months later | No | Garvey (2006) |
| Common Sense Parenting (CSP) | Prevention of problem behaviours | Group | CSP standard format: 6 group sessions for parents (2 hours per session)  CSP plus format: 6 group sessions for parents (2 hours per session) plus two sessions that both parent and child attend | No for CSP standard; yes for CSP plus | Fleming (2015) |
| Driving Mum and Dad Mad | Teaches positive parenting and communication skills | Individual | Format 1: 6 video episodes (30 minutes per video)  Format 2: 5 video episodes (60 minutes per video) plus workbooks/web support | No | Calam (2008) |
| Family Matters (FM) | Prevent adolescent tobacco and substance use | Individual | 4 booklets and 4 follow-up phone calls with researchers | Yes | Aalborg (2012) and Miller (2011), Byrnes (2012) |
| Parent Management Training – The Oregon Model (PMTO) | Strengthen parenting practices to prevent conduct disorder | Group | 18 group sessions (2 hours per session) | No | Bjorknes (2011) and Bjorknes (2013) |
| Parenting our Children to Excellence (PACE) | Prevention of conduct disorders | Group | 8 group sessions (2 hours per session) | No | Nordstrom (2008) |
| Prevention Program for Externalising Problem Behaviour (PEP) | Prevention of externalising behaviour problems | Group | 10 group sessions (90-120 minutes per session) | No, however teachers are provided with training | Plueck (2010) |
| Program for mothers who have recently divorced (unnamed) | Prevention of child mental health problems | Group | 11 group sessions (1.75 hours each) and 2 individual sessions (1 hour each) to tailor program to individual needs | No | Winslow (2009) |
| Program not named: once-off anxiety prevention seminar | Prevention of internalising disorders | Group | 1 group session (60 minutes in length) | No | Mian (2015) |
| Strengthening Families Program: For Parents and Youth 10-14 (SFP) | Prevent adolescent tobacco and substance use | Group | 7 weekly group sessions (2 hours per session)  Swedish version; 12 group sessions (1.5-2 hours per session) | Yes  Yes | Aalborg (2012) and Miller (2011), Byrnes (2012)  Skarstrand (2009) |
| Strong African American Families (SAAF) | Prevention of substance use and engaging in early sexual activity | Group | 7 group sessions (2 hours per session) | Yes | Brody (2006) |
| Triple P | Prevention of child behaviour problems | Individual or group | Format 1: 8 individual home visits (1 hour per session)  Format 2: 4 group sessions (2 hours per session) and 4 check-in phone calls | No | Heinrichs (2006) |
|  |  |  | 4 group sessions (2 hours per session) and 4 check-in phone calls | No | Heinrichs (2005) |
|  |  |  | 4 group sessions (2-3 hours per session), video elements, a parent workbook, up to 4 20-minute phone contacts after the course | No | Eisner (2011) |
| Webster-Stratton’s Incredible Years program | Decrease risk of development of conduct disorders | Group | 8 group sessions (2 hours per session)  Note; the original 12-15 week curriculum was reduced to 8 weeks for both studies | No | Baker (2011), Helfenbaum-Kun (2007) |
|  |  |  | Short format: 6 group sessions (2 hours per session)  Regular format: 12 group sessions (2 hours per session) | No | Reedtz (2011) |
