# Supplementary material for: Parental engagement in preventive parenting programs for child mental health: a systematic review of predictors and strategies to increase engagement
Source: PeerJ. 2018 Apr 27;6:e4676. doi: 10.7717/peerj.4676 (PMC5926551; doi:10.7717/peerj.4676)
Supplement: Supplemental Information 5 [file peerj-06-4676-s005.docx]

### Online Supplement 4: Descriptive Summary of Included Studies by Themes as Related to the Three Stages of Engagement

## Table 4a

## *Descriptive summary of the predictors of intent to enrol*

|  | **Parenting program name** | ***n*=** | **Instrument** | **Predictor of engagement** | **Intent to enrol rate** | **Statistics** | **Significance** | **Study** |
| --- | --- | --- | --- | --- | --- | --- | --- | --- |
| **1. Demographic information** | | | | | | | | |
| **Total household income** | | | | | | | | |
|  | Program not named: once-off anxiety prevention seminar | 101 | Family income (converted to a 5-point scale) | Parent income (no stated direction) | No change | Stat n/r | Non-sig | Mian (2015) |
|  | Parenting our Children to Excellence (PACE) | 322 | Yearly income (converted to a 4-point scale) | Family income (no stated direction) | No change | Wald χ^2^ = .83, OR = .88,  CI = [.68, 1.15] | *p* > .05 | Nordstrom (2008) |
| **Neighbourhood socioeconomic status** | | | | | | | | |
|  | Prevention Program for Externalising Problem Behaviour (PEP) | 2123 | Social burden of district (calculated by Department of Youth and Family Welfare) | Increased social burden of district | Decreased | OR = 1.69,  CI = [1.42, 2.01] | *p* < .01 | Plueck (2010) |
|  | Family Matters (FM) & Strengthening Families Program: For Parents and Youth 10-14 (SFP) | 214 | 2000 Census of Population and Housing | Increased neighbourhood % on public assistance | No change | OR = .80 | *p* = .108 | Byrnes (2012) |
|  | Family Matters (FM) & Strengthening Families Program: For Parents and Youth 10-14 (SFP) | 214 | 2000 Census of Population and Housing | Increased neighbourhood % below poverty line | No change | OR = 1.18 | *p* = .279 | Byrnes (2012) |
| **Neighbourhood unemployment** | | | | | | | | |
|  | Family Matters (FM) & Strengthening Families Program: For Parents and Youth 10-14 (SFP) | 214 | 2000 Census of Population and Housing | Higher levels of neighbourhood unemployment | Decreased | OR = .73 | *p* = .009 | Byrnes (2012) |
| **Transient nature of neighbourhood** | | | | | | | | |
|  | Family Matters (FM) & Strengthening Families Program: For Parents and Youth 10-14 (SFP) | 214 | 2000 Census of Population and Housing | Higher neighbourhood % of families moved in last 5 years | No change | OR = .97 | *p* = .724 | Byrnes (2012) |
| **One- or two-parent households** | | | | | | | | |
|  | Family Matters (FM) & Strengthening Families Program: For Parents and Youth 10-14 (SFP) | 214 | 2000 Census of Population and Housing | Higher neighbourhood % of female headed households | No change | OR = 1.21 | *p* = .109 | Byrnes (2012) |
|  | Parenting our Children to Excellence (PACE) | 322 | Study-specific questions answered by parent | Marital status (single parent) | No change | Wald χ^2^ = .06, OR = 1.09,  CI = [.56, 2.12] | *p* > .05* | Nordstrom (2008) |
| **2. Parent factors** | | | | | | | | |
| **Parent age** | | | | | | | | |
|  | Program not named: once-off anxiety prevention seminar | 101 | Study-specific questions answered by parent | Parent age (no stated direction) | No change | Stat n/r | Non-sig* | Mian (2015) |
|  | Family Matters (FM) & Strengthening Families Program: For Parents and Youth 10-14 (SFP) | 214 | Study-specific questions answered by parent | Parent age (no stated direction) | No change | OR = .98 | *p* = .089* | Byrnes (2012) |
|  | Parenting our Children to Excellence (PACE) | 322 | Study-specific questions answered by parent | Maternal age (no stated direction) | No change | Wald χ^2^ = 1.12,  OR = .89,  CI = [.72, 1.10] | *p* > .05* | Nordstrom (2008) |
| **Parent gender** | | | | | | | | |
|  | Program not named: once-off anxiety prevention seminar | 101 | Gender of the survey respondent | Maternal respondent | No change | Stat n/r | Non-sig | Mian (2015) |
| **Parent race/ethnicity** | | | | | | | | |
|  | Parenting our Children to Excellence (PACE) | 322 | Study-specific questions answered by parent (choice of African American or European American) | Maternal ethnicity (no stated direction) | No change | Wald χ^2^ = 3.26,  OR = .55,  CI = [.29, 1.05] | *p* > .05* | Nordstrom (2008) |
|  | Program not named: once-off anxiety prevention seminar | 101 | Parents provided opportunity to list their immigration status and ethnicity on a scale and in an open-question format | Immigration status; foreign born parent | No change | Stat n/r | Non-sig* | Mian (2015) |
| **Parent language proficiency** | | | | | | | | |
|  | Program not named: once-off anxiety prevention seminar | 101 | English proficiency measured on 5-point scale from ‘not at all’ to ‘very well’ | Parent's increased proficiency in English | No change | Stat n/r | Non-sig | Mian (2015) |
| **Parent education** | | | | | | | | |
|  | Program not named: once-off anxiety prevention seminar | 101 | 9-point scale from ‘8^th^ grade or less’ to ‘professional degree’ | Parent’s highest level of education | No change | Stat n/r | Non-sig* | Mian (2015) |
|  | Family Matters (FM) & Strengthening Families Program: For Parents and Youth 10-14 (SFP) | 214 | 2000 Census of Population and Housing | Increased neighbourhood % high school dropout (parents’ generation) | No change | OR = 1.22 | *p* = .154 | Byrnes (2012) |
|  | Parenting our Children to Excellence (PACE) | 322 | Study-specific questions answered by parent (4-point scale from high school not completed to college graduate) | Maternal education (no stated direction) | No change | Wald χ^2^ = .46, OR = .88,  CI = [.62, 1.27] | *p* > .05* | Nordstrom (2008) |
| **Parent occupation** | | | | | | | | |
|  | Program not named: once-off anxiety prevention seminar | 101 | Scale from ‘unemployed’ to ‘full-time work’ | Parent working part-time versus full-time versus unemployed | Increased | 92.9%, 64.7% and 59.4% planned to attend, respectively | Fischer's exact *p* = .07 and *p*<.05 respectively* | Mian (2015) |
|  | Parenting our Children to Excellence (PACE) | 322 | Study-specific questions answered by parent (either not currently employed or currently employed) | Maternal employment status (no stated direction) | No change | Wald χ^2^ = .13, OR = 1.14,  CI = [.56, 2.33] | *p* > .05* | Nordstrom (2008) |
| **Parent mental health status** | | | | | | | | |
|  | Program not named: once-off anxiety prevention seminar | 101 | Beck Anxiety Inventory (BAI) | Parent anxiety (no stated direction) | No change | Stat n/r | Non-sig* | Mian (2015) |
| **Parent’s perceived benefit of attending** | | | | | | | | |
|  | Parenting our Children to Excellence (PACE) | 322 | The Raising Young Children Scale | Parent's perceived benefits of the program (no stated direction) | Increase | Wald χ^2^ = 10.31,  OR = 1.08,  CI = [1.03, 1.13] | *p* < .001 | Nordstrom (2008) |
| **Measures of parenting behaviours** | | | | | | | | |
|  | Parenting our Children to Excellence (PACE) | 322 | Parenting Efficacy subscale of the Parenting Sense of Competence Scale | Parenting efficacy (no stated direction) | No change | Wald χ^2^ = 2.31,  OR = 1.04,  CI = [.99, 1.09] | *p* > .05 | Nordstrom (2008) |
|  | Parenting our Children to Excellence (PACE) | 322 | Parenting Possibilities Questionnaire, Family Stories measure | Positive attributions (no stated direction) | No change | Wald χ^2^ = .87, OR = 1.28,  CI = [.76, 2.17] | *p* > .05 | Nordstrom (2008) |
|  | Parenting our Children to Excellence (PACE) | 322 | Parenting Possibilities Questionnaire, Family Stories measure | Negative attributions (no stated direction) | No change | Wald χ^2^ = 1.30,  OR = 1.34,  CI = [.81, 2.21] | *p* > .05 | Nordstrom (2008) |
| **3. Child factors** | | | | | | | | |
| **Child age** | | | | | | | | |
|  | Prevention Program for Externalizing Problem Behaviour (PEP) | 2123 | Child’s age as reported by teacher | Older child | Decreased | Stat n/r | *p* = .015* | Plueck (2010) |
|  | Program not named: once-off anxiety prevention seminar | 101 | Study-specific questions answered by parent | Child age (no stated direction) | No change | Stat n/r | Non-sig* | Mian (2015) |
|  | Parenting our Children to Excellence (PACE) | 322 | Study-specific questions answered by parent | Child age (no stated direction) | No change | Wald χ^2^ = 2.90,  OR = .76,  CI = [.55, 1.04] | *p* > .05* | Nordstrom (2008) |
| **Child gender** | | | | | | | | |
|  | Prevention Program for Externalizing Problem Behaviour (PEP) | 2123 | Study-specific questions answered by parent | Child gender (male) | No change | Stat n/r | *p* = .623* | Plueck (2010) |
|  | Family Matters (FM) & Strengthening Families Program: For Parents and Youth 10-14 (SFP) | 214 | Study-specific questions answered by parent | Child gender (male) | No change | OR = .82 | *p* = .221* | Byrnes (2012) |
|  | Program not named: once-off anxiety prevention seminar | 101 | Study-specific questions answered by parent | Child gender (male) | No change | Stat n/r | Non-sig* | Mian (2015) |
|  | Parenting our Children to Excellence (PACE) | 322 | Study-specific questions answered by parent | Child gender (male) | No change | Wald χ^2^ = 3.09,  OR = .61,  CI = [.35, 1.06] | *p* > .05* | Nordstrom (2008) |
| **Mental health symptoms** | | | | | | | | |
|  | Prevention Program for Externalizing Problem Behaviour (PEP) | 2123 | Teacher-report - Child Behaviour Checklist | Increased child externalising behaviour | No change | Stat n/r | *p* = .079* | Plueck (2010) |
|  | Program not named: once-off anxiety prevention seminar | 101 | Brief Infant-Toddler Social and Emotional Assessment (BITSEA) | Child anxiety (no stated direction) | No change | Stat n/r | Non-sig* | Mian (2015) |
|  | Prevention Program for Externalizing Problem Behaviour (PEP) | 2123 | Teacher-report - Child Behaviour Checklist | Increased child internalising behaviours | No change | Stat n/r | *p* = .950* | Plueck (2010) |
|  | Parenting our Children to Excellence (PACE) | 322 | Disruptive Behaviour Disorders rating scale | ADHD symptoms (no stated direction) | No change | Wald χ^2^ = .56, OR = .98,  CI = [.94, 1.03] | *p* > .05* | Nordstrom (2008) |
|  | Parenting our Children to Excellence (PACE) | 322 | Disruptive Behaviour Disorders rating scale | ODD symptoms (no stated direction) | No change | Wald χ^2^ = .60, OR = .97,  CI = [.89, 1.05] | *p* > .05* | Nordstrom (2008) |
| **Child's exposure to trauma** | | | | | | | | |
|  | Program not named: once-off anxiety prevention seminar | 101 | Life Events Checklist | Increased child's exposure to previous trauma | No change | Stat n/r | Non-sig | Mian (2015) |
| **Perceived burden of child's behaviours** | | | | | | | | |
|  | Prevention Program for Externalizing Problem Behaviour (PEP) | 2123 | Teacher-reported burden for themselves due to child's behaviours | Increased burden of child's behaviours | No change | Stat n/r | *p* = .150 | Plueck (2010) |
|  | Prevention Program for Externalizing Problem Behaviour (PEP) | 2123 | Teacher’s reported need for additional assistance in the class room due to child’s problems | Increased need for help | Decreased | OR = 1.34,  CI = [1.09, 1.64] | *p* = .003 | Plueck (2010) |
| **4. Parent/child relational factors** | | | | | | | | |
| Nil Reported | | | | | | | | |
| **5. Barriers to engagement/service preferences** | | | | | | | | |
| **Time and scheduling difficulties** | | | | | | | | |
|  | Parenting our Children to Excellence (PACE) | 322 | The Obstacles to Engagement Scale (OES) | Fewer time/scheduling barriers | Increase | Wald χ^2^ = 38.88,  OR = 1.93,  CI = [1.57, 2.37] | *p* < .001 | Nordstrom (2008) |
|  | Parenting our Children to Excellence (PACE) | 322 | The Obstacles to Engagement Scale (OES) | Less personal obstacles | No change | Wald χ^2^ = 2.03,  OR = .91,  CI = [.79, 1.04] | *p* > .05 | Nordstrom (2008) |
|  | Parenting our Children to Excellence (PACE) | 322 | The Obstacles to Engagement Scale (OES) | Low intervention demands | No change | Wald χ^2^ = .58, OR = .94,  CI = [.81, 1.10] | *p* > .05 | Nordstrom (2008) |
| **Service preferences** | | | | | | | | |
|  | Program not named: once-off anxiety prevention seminar | 101 | Parent service preferences | Session type - parents who preferred "a group that teaches skills for parenting" | Decrease | 16.3% vs 45.0%,  χ^2^ = 6.29 | *p* < .05 | Mian (2015) |
|  | Program not named: once-off anxiety prevention seminar | 101 | Parent service preferences | Session topic – parents who preferred a topic of "healthy living and wellbeing" | Decrease | 26.5% vs 55.0%,  χ^2^ = 5.08 | *p* < .05 | Mian (2015) |
|  | Program not named: once-off anxiety prevention seminar | 101 | Parent service preferences | Previous service utilisation | No change | χ^2^ = 1.65 | *p* > .05 | Mian (2015) |
|  | Program not named: once-off anxiety prevention seminar | 101 | Parent service preferences | Group format | No change | Stat n/r | Non-sig | Mian (2015) |
|  | Program not named: once-off anxiety prevention seminar | 101 | Parent service preferences | Offer of incentives | No change | Stat n/r | Non-sig | Mian (2015) |
|  | Program not named: once-off anxiety prevention seminar | 101 | Parent service preferences | Group characteristics | No change | Stat n/r | Non-sig | Mian (2015) |
|  | Parenting our Children to Excellence (PACE) | 322 | The Obstacles to Engagement Scale (OES) | Program relevance/trust | No change | Wald χ^2^ = .74, OR = 1.07,  CI = [.92, 1.23] | *p* > .05 | Nordstrom (2008) |

Notes:

*Indicates *p*-values selected for Stouffer’s *p* analysis

## Table 4b

## *Descriptive Summary of the Predictors of Enrolment*

|  | **Parenting program name** | ***n*=** | | **Instrument** | **Predictor of engagement** | **Enrolment rate** | **Statistics** | | **Significance** | | | **Study** | |
| --- | --- | --- | --- | --- | --- | --- | --- | --- | --- | --- | --- | --- | --- |
| **1. Demographics** | | | | | | | | | | | | | |
| **Total household income** | | | | | | | | | | | | | |
|  | Bridges to High School | 353 | | Study-specific questions answered by parent (family income included an aggregate of wages, salary, child support and state assistance) | Family income (no stated direction) | No change | Wald χ^2^ = .49,  SE = .01,  Beta = .01 | | | *p* > .05 | | Carpentier (2007) | |
|  | Common Sense Parenting (CSP) | 213 | | Study-specific questions answered by parent (19-point scale from <10,000 to over 200,00) | Higher household income | Increased | OR = 1.50,  CI = [1.09, 2.06] | | | | *p* < .05 | Fleming (2015) | |
|  | Parenting our Children to Excellence (PACE) | 322 | | Yearly income (converted to a 4-point scale) | Family income (no stated direction) | No change | Wald χ^2^ = .05, OR = .97,  CI = [.74, 1.28] | | | | *p* > .05 | Nordstrom (2008) | |
| **Individual socioeconomic status** | | | | | | | | | | | | | |
|  | Triple P | 257 | | International Socio-Economic Index of occupational status | High SES | Increased | OR = 2.15,  CI = [1.38, 3.35] | | | | *p* < .001 | Eisner (2011) | |
|  | Prevention Program for Externalizing Problem Behaviour (PEP) | 91 | | parent-report: calculated as a mean of both parents’ education and profession | SES (no stated direction) | No change | Stat n/r | | | | *p* = .956 | Plueck (2010) | |
|  | Triple P | 282 | | Study-specific questions answered by teacher | Middle SES family or neighbourhood | Decreased | OR = .50,  CI = [.34, .73] and  OR = .69,  CI = [.49, .97], respectively | | | | *p* < .05 | Heinrichs (2005) | |
|  | Webster-Stratton’s Incredible Years program | 106 | | Ranked as high or low by neighbourhood SES of childcare | High SES | Increased | χ^2^ (1) = 15.42 | | | | *p* < .001 | Baker (2011) | |
|  | Program for mothers who have recently divorced (unnamed) | 321 | | Parent-report of household income divided by US Census Bureau's poverty threshold | Higher income-needs ratio | Increased | *b* = .27,  SE = .10,  Beta = .31 | | | | *p* < .05 | Winslow (2009) | |
|  | Triple P | 282 | | Study-specific questions answered by teacher | Low SES family or neighbourhood | Decreased | OR = .27,  CI = [.14, .51] and  OR = .49,  CI = [.34, .72], respectively | | | | *p* < .05 | Heinrichs (2005) | |
| **Neighbourhood socioeconomic status** | | | | | | | | | | | | | |
|  | Triple P | 282 | | Social structure index | High or moderate social problems | Decreased | χ^2^ (2) = 31.2 | | | | *p* < .001 | Heinrichs (2005) | |
|  | Prevention Program for Externalizing Problem Behaviour (PEP) | 91 | | Social burden of district (calculated by Department of Youth and Family Welfare) | Increased social burden of district | No change | | Stat n/r | | | *p* = .210 | Plueck (2010) | |
|  | Family Matters (FM) & Strengthening Families Program: For Parents and Youth 10-14 (SFP) | 214 | | 2000 Census of Population and Housing | Higher neighbourhood % below poverty line | No change | OR = 1.09 | | | | *p* = .743 | Byrnes (2012) | |
|  | Family Matters (FM) & Strengthening Families Program: For Parents and Youth 10-14 (SFP) | 214 | | 2000 Census of Population and Housing | Higher neighbourhood % on public assistance | No change | OR = 1.36 | | | | *p* = .165 | Byrnes (2012) | |
| **Neighbourhood unemployment** | | | | | | | | | | | | | |
|  | Family Matters (FM) & Strengthening Families Program: For Parents and Youth 10-14 (SFP) | 214 | | 2000 Census of Population and Housing | Higher levels of neighbourhood unemployment | No change | OR = 1.13 | | | | *p* = .485 | Byrnes (2012) | |
| **Transient nature of neighbourhood** | | | | | | | | | | | | | |
|  | Family Matters (FM) & Strengthening Families Program: For Parents and Youth 10-14 (SFP) | 214 | | 2000 Census of Population and Housing | Higher neighbourhood % of families moved in last 5 years | No change | OR = 1.09 | | | | *p* = .561 | Byrnes (2012) | |
| **Family Structure** | | | | | | | | | | | | | |
|  | Triple P | 282 | | Study-specific questions answered by teacher | Number of family members in household (no stated direction) | No change | Stat n/r | | | | Non-sig* | Heinrichs (2005) | |
|  | Family Matters (FM) & Strengthening Families Program: For Parents and Youth 10-14 (SFP) | 214 | | 2000 Census of Population and Housing | Neighbourhood % of female headed households | No change | OR = .85 | | | | *p* = .317 | Byrnes (2012) | |
|  | Strengthening Families Program: For Parents and Youth 10-14 (SFP) | 200 | | Study-specific questions answered by parent | Parent and child living together | No change | OR =1.45,  CI = [.75, 2.81] | | | | *p* > .05* | Skarstrand (2009) | |
|  | Bridges to High School | 353 | | Study-specific questions answered by parent | Number of kids in the home (no stated direction) | No change | Wald χ^2^ = .33, SE = .09,  Beta = .05 | | | | *p* > .05* | Carpentier (2007) | |
|  | Triple P | 257 | | Study-specific questions answered by parent | Large family (3+ children) | Decreased | OR = .50,  CI = [.30, .81] | | | | *p* < .01* | Eisner (2011) | |
| **One- or two-parent households** | | | | | | | | | | | | | |
|  | Triple P | 282 | | Study-specific questions answered by teacher | Single parent home | Increased | OR = 1.56, CI = [1.05, 2.32] | | | | *p* < .05* | Heinrichs (2005) | |
|  | Prevention Program for Externalizing Problem Behaviour (PEP) | 91 | | Study-specific questions answered by parent | Single parent home | No change | Stat n/r | | | | *p* = .854* | Plueck (2010) | |
|  | Webster-Stratton’s Incredible Years program | 106 | | Study-specific questions answered by parent | Single parent status | No change | χ^2^ (1) = 1.19 | | | | *p* = .28* | Baker (2011) | |
|  | Parenting our Children to Excellence (PACE) | 322 | | Study-specific questions answered by parent | Marital status (single parent) | No change | Wald χ^2^ = 3.16, OR = .53,  CI = [.26, 1.07] | | | | *p* > .05* | Nordstrom (2008) | |
|  | Common Sense Parenting (CSP) | 213 | | Study-specific questions answered by parent | Parent living with partner | No change | OR = 1.66,  CI = [.90, 3.07] | | | | *p* > .05* | Fleming (2015) | |
|  | Triple P | 257 | | Study-specific questions answered by parent | Single parent | No change | OR = .82,  CI = [.48, 1.40] | | | | *p* > .05* | Eisner (2011) | |
|  | Bridges to High School | 353 | | Study-specific questions answered by parent | Single parent status | No change | Wald χ^2^ = .86, SE = .39,  Beta = -.36 | | | | *p* > .05* | Carpentier (2007) | |
| **2. Parent factors** | | | | | | | | | | | | | |
| **Parent age** | | | | | | | | | | | | | |
|  | Family Matters (FM) & Strengthening Families Program: For Parents and Youth 10-14 (SFP) | 214 | | Study-specific questions answered by parent | Older parents | Increased | OR = 1.04 | | | | *p* = .037* | Byrnes (2012) | |
|  | Parenting our Children to Excellence (PACE) | 322 | | Study-specific questions answered by parent | Lower maternal age | Increased | Wald χ^2^ (1, N = 322) = 8.30 | | | | *p* < .004* | Nordstrom (2008) | |
|  | Prevention Program for Externalizing Problem Behaviour (PEP) | 91 | | Study-specific questions answered by parent | Parent’s age | No change | Stat n/r | | | | *p* = .063* | Plueck (2010) | |
|  | Triple P | 282 | | Study-specific questions answered by teacher | Parent’s age (no stated direction) | No change | Stat n/r | | | | Non-sig* | Heinrichs (2005) | |
|  | Common Sense Parenting (CSP) | 213 | | Study-specific questions answered by parent | Parent’s age (no stated direction) | No change | OR = 1.36,  CI = [.97, 1.90] | | | | *p* > .05* | Fleming (2015) | |
|  | Strengthening Families Program: For Parents and Youth 10-14 (SFP) | 200 | | Study-specific questions answered by parent | Parent’s age (no stated direction | No change | OR=1.06,  CI = [.78, 1.43] | | | | *p* > .05* | Skarstrand (2009) | |
| **Parent gender** | | | | | | | | | | | | | |
|  | Common Sense Parenting (CSP) | 213 | | Study-specific questions answered by parent | Parent male | No change | OR = .54,  CI = [.25, 1.16] | | | | *p* > .05* | Fleming (2015) | |
|  | Strengthening Families Program: For Parents and Youth 10-14 (SFP) | 200 | | Study-specific questions answered as parent | Parent male | No change | OR =1.05,  CI = [.54, 2.03] | | | | *p* > .05* | Skarstrand (2009) | |
| **Parent race/ethnicity** | | | | | | | | | | | | | |
|  | Webster-Stratton’s Incredible Years program | 106 | | Study-specific questions answered by parent | Caucasian families versus African American and Puerto Rican families | Increased | χ^2^ (1) = 8.60 and χ^2^ (1) = 14.95, respectively | | | | *p* <.01 and *p* <.001 respectively* | Baker (2011) | |
|  | Common Sense Parenting (CSP) | 213 | | Study-specific questions answered by parent | Parent race (Hispanic) | No change | OR = .88,  CI = [.34, 2.28] | | | | *p* > .05* | Fleming (2015) | |
|  | Strengthening Families Program: For Parents and Youth 10-14 (SFP) | 200 | | Study-specific questions answered by parent (‘Where you born in Sweden?’) | Born in Sweden | No change | OR = 1.70,  CI = [.81, 3.56] | | | | *p* > .05* | Skarstrand (2009) | |
|  | Program for mothers who have recently divorced (unnamed) | 321 | | Study-specific questions answered by parent (parent’s self-reported ethnicity) | Parent being of minority ethnicity | No change | *b* = .24,  SE = .05,  Beta = .07 | | | | *p* > .05* | Winslow (2009) | |
|  | Bridges to High School | 353 | | Acculturation Rating Scale for Mexican Americans-II | Primary parent Anglo orientation | No change | Wald χ^2^ = 1.60, SE = .25,  Beta = -.32 | | | | *p* > .05* | Carpentier (2007) | |
|  | Bridges to High School | 353 | | 16-item composite of three subscales (obligations to family, level of emotional closeness, using family as referent in decision-making) | Primary parent familism | No change | Wald χ^2^ = .30, SE = .39,  Beta = -.21 | | | | *p* > .05 | Carpentier (2007) | |
|  | Bridges to High School | 353 | | 16-item composite of three subscales (obligations to family, level of emotional closeness, using family as referent in decision-making) | Child Anglo orientation | No change | Wald χ^2^ = 1.50, SE = .25,  Beta = -.31 | | | | *p* > .05 | Carpentier (2007) | |
|  | Bridges to High School | 353 | | Acculturation Rating Scale for Mexican Americans-II | Child familism (no stated direction) | No change | Wald χ^2^ = .03, SE = .34,  Beta = -.06 | | | | *p* > .05 | Carpentier (2007) | |
|  | Parenting our Children to Excellence (PACE) | 322 | | Study-specific questions answered by parent (choice of African American or European American) | Maternal ethnicity (no stated direction) | No change | Wald χ^2^ = 3.20, OR = 1.85,  CI = [.94, 3.62] | | | | *p* > .05* | Nordstrom (2008) | |
| **Parent language proficiency** | | | | | | | | | | | | | |
|  | Bridges to High  School | 596 | | Study-specific questions  answered by parent | Family language preference (Spanish) | Increased | Wald χ^2^ (1) = 15.28,  Beta = -.56,  SE = .14,  Beta = .57 | | | | *p* < .001 | Carpentier (2007) | |
| **Parent education** | | | | | | | | | | | | | |
|  | Webster-Stratton’s Incredible Years program | 189 | | Parent-report versus Statistics Norway (2010) | Educated parents (bachelor degree or higher) | No change | 78% vs 41% | | | | *p* > .05* | Reedtz (2011) | |
|  | Common Sense Parenting (CSP) | 213 | | 8-point measure ranging from ‘some high school’ to ‘PhD, J.D, D.D.S, M.D, D.V.M’ | Higher parent education | Increased | OR = 1.48,  CI = [1.00, 2.14] | | | | *p* < .05* | Fleming (2015) | |
|  | Family Matters (FM) & Strengthening Families Program: For Parents and Youth 10-14 (SFP) | 214 | | 2000 Census of Population and Housing | Higher neighbourhood % high school dropout (parent’s generation) | Decrease | OR = .56 | | | | *p* = .006 | Byrnes (2012) | |
|  | Program for mothers who have recently divorced (unnamed) | 321 | | Study-specific questions answered by parent (ordinal scale i.e. elementary, some high school, graduation) | Maternal education (no stated direction) | No change | *b* = .06,  SE = .05,  Beta = .10 | | | | *p* > .05* | Winslow (2009) | |
|  | Strengthening Families Program: For Parents and Youth 10-14 (SFP) | 200 | | Study-specific questions answered by parent | Parent education (no stated direction) | No change | OR = 1.29,  CI = [.85, 1.95] | | | | *p* > .05* | Skarstrand (2009) | |
|  | Bridges to High School | 353 | | Highest education level obtained by any primary caregiver in family | Family education level (no stated direction) | No change | Wald χ^2^ = 1.96, SE = .05,  Beta = -.07 | | | | *p* > .05* | Carpentier (2007) | |
|  | Parenting our Children to Excellence (PACE) | 322 | | Study-specific questions answered by parent (4-point scale from high school not completed to college graduate) | Maternal education (no stated direction) | No change | Wald χ^2^ = 3.76, OR = 1.48,  CI = [1.00, 2.20] | | | | *p* > .05* | Nordstrom (2008) | |
| **Parent occupation** | | | | | | | | | | | | | |
|  | Strengthening Families Program: For Parents and Youth 10-14 (SFP) | 200 | | Study-specific questions answered by parent | Working full-time | No change | OR = 1.65,  CI = [.73, 3.75] | | | | *p* > .05 | Skarstrand (2009) | |
|  | Triple P | 282 | | Study-specific questions answered by teacher (parents’ occupation placed in categories of workers, employers, public servant, self-employed, other) | Parent occupation type (no stated direction) | No change | Stat n/r | | | | Non-sig* | Heinrichs (2005) | |
|  | Bridges to High School | 353 | | Study-specific questions answered by parent (parents asked to report how many hours worked a week) | Number of parent hours worked | No change | Wald χ^2^ = .08, SE = .01,  Beta = .01 | | | | *p* > .05* | Carpentier (2007) | |
|  | Parenting our Children to Excellence (PACE) | 322 | | Study-specific questions answered by parent (either currently not employed or currently employed) | Maternal employment status (no stated direction) | No change | Wald χ^2^ = 1.63, OR = 1.63,  CI = [.77, 3.45] | | | | *p* > .05* | Nordstrom (2008) | |
|  | Triple P | 257 | | Study-specific questions answered by parent | Dual-earner family | Decreased | OR = .46,  CI = [.29, .73] | | | | *p* < .001* | Eisner (2011) | |
| **Parental mental health status** | | | | | | | | | | | | | |
|  | Program for mothers who have recently divorced (unnamed) | 321 | | Psychiatric Epidemiology Research Interview (PERI) Demoralisation scale | Maternal distress (no stated direction) | No change | *b* = .00,  SE = .01,  Beta = .02 | | | | *p* > .05* | Winslow (2009) | |
|  | Webster-Stratton’s Incredible Years program | 106 | | Brief Symptom Inventory (BSI) | Increased parent depression score | No change | *t* (76) = -.48 | | | | *p* = .63* | Baker (2011) | |
| **Months since divorce** | | | | | | | | | | | | | |
|  | Program for mothers who have recently divorced (unnamed) | 321 | | Study-specific questions answered by parent | Increased number of months since divorce | No change | *b* = -.03,  SE = .02,  Beta = -.15 | | | | *p* > .05 | Winslow (2009) | |
| **Parental perceived benefit** | | | | | | | | | | | | | |
|  | Parenting our Children to Excellence (PACE) | 322 | | The Raising Young Children Scale | Higher perceived benefits | Increased | Wald χ^2^ (1, N = 322) = 6.64 | | | | *p* < .010 | Nordstrom (2008) | |
| **Parental self efficacy** | | | | | | | | | | | | | |
|  | Parenting our Children to Excellence (PACE) | 322 | | Parenting Efficacy subscale of the Parenting Sense of Competence Scale | Higher parental self-efficacy | Increased | Wald χ^2^ (1, N = 322) = 6.37 | | | | *p* < .012 | Nordstrom (2008) | |
| **Measures of parenting behaviours** | | | | | | | | | | | | | |
|  | Program for mothers who have recently divorced (unnamed) | 321 | | Composite of self-report scales (CRPBI Inconsistent Discipline subscale, Oregon Social Learning Centre ratio of inappropriate-to-appropriate discipline and follow-through scales) | Effective discipline | No change | *b* = .020,  SE = .17,  Beta = .13 | | | | *p* > .05 | Winslow (2009) | |
|  | Strengthening Families Program: For Parents and Youth 10-14 (SFP) | 200 | | Parent-report of rule-setting in home | Increased rule-setting by parents | No change | OR = .99,  CI = [.59, 1.67] | | | | *p* > .05 | Skarstrand (2009) | |
|  | Strengthening Families Program: For Parents and Youth 10-14 (SFP) | 200 | | Study-specific questions answered by parent | Knowledge of school performance | No change | OR = .84,  CI = [.49, 1.43] | | | | *p* > .05 | Skarstrand (2009) | |
|  | Strengthening Families Program: For Parents and Youth 10-14 (SFP) | 200 | | Study-specific questions answered by parent | More restrictive attitude to alcohol | Increase | OR = 2.03,  CI = [1.02, 4.06] | | | | *p* < .05 | Skarstrand (2009) | |
|  | Parenting our Children to Excellence (PACE) | 322 | | Parenting Possibilities Questionnaire, Family Stories measure | Positive attributions (not stated direction) | No change | Wald χ^2^ = .41, OR = 1.20,  CI = [.69, 2.07] | | | | *p* > .05 | Nordstrom (2008) | |
|  | Parenting our Children to Excellence (PACE) | 322 | | Parenting Possibilities Questionnaire, Family Stories measure | Negative attributions (no stated direction) | No change | Wald χ^2^ = .13, OR = .91,  CI = [.54, 1.52] | | | | *p* > .05 | Nordstrom (2008) | |
|  | Triple P | 257 | | Alabama Parenting Questionnaire | Parenting problems (no stated direction) | No change | OR = 1.25,  CI = [.85, 1.86] | | | | *p* > .05 | Eisner (2011) | |
| **Parental social support** | | | | | | | | | | | | | |
|  | Webster-Stratton’s Incredible Years program | 106 | | Social Support Appraisals Scale (SSAS) | Greater social support | Increased | *t* (74) = -2.66 | | | | *p* = .01 | Baker (2011) | |
|  | Triple P | 257 | | Study-specific questions answered by parent | Strong neighbourhood social networks | Increased | OR = 1.58,  CI = [1.05, 2.37] | | | | *p* < .05 | Eisner (2011) | |
| **3. Child factors** | | | | | | | | | | | | | |
| **Child age** | | | | | | | | | | | | | |
|  | Common Sense Parenting (CSP) | 213 | | Study-specific questions answered by parent | Younger child | Increased | OR = .70,  CI = [.51, .95] | | | | *p* < .05* | Fleming (2015) | |
|  | Prevention Program for Externalizing Problem Behaviour (PEP) | 91 | | Study-specific questions answered by teacher | Child's age (no stated direction) | No change | Stat n/r | | | | *p* = .841* | Plueck (2010) | |
|  | Parenting our Children to Excellence (PACE) | 322 | | Study-specific questions answered by parent | Child age (no stated direction) | No change | Wald χ^2^ = .60, OR = .87,  CI = [.62, 1.23] | | | | *p* > .05* | Nordstrom (2008) | |
| **Child gender** | | | | | | | | | | | | | |
|  | Prevention Program for Externalizing Problem Behaviour (PEP) | 91 | | Study-specific questions answered by parent | Child gender (male) | No change | OR = .08 | | | | *p* = .061* | Plueck (2010) | |
|  | Family Matters (FM) & Strengthening Families Program: For Parents and Youth 10-14 (SFP) | 214 | | Study-specific questions answered by parent | Child’s gender (no stated direction) | No change | OR = .77 | | | | *p* = .298* | Byrnes (2012) | |
|  | Common Sense Parenting (CSP) | 213 | | Study-specific questions answered by parent | Child’s gender (male) | No change | OR = 1.28,  CI = [.69, 2.37] | | | | *p* > .05* | Fleming (2015) | |
|  | Strengthening Families Program: For Parents and Youth 10-14 (SFP) | 200 | | Study-specific questions answered by parent | Child’s gender (male) | No change | OR = 1.00,  CI = [.61, 1.66] | | | | *p* > .05* | Skarstrand (2009) | |
|  | Parenting our Children to Excellence (PACE) | 322 | | Study-specific questions answered by parent | Child gender (no stated direction) | No change | Wald χ^2^ = .04, OR = .94,  CI = [.54, 1.66] | | | | *p* > .05* | Nordstrom (2008) | |
| **Child’s academic success** | | | | | | | | | | | | | |
|  | Common Sense Parenting (CSP) | 213 | | Study-specific questions answered by child | Child's school grades (no stated direction) | No change | OR = 1.29,  CI = [.96, 1.75] | | | | *p* > .05 | Fleming (2015) | |
|  | Bridges to High School | 353 | | Letter grades aggregated across quarters to yield a GPA | Child GPA (no stated direction) | No change | Wald χ^2^ = .03, SE = .01,  Beta = -.05 | | | | *p* > .05 | Carpentier (2007) | |
| **Child mental health symptoms** | | | | | | | | | | | | | |
|  | Webster-Stratton’s Incredible Years program | 189 | | ECBI (Intensity and Problem subscales) versus Norwegian norm | Increased externalising problem behaviours | Increased | Intensity; *t*=7.7  Problem; *t*=7.1 | | | | Both *p*’s  < .001* | Reedtz (2011) | |
|  | Parenting our Children to Excellence (PACE) | 322 | | Disruptive Behaviour Disorders rating scale | More child ODD symptoms | Increased | Wald χ^2^ (1, N = 322) = 11.62 | | | | *p* < .001* | Nordstrom (2008) | |
|  | Prevention Program for Externalizing Problem Behaviour (PEP) | 91 | | Parent-report (pre-test): Child Behaviour Checklist | Less child externalising behaviours | Decreased | OR = .88 | | | | *p* = .044* | Plueck (2010) | |
|  | Webster-Stratton’s Incredible Years program | 106 | | Teacher’s Report Form (TRF) | Increased child externalising behaviours | No change | *t* (97) = -.58 | | | | *p* = .57* | Baker (2011) | |
|  | Common Sense Parenting (CSP) | 213 | | Strengths and Difficulties Questionnaire – Conduct Problems scale | Child conduct problems (no stated direction) | No change | OR = 1.08,  CI = [.79, 1.48] | | | | *p* > .05* | Fleming (2015) | |
|  | Prevention Program for Externalizing Problem Behaviour (PEP) | 91 | | Parent-report (screening): Child Behaviour Checklist | Child externalising behaviours (no stated direction) | No change | Stat n/r | | | | *p* = .976 | Plueck (2010) | |
|  | Prevention Program for Externalizing Problem Behaviour (PEP) | 91 | | Teacher-report (screening): Child Behaviour Checklist | Child externalising behaviours (no stated direction) | No change | Stat n/r | | | | *p* = .460 | Plueck (2010) | |
|  | Bridges to High School | 353 | | Externalising score on Child Behaviour Checklist (CBCL) | Child externalising symptoms (no stated direction) | No change | Wald χ^2^ = .09, SE = .02,  Beta = -.01 | | | | *p* > .05* | Carpentier (2007) | |
|  | Triple P | 257 | | Social Behaviour Questionnaire (Externalising Problem Behaviour subscale) | Child externalising problem behaviour (no stated direction) | No change | OR = 1.03,  CI = [.69, 1.54] | | | | *p* > .05* | Eisner (2011) | |
|  | Parenting our Children to Excellence (PACE) | 322 | | Disruptive Behaviour Disorders rating scale | ADHD symptoms (no stated direction) | No change | Wald χ^2^ = .38, OR = .99,  CI = [.95, 1.03] | | | | *p* > .05* | Nordstrom (2008) | |
|  | Prevention Program for Externalizing Problem Behaviour (PEP) | 91 | | Parent-report (pre-test): Child Behaviour Checklist | Child internalising behaviours (no stated direction) | No change | Stat n/r | | | | *p* = .900* | Plueck (2010) | |
|  | Prevention Program for Externalizing Problem Behaviour (PEP) | 91 | | Parent-report (screening): Child Behaviour Checklist | Child internalising behaviours (no stated direction) | No change | OR = 2.00 | | | | *p* = .076 | Plueck (2010) | |
|  | Prevention Program for Externalizing Problem Behaviour (PEP) | 91 | | Teacher-report (screening): Child Behaviour Checklist | Child internalising behaviours (no stated direction) | No change | Stat n/r | | | | *p* = .315 | Plueck (2010) | |
|  | Common Sense Parenting (CSP) | 213 | | Strengths and Difficulties Questionnaire – emotional symptoms scale | Child emotional symptoms (no stated direction) | No change | OR = .84,  CI = [.63, 1.13] | | | | *p* > .05* | Fleming (2015) | |
|  | Bridges to High School | 353 | | Internalising score on Child Behaviour Checklist (CBCL) | Child internalising symptoms (no stated direction) | No change | Wald χ^2^ = .01, SE = .02,  Beta = .01 | | | | *p* > .05* | Carpentier (2007) | |
|  | Program for mothers who have recently divorced (unnamed) | 321 | | Child Behaviour Checklist (CBCL) | Higher child maladjustment | Increased | *b* = .03,  SE = .01,  Beta = .23 | | | | *p* < .05* | Winslow (2009) | |
|  | Strengthening Families Program: For Parents and Youth 10-14 (SFP) | 200 | | Study-specific questions answered by parent (14 statements which made a ‘perception of norm-breaking behaviours’) | Child's norm-breaking behaviours (no stated direction) | No change | OR = .94,  CI = [.61, 1.45] | | | | *p* > .05* | Skarstrand (2009) | |
| **Child's exposure to negative life events** | | | | | | | | | | | | | |
|  | Program for mothers who have recently divorced (unnamed) | 321 | | Negative Life Events Scale | Increased child's exposure to negative life events | No change | *b* = .01,  SE = .03,  Beta = .03 | | | | *p* > .05 | Winslow (2009) | |
| **Academic success** | | | | | | | | | | | | | |
|  | Common Sense Parenting (CSP) | 213 | | Study-specific questions answered by child | Child's school grades (no stated direction) | No change | OR = 1.29,  CI = [.96, 1.75] | | | | *p* > .05 | Fleming (2015) | |
|  | Bridges to High School | 353 | | Letter grades aggregated across quarters to yield a GPA | Child GPA (no stated direction) | No change | Wald χ^2^ = .03, SE = .01,  Beta = -.05 | | | | *p* > .05 | Carpentier (2007) | |
| **Perceived burden of child’s behaviour** | | | | | | | | | | | | | |
|  | Prevention Program for Externalizing Problem Behaviour (PEP) | 91 | | Study-specific questions answered by parent | Parent’s increased need for assistance with child's behaviour | No change | Stat n/r | | | | *p* = .232 | Plueck (2010) | |
|  | Prevention Program for Externalizing Problem Behaviour (PEP) | 91 | | Teacher’s reported need for additional assistance in the class room due to child’s problems | Need for help | No change | Stat n/r | | | | *p* = .731 | Plueck (2010) | |
|  | Prevention Program for Externalizing Problem Behaviour (PEP) | 91 | | Teacher reported burden for themselves due to child's behaviours | Increased burden of child's behaviours | Decreased | Stat n/r | | | | *p* = .012 | Plueck (2010) | |
|  | Prevention Program for Externalizing Problem Behaviour (PEP) | 91 | | Parent-report of burden of child's behaviours | Increased burden of child's behaviours | No change | Stat n/r | | | | *p* = .711 | Plueck (2010) | |
| **4. Child/parent factors** | | | | | | | | | | | | | |
| **Parent-child affect quality** | | | | | | | | | | | | | |
|  | Common Sense Parenting (CSP) | 213 | | Parent-report of parent-child affect quality | Decreased parent-child affect quality | Increased | OR=.70,  CI = [.50, .96] | | | | *p* < .05 | Fleming (2015) | |
|  | Strengthening Families Program: For Parents and Youth 10-14 (SFP) | 200 | | Warmth of Parenting Scale | Decreased parent warmth | Decreased | OR=.48,  CI = [.29, .80] | | | | *p* < .01 | Skarstrand (2009) | |
|  | Program for mothers who have recently divorced (unnamed) | 321 | | Acceptance and Rejection subscales of the Child Report of Parenting Behaviour Inventory and the Parent-Adolescent Communication Scale | Mother-child relationship quality (on stated direction) | No change | *b* = -.05,  SE = .12,  Beta = -.04 | | | | *p* > .05 | Winslow (2009) | |
| **Family management** | | | | | | | | | | | | | |
|  | Common Sense Parenting (CSP) | 213 | | Alabama Parenting Questionnaire | Family management (no stated direction) | No change | OR = .99,  CI = [.73, 1.35] | | | | *p* > .05 | Fleming (2015) | |
| **5. Barriers to engagement/ service preferences** | | | | | | | | | | | | | |
| **time/scheduling barriers** | | | | | | | | | | | | | |
| Parenting our Children to Excellence (PACE) | | | 322 | The Obstacles to Engagement Scale (OES) | Less time/scheduling barriers | Increased | | Wald χ^2^ (1, N = 322) = 27.43 | | | *p* < .001 | | Nordstrom (2008) |
| Parenting our Children to Excellence (PACE) | | | 322 | The Obstacles to Engagement Scale (OES) | Less personal and family obstacles | Increased | | Wald χ^2^ (1, N = 322) = 8.39 | | | *p* < .004 | | Nordstrom (2008) |
| **Service preferences** | | | | | | | | | | | | | |
| **Child and parent attending** | | | | | | | | | | | | | |
|  | Common Sense Parenting (CSP) | 213 | | Common Sense Parenting versus Common Sense Parenting Plus | Parents enrolled in CSP Plus | No change | OR = 1.64,  CI = [.88, 3.09] | | | | *p* > .05 | Fleming (2015) | |
| **Program relevance/trust** | | | | | | | | | | | | | |
|  | Parenting our Children to Excellence (PACE) | 322 | | The Raising Young Children Scale | More program relevance/trust | Increased | Wald χ^2^ (1, N = 322) = 5.15 | | | | *p* < .023 | Nordstrom (2008) | |
|  | Parenting our Children to Excellence (PACE) | 322 | | The Obstacles to Engagement Scale (OES) | Low intervention demands | No change | Wald χ^2^ = .01, OR = .99,  CI = [.84, 1.17] | | | | *p* > .05 | Nordstrom (2008) | |
|  | Triple P | 257 | | Study-specific questions answered by parent | Non-Triple P language (i.e. non-native language) | Decreased | OR = .46,  CI = [.28, .75] | | | | *p* < .01 | Eisner (2011) | |
|  | Triple P | 257 | | Study-specific questions answered by parent | Previous parent service utilisation | No change | OR = .99,  CI = [.64, 1.52] | | | | *p* > .05 | Eisner (2011) | |

Notes:

*Indicates *p*-values selected for Stouffer’s *p* analysis

## Table 4c

## *Descriptive summary of the predictors of ongoing engagement*

|  | **Parenting program name** | ***n*=** | | **Instrument** | **Predictor of engagement** | **Ongoing engagement rate** | **Statistics** | **Significance** | | | **Study** |
| --- | --- | --- | --- | --- | --- | --- | --- | --- | --- | --- | --- |
| **1. Demographic information** | | | | | | | | | | | |
| **total household income** | | | | | | | | | | | |
|  | Chicago Parent Program (CPP) | | 292 | Study-specific questions answered by parent | Financial parity (no stated direction | No change | Stat n/r | | Non-sig | | Garvey (2006) |
|  | Triple P | | 282 | Study-specific questions answered by teacher | Parent income (no stated direction) | No change | Stat n/r | | Non-sig | | Heinrichs (2005) |
|  | Common Sense Parenting (CSP) | | 157 | 19-point measure with categories ranging from <$10,000 to over $200,000 | Total household income | No change | Beta = 3.31,  SE = 2.34 | | *p* > .05 | | Fleming (2015) |
|  | Program not named: once-off anxiety prevention seminar | | 101 | Family income (converted to a 5-point scale) | Parent income (no stated direction) | No change | Stat n/r | | Non-sig | | Mian (2015) |
|  | Parenting our Children to Excellence (PACE) | | 114 | Yearly income (converted to a 4-point scale) | Family income (no stated direction) | No change | Beta = .37,  SE = .27,  *t* (93) = 1.40 | | Non-sig | | Nordstrom (2008) |
|  | Triple P | | 257 | Study-specific questions answered by parent | Dual-earner family | Decreased | OR = .23,  CI = [.06, .80] | | *p* < .05 | | Eisner (2011) |
|  | Bridges to High School | | 353 | Study-specific questions answered by parent (family income included an aggregate of wages, salary, child support and state assistance) | Family income (no stated direction) | No change | *b* = .01,  SE = .01,  Beta = .03 | | Non-sig | | Carpentier (2007) |
| **Individual socioeconomic status** | | | | | | | | | | | |
|  | Prevention Program for Externalizing Problem Behaviour (PEP) | | 74 | Parent-report: calculated as a mean of both parents’ education and profession | Low SES | Decreased | OR = .25 | | *p* = .001 | | Plueck (2010) |
|  | Webster-Stratton’s Incredible Years program | | 106 | High or low SES based on childcare attending | Low SES | No change | *t* (49) = -.76 | | *p* = .45 | | Baker (2011) |
|  | Chicago Parent Program (CPP) | | 292 | Parent-report (parents indicated whether any of 7 possible events occurred to them in last year, i.e. being unable to pay rent/mortgage) | Level of economic disadvantage | No change | Stat n/r | | Non-sig | | Garvey (2006) |
|  |  | |  |  |  |  |  | |  | |  |
|  | Barkley (1997)’s Behavioural Parent Training (BPT) program | | 72 | Study-specific questions answered by parent | Low SES | No change | Stat n/r | | Non-sig | | Hellenthal (2009) |
|  | Bridges to High School | | 292 | Study-specific questions answered by parent | Assessed as one factor: family income, child support required and state and federal assistance | No change | Stat n/r | | Non-sig | | Mauricio (2014) |
|  | Program for mothers who have recently divorced (unnamed) | | 321 | Parent-report of household income and dividing it by US Census Bureau's poverty threshold | Income-needs ratio | No change | *b* = .08,  SE = .11,  Beta = .09 | | *p* > .05 | | Winslow (2009) |
|  | Strong African American Families (SAAF) | | 172 | Money for Necessities subscale from the Family Resource Scale | Perceived economic stress | No change | Stat n/r | | Non-sig | | Brody (2006) |
|  | Triple P | | 257 | International Socio-Economic Index of occupational status | High SES | No change | OR = 2.27,  CI = [.68, 7.53] | | *p* > .05 | | Eisner (2011) |
| **Neighbourhood socioeconomic status** | | | | | | | | | | | |
|  | Triple P | 282 | | Social structure index of preschool (OKS) | Low SES neighbourhood | Decreased | OR = .31, CI = [.13, .75] | | *p* < .05 | | Heinrichs (2005) |
|  | Prevention Program for Externalizing Problem Behaviour (PEP) | 74 | | Social burden of district (calculated by Department of Youth and Family Welfare) | Social burden of district | No change | Stat n/r | | *p* = .290 | | Plueck (2010) |
| **Family Structure** | | | | | | | | | | | |
|  | Strong African American Families (SAAF) | 172 | | Ratio of children to adults calculated by dividing number of children in household by number of adults living there | Higher ratio of children to parents | Decreased | Beta = -.24 (SEM model; χ^2^ (40, N = 164) = 33.36, *p* = .76) | | *p* < .05* | | Brody (2006) |
|  | Strengthening Families Program: For Parents and Youth 10-14 (SFP) | 115 | | Study-specific questions answered by parent | Living with target child | No Change | OR = .84, CI = [.41, 1.74] | | *p* > .05* | | Skarstrand (2009) |
|  |  |  | |  |  |  |  | |  | |  |
|  | Triple P | 257 | | Study-specific questions answered by parent | Large family (3+ children) | Decreased | OR = .19,  CI = [.05, .75] | | *p* < .05* | | Eisner (2011) |
|  | Bridges to High School | 353 | | Study-specific questions answered by parent (parents asked how many children living at home) | Number of kids in the home | No change | *b* = -.06,  SE = .12,  Beta = -.02 | | *p* > .05* | | Carpentier (2007) |
|  |  |  | |  |  |  |  | |  | |  |
| **One- or two-parent households** | | | | | | | | | | | |
|  | Common Sense Parenting (CSP) | 157 | | Baseline parent interview | Parent living with partner | No change | Beta = .19,  SE = 4.69 | | *p* > .05* | | Fleming (2015) |
|  | Parenting our Children to Excellence (PACE) | 114 | | Study-specific questions answered by parent | Marital status (single parent) | No change | Beta = -1.09,  SE = .71,  *t* (93) = -1.55 | | Non-sig* | | Nordstrom (2008) |
|  | Triple P | 257 | | Study-specific questions answered by parent | Single parent | No change | OR = .84,  CI = [.13, 5.38] | | *p* > .05* | | Eisner (2011) |
|  | Bridges to High School | 353 | | Study-specific questions answered by parent | Single parent status | No change | *b* = -.39,  SE = .53,  Beta = -.04 | | *p* > .05* | | Carpentier (2007) |
|  | Prevention Program for Externalizing Problem Behaviour (PEP) | 74 | | Study-specific questions answered by parent | Single parent | No change | Stat n/r | | *p* = .746* | | Plueck (2010) |
|  | Chicago Parent Program (CPP) | 292 | | Study-specific questions answered by parents | Marital status (no stated direction) | No change | Stat n/r | | Non-sig* | | Garvey (2006) |
|  | Webster-Stratton’s Incredible Years program | 106 | | Study-specific questions answered by parents | Single parent status | Increase | *t* (46) = -2.85 | | *p* < .01* | | Baker (2011) |
| **2. Parent factors** | | | | | | | | | | | |
| **Parent age** | | | | | | | | | | | |
|  | Barkley (1997)’s Behavioural Parent Training (BPT) program | | 72 | Study-specific questions answered by parent | Younger parent age | Decreased | *r* = .36 | | *p* < .01* | Hellenthal (2009) | |
|  | Strengthening Families Program: For Parents and Youth 10-14 (SFP) | | 115 | Study-specific questions answered by parent | Parent age (no stated direction) | No change | OR = 1.33, CI = [.94, 1.87] | | *p* > .05* | Skarstrand (2009) | |
|  | Prevention Program for Externalizing Problem Behaviour (PEP) | | 74 | Study-specific questions answered by parent | Parent age (no stated direction) | No change | Stat n/r | | *p* = .349* | Plueck (2010) | |
|  | Chicago Parent Program (CPP) | | 292 | Study-specific questions answered by parent | Parent age (no stated direction) | No change | Stat n/r | | Non-sig* | Garvey (2006) | |
|  | Common Sense Parenting (CSP) | | 157 | Study-specific questions answered by parent | Parent age (no stated direction) | No change | Beta = 2.25,  SE = 2.25 | | *p* > .05* | Fleming (2015) | |
|  | Parenting our Children to Excellence (PACE) | | 114 | Study-specific questions answered by parent | Maternal age (no stated direction) | No change | Beta = -.14,  SE = .21,  *t* (93) = -.65 | | Non-sig* | Nordstrom (2008) | |
| **Parent gender** | | | | | | | | | | | |
|  | Strengthening Families Program: For Parents and Youth 10-14 (SFP) | 115 | | Study-specific questions answered by parent | Parent gender (male) | No change | OR = 1.01,  CI = [.51, 2.01] | | *p* > .05* | | Skarstrand (2009) |
|  | Common Sense Parenting (CSP) | 157 | | Study-specific questions answered by parent | Parent gender (male) | No change | Beta = 9.27,  SE = 6.51 | | *p* > .05* | | Fleming (2015) |
| **Parent race/ethnicity** | | | | | | | | | | | |
|  | Strengthening Families Program: For Parents and Youth 10-14 (SFP) | 115 | | Study-specific questions answered by parent | Birth place of parent (same country as study) | Increased | OR = 4.98,  CI = [1.62, 15.30] | | *p* < .01* | | Skarstrand (2009) |
|  | Webster-Stratton’s Incredible Years program | 106 | | Study-specific questions answered by parent | Ethnicity (African American versus Puerto Rican versus Caucasian) | No change | *F* (2,43) = 1.25 | | *p* = .30* | | Baker (2011) |
|  | Chicago Parent Program (CPP) | 292 | | Study-specific questions answered by parent | Ethnicity/race (no stated direction) | No change | Stat n/r | | Non-sig* | | Garvey (2006) |
|  | Common Sense Parenting (CSP) | 157 | | Baseline parent interview | Parent Race (Caucasian verses African American) | No change | Beta = 7.88,  SE = 5.41 | | *p* > .05* | | Fleming (2015) |
|  | Program for mothers who have recently divorced (unnamed) | 321 | | Study-specific questions answered by parent (self-report of ethnicity) | Parent minority ethnicity | No change | *b* = -.47,  SE = .30,  Beta = -.15 | | *p* > .05* | | Winslow (2009) |
|  | Parenting our Children to Excellence (PACE) | 114 | | Study-specific questions answered by parent (choice of African American or European American) | Maternal ethnicity (no stated direction) | No change | Beta = 1.08,  SE = .67,  *t* (93) = 1.62 | | Non-sig* | | Nordstrom (2008) |
|  | Bridges to High School | 353 | | Acculturation Rating Scale for Mexican Americans-II | Primary parent Anglo-orientation | No change | *b* = -.02,  SE = .34,  Beta = -.01 | | *p* > .05* | | Carpentier (2007) |
|  | Bridges to High School | 353 | | 16-item composite of three subscales (obligations to family, level of emotional closeness, using family as referent in decision-making) | Primary parent familism | No change | *b* = -.22,  SE = .52,  Beta = -.02 | | *p* > .05 | | Carpentier (2007) |
|  | Bridges to High School | 353 | | 16-item composite of three subscales (obligations to family, level of emotional closeness, using family as referent in decision-making) | Child Anglo orientation | No change | *b* = .05,  SE = .33,  Beta = .01 | | *p* > .05 | | Carpentier (2007) |
|  | Bridges to High School | 353 | | Acculturation Rating Scale for Mexican Americans-II | Child familism | No change | *b* = .61,  SE = .45,  Beta = .07 | | *p* > .05 | | Carpentier (2007) |
| **Parent language proficiency** | | | | | | | | | | | |
|  | Triple P | 257 | | Study-specific questions answered by parent | Non-Triple P language (i.e. non-native language) | Decreased | OR = .27,  CI = [.07, 1.13] | | *p* < .10 | | Eisner (2011) |
|  | Bridges to High  School | 353 | | Study-specific questions answered by parent | Family language preference (Spanish) | Increased | *b* = 1.46,  SE = .44,  Beta = .21 | | *p* < .01 | | Carpentier (2007) |
| **Parent education** | | | | | | | | | | | |
|  | Parenting our Children to Excellence (PACE) | 114 | | Study-specific questions answered by parent (4-point scale from high school not completed - college graduate) | Higher maternal education | Increased | Beta = .79,  *t* (93) = 2.23 | | *p* = .026* | | Nordstrom (2008) |
|  | Program for mothers who have recently divorced (unnamed) | 321 | | Study-specific questions answered by parent (ordinal scale i.e. elementary, some high school, graduation) | Higher maternal education | Increased | *b* = .13,  SE = .06,  Beta = .21 | | *p* < .05* | | Winslow (2009) |
|  | Strengthening Families Program: For Parents and Youth 10-14 (SFP) | 115 | | Study-specific questions answered by parent | Education level (no stated direction) | No change | OR = 1.48,  CI = [.93, 2.37] | | *p* > .05* | | Skarstrand (2009) |
|  | Chicago Parent Program (CPP) | 292 | | Study-specific questions answered by parent | Parent education level (no stated direction) | No change | Stat n/r | | Non-sig* | | Garvey (2006) |
|  | Barkley (1997)’s Behavioural Parent Training (BPT) program | 72 | | Study-specific questions answered by parent | Parent education (no stated direction) | No change | Stat n/r | | Non-sig* | | Hellenthal (2009) |
|  | Common Sense Parenting (CSP) | 157 | | 8-point measure ranging from ‘some high school’ to ‘PhD, J.D, D.D.S, M.D, D.V.M’ | Parent education (no stated direction) | No change | Beta = 3.94,  SE = 2.26 | | *p* > .05* | | Fleming (2015) |
|  | Bridges to High School | 353 | | Highest education level obtained by any primary caregiver in family | Family education level (no stated direction) | No change | *b* = .04,  SE = .06,  Beta = .04 | | *p* > .05* | | Carpentier (2007) |
| **Parent occupation** | | | | | | | | | | | |
|  | Strengthening Families Program: For Parents and Youth 10-14 (SFP) | 115 | | Study-specific questions answered by parent | Working full-time | No change | OR = 1.19,  CI = [.46, 3.05] | | *p* > .05* | Skarstrand (2009) | |
|  | Chicago Parent Program (CPP) | 292 | | Study-specific questions answered by parent | Maternal employment status (no stated direction) | No change | Stat n/r | | Non-sig* | Garvey (2006) | |
|  | Parenting our Children to Excellence (PACE) | 114 | | Study-specific questions answered by parent (choice of not currently employed or currently employed) | Maternal employment status (no stated direction) | No change | *b* = -.58,  SE = .89,  *t* (93) = -.65 | | Non-sig* | Nordstrom (2008) | |
|  | Bridges to High School | 353 | | Study-specific questions answered by parent | Number of parent hours worked | No change | *b* = -.01,  SE = .01,  Beta = -.02 | | *p* > .05* | Carpentier (2007) | |
| **Parent mental health status** | | | | | | | | | | | |
|  | Bridges to High School | 292 | | Centre for Epidemiologic Studies Depression Scale | Increased symptoms of depression | More likely to attend but drop out early | Logit = -0.03, SE = 0.01,  OR = .97 | | *p* < .05* | | Mauricio (2014) |
|  | Chicago Parent Program (CPP) | 292 | | Everyday Stressor Index (ESI) | Baseline parent stress (no stated direction) | No change | Stat n/r | | Non-sig* | | Garvey (2006) |
|  | Chicago Parent Program (CPP) | 292 | | Centre for Epidemiologic Studies Depression Scale (CESD) | Baseline parent depression (no stated direction) | No change | Stat n/r | | Non-sig* | | Garvey (2006) |
|  | Webster-Stratton’s  Incredible Years program | 106 | | Brief Symptom Inventory | Increased parent depression score | No change | *r* (44) = .05 | | *p* = .74* | | Baker (2011) |
|  | Barkley (1997)’s Behavioural Parent Training (BPT) program | 72 | | Disruptive Behaviour Stress Inventory | Parenting stress (no stated direction) | No change | Stat n/r | | Non-sig* | | Hellenthal (2009) |
|  | Program for mothers who have recently divorced (unnamed) | 321 | | Psychiatric Epidemiology Research Interview Demoralisation scale | Maternal distress (no stated direction) | No change | *b* = .00,  SE = .01,  Beta = .04 | | *p* > .05* | | Winslow (2009) |
|  | Strong African American Families (SAAF) | 172 | | Centre for Epidemiologic Studies Depression scale (CESD) | Maternal depression (no stated direction) | No change | Stat n/r | | Non-sig* | | Brody (2006) |
| **Months since divorce** | | | | | | | | | | | |
|  | Program for mothers who have recently divorced (unnamed) | 321 | | Parent-report | Increased number of months since divorce | Decreased | *b* = -.04,  SE = .02,  Beta = -.21 | | *p* < .05 | | Winslow (2009) |
| **Parent perceived benefit** | | | | | | | | | | | |
|  | Parenting our Children to Excellence (PACE) | 114 | | The Raising Young Children Scale | Perceived benefits | No change | Beta = 0.00,  SE = .06,  *t* (93) = -.02 | | Non-sig | | Nordstrom (2008) |
| **Measures of parenting behaviours** | | | | | | | | | | | |
|  | Chicago Parent Program (CPP) | 292 | | Toddler Care Questionnaire | Decreased self-efficacy | Increased | *r* = -.20 | | *p* < .05 | | Garvey (2006) |
|  | PACE (Parenting Our Children to Excellence) | 322 | | Parenting Efficacy subscale of the Parenting Sense of Competence Scale | Decreased self-efficacy | Decreased | Beta = -.10,  *t* (93) = -2.08 | | *p* = .040 | | Nordstrom (2008) |
|  | Strengthening Families Program: For Parents and Youth 10-14 (SFP) | 115 | | Warmth of Parenting Scale | Increased warmth | Increased | OR = .36,  CI = [.21, 0.64] | | *p* < .001 | | Skarstrand (2009) |
|  | Strengthening Families Program: For Parents and Youth 10-14 (SFP) | 115 | | Study-specific questions answered by parent (parents asked to choose one statement from four potential options) | More restrictive attitude to alcohol | No change | OR = 1.63,  CI = [.75, 3.57] | | *p* > .05 | | Skarstrand (2009) |
|  | Driving Mum and Dad Mad | 723 | | Parenting Scale (PS) | More positive parenting style | Increased participation, unless parenting conflict added to regression | χ^2^ (5, N = 154) = 16.69 | | *p* < .01 | | Calam (2008) |
|  | Program for mothers who have recently divorced (unnamed) | 321 | | Composite of self-report scales (CRPBI, Oregon Social Learning Centre) | Effective discipline | No change | *b* = -.26,  SE = .18,  Beta = -.17 | | *p* > .05 | | Winslow (2009) |
|  | Strengthening Families Program: For Parents and Youth 10-14 (SFP) | 115 | | Parent-report of rule-setting in the home | Rule-setting (no stated direction) | No change | OR = .67,  CI = [.38, 1.20] | | *p* > .05 | | Skarstrand (2009) |
|  | Parenting our Children to Excellence (PACE) | 114 | | Parent Efficacy subscale of the Parent Sense of Competence Scale | Positive attributions (no stated direction) | No change | *b* = -.03,  SE = .51,  *t* (93) = -.06 | | Non-sig | | Nordstrom (2008) |
|  | Parenting our Children to Excellence (PACE) | 114 | | Parent Efficacy subscale of the Parent Sense of Competence Scale | Negative attributions (no stated direction) | No change | Beta = .71,  SE = .47,  *t* (93) = 1.50 | | Non-sig | | Nordstrom (2008) |
|  | Driving Mum and Dad Mad | 723 | | Parent Problem Checklist (PPC) problem scale | More conflict | Decreased | χ^2^ (7, N = 154) = 20.84 | | *p* < .005 | | Calam (2008) |
|  | Strengthening Families Program: For Parents and Youth 10-14 (SFP) | 115 | | Study-specific questions answered by parent | Parent's knowledge of school performance (no stated direction) | No change | OR = 1.16,  CI = [.65, 2.09] | | *p* > .05 | | Skarstrand (2009) |
|  | Triple P | 257 | | Alabama Parenting Questionnaire | Parenting problems (no stated direction) | No change | OR = .40,  CI = [.14, 1.12] | | *p* > .05 | | Eisner (2011) |
| **Parent social support** | | | | | | | | | | | |
|  | Webster-Stratton’s Incredible Years program | 106 | | Social Support Appraisal Scale | Decreased parents perceived social support | No change | *r* (42) = -.06 | | *p* = .71 | | Baker (2011) |
|  | Triple P | 257 | | Parent-report | Strong neighbourhood social networks | Increased | OR = 4.32,  CI = [1.30, 14.30] | | *p* < .05 | | Eisner (2011) |
| **Parent cognitions** | | | | | | | | | | | |
|  | PACE (Parenting Our Children to Excellence) | 114 | | Study-specific questions answered by parent | Parental cognitions (no stated direction, this factor is a combination of several cognitions) | Increased | *F* (18,111) = 2.38 | | *p* = .004 | | Nordstrom (2008) |
| **Religious involvement** | | | | | | | | | | | |
|  | Strong African American Families (SAAF) | 172 | | Study-specific questions answered by parent | Low religious involvement | No change | Stat n/r | | Non-sig | | Brody (2006) |
| **3. Child factors** | | | | | | | | | | | |
| **Child age** | | | | | | | | | | | |
|  | Prevention Program for Externalizing Problem Behaviour (PEP) | 74 | | Study-specific questions answered by parent | Child age (no stated direction) | No change | Stat n/r | | *p* = .139* | | Plueck (2010) |
|  | Common Sense Parenting (CSP) | 157 | | Baseline parent interview | Child age (no stated direction) | No change | Beta = -4.18,  SE = 2.35 | | *p* > .05* | | Fleming (2015) |
|  | Parenting our Children to Excellence (PACE) | 114 | | Study-specific questions answered by parent | Child age (no stated direction) | No change | Beta = .50,  SE = .36,  *t* (93) = 1.41 | | Non-sig* | | Nordstrom (2008) |
| **Child gender** | | | | | | | | | | | |
|  | Common Sense Parenting (CSP) | | 157 | Baseline parent interview | Child gender (male) | Increased | Beta = 11.94,  SE = 4.45 | | *p* < .01* | Fleming (2015) | |
|  | Strengthening Families Program: For Parents and Youth 10-14 (SFP) | | 115 | Study-specific questions answered by parent | Child gender (male) | No change | OR = 1.63,  CI = [.93, 2.86] | | *p* > .05* | Skarstrand (2009) | |
|  | Prevention Program for Externalizing Problem Behaviour (PEP) | | 74 | Study-specific questions answered by parent | Child gender (male) | No change | Stat n/r | | *p* = .233* | Plueck (2010) | |
|  | Chicago Parent Program (CPP) | | 292 | Study-specific questions answered by parent | Child gender (male) | No change | Stat n/r | | Non-sig* | Garvey (2006) | |
|  | Parenting our Children to Excellence (PACE) | | 114 | Study-specific questions answered by parent | Child gender (no stated direction) | No change | Beta = .29,  SE = .61,  *t* (93) = .47 | | Non-sig* | Nordstrom (2008) | |
| **Child mental health symptoms** | | | | | | | | | | | |
|  | Chicago Parent Program (CPP) | 292 | | Eyberg Child Behaviour Inventory (EBCI) | Increased behaviour problems | Increased | *r* = .19 | | *p* < .05* | Garvey (2006) | |
|  | Chicago Parent Program (CPP) | 292 | | Caregiver-Teacher Report Form (CTRF) | Teacher-rated child behaviour problems | No change | Stat n/r | | Non-sig | Garvey (2006) | |
|  | Driving Mum and Dad Mad | 723 | | Eyberg Child Behaviour Inventory (ECBI) | More problematic child behaviour | Increase | χ^2^ (4, N = 154) = 12.09 | | *p* < .05* | Calam (2008) | |
|  | Bridges to High School | 292 | | Externalising Subscale of Child Behaviour Checklist (CBCL) answered by teacher | Increased externalising symptoms | More likely to attend but drop out early | Logit = .05, SE = .02,  OR = 1.05 | | *p* < .05* | Mauricio (2014) | |
|  | Webster-Stratton’s Incredible Years program | 106 | | Teacher Report Form | Increased child externalising behaviours | No change | *r* (49) = .22 | | *p* = .14 | Baker (2011) | |
|  | Triple P | 257 | | Social Behaviour Questionnaire (Externalising Problem Behaviour subscale) | Child externalising problem behaviour (no stated direction) | No change | OR = .65,  CI = [.22, 1.92] | | *p* > .05* | Eisner (2011) | |
|  | Common Sense Parenting (CSP) | 157 | | Strengths and Difficulties Questionnaire | Increasing child conduct problems | No change | Beta = .66,  SE = 2.25 | | *p* > .05* | Fleming (2015) | |
|  | Prevention Program for Externalizing Problem Behaviour (PEP) | 74 | | Parent-report (screening): Child Behaviour Checklist | Child externalising behaviours (no stated direction) | No change | Stat n/r | | *p* = .311 | Plueck (2010) | |
|  | Prevention Program for Externalizing Problem Behaviour (PEP) | 74 | | Teacher-report (screening): Child Behaviour Checklist | Child externalising behaviours (no stated direction) | No change | Stat n/r | | *p* = .453 | Plueck (2010) | |
|  | Prevention Program for Externalizing Problem Behaviour (PEP) | 74 | | Parent-report (pre-test): Child Behaviour Checklist | Child externalising behaviours (no stated direction) | No change | Stat n/r | | *p* = .530* | Plueck (2010) | |
|  | Prevention Program for Externalizing Problem Behaviour (PEP) | 74 | | Teacher-report (pre-test): Child Behaviour Checklist | Child externalising behaviours (no stated direction) | No change | Stat n/r | | *p* = .652 | Plueck (2010) | |
|  | Parenting our Children to Excellence (PACE) | 114 | | Disruptive Behaviour Disorders rating scale | ADHD symptoms (no stated direction) | No change | Beta = -.03,  SE = .04,  *t* (93) = -.73 | | Non-sig* | Nordstrom (2008) | |
|  | Parenting our Children to Excellence (PACE) | 114 | | Disruptive Behaviour Disorders rating scale | ODD symptoms (no stated direction) | No change | Beta = .04,  SE = .08,  *t* (93) = .54 | | Non-sig* | Nordstrom (2008) | |
|  | Bridges to High School | 353 | | Externalising score on Child Behaviour Checklist (CBCL) | Child externalising symptoms (no stated direction) | No change | *b* = -.09,  SE = .03,  Beta = -.05 | | *p* > .05* | Carpentier (2007) | |
|  | Bridges to High School | 353 | | Internalising score on Child Behaviour Checklist (CBCL) | Child internalising symptoms (no stated direction) | No change | *b* = .03,  SE = .02,  Beta = .10 | | *p* > .05* | Carpentier (2007) | |
|  | Prevention Program for Externalizing Problem Behaviour (PEP) | 74 | | Teacher-report (screening): Child Behaviour Checklist | Higher child internalising behaviours | Decrease | Stat n/r | | *p* = .025 | Plueck (2010) | |
|  | Common Sense Parenting (CSP) | 157 | | Strengths and Difficulties Questionnaire | Child emotional symptoms (no stated direction) | No change | Beta = -1.56,  SE = 2.47 | | *p* > .05* | Fleming (2015) | |
|  | Prevention Program for Externalizing Problem Behaviour (PEP) | 74 | | Parent-report (screening): Child Behaviour Checklist | Child internalising behaviours (no stated direction) | No change | Stat n/r | | *p* = .931 | Plueck (2010) | |
|  | Prevention Program for Externalizing Problem Behaviour (PEP) | 74 | | Parent-report (pre-test): Child Behaviour Checklist | Child internalising behaviours (no stated direction) | No change | Stat n/r | | *p* = .798* | Plueck (2010) | |
|  | Prevention Program for Externalizing Problem Behaviour (PEP) | 74 | | Teacher-report (pre-test): Child Behaviour Checklist | Child internalising behaviours (no stated direction) | No change | Stat n/r | | *p* = .424 | Plueck (2010) | |
|  | Barkley (1997)’s Behavioural Parent Training (BPT) program | 72 | | Ohio Scales | More severe child behaviour symptoms | Increased | *r* = .24 | | *p* = .05* | Hellenthal (2009) | |
|  | Program for mothers who have recently divorced (unnamed) | 321 | | Child Behaviour Checklist (CBCL) | Child maladjustment (no stated direction) | No change | *b* = .00,  SE = .01,  Beta = .03 | | *p* > .05* | Winslow (2009) | |
|  | Webster-Stratton’s Incredible Years program | 106 | | Teacher Report Form | Increased rule breaking behaviour | Increased | *r* (49) = .29 | | *p* = .05* | Baker (2011) | |
|  | Strengthening Families Program: For Parents and Youth 10-14 (SFP) | 115 | | Scale constructed for study by researchers | Increased norm-breaking behaviours | No change | OR = .88,  CI = [.55, 1.42] | | *p* > .05* | Skarstrand (2009) | |
| **Child's exposure to negative life events** | | | | | | | | | | | |
|  | Program for mothers who have recently divorced (unnamed) | 321 | | Negative Life Events Scale | Child's exposure to negative life events | No change | *b* = .00,  SE = .04,  Beta = .00 | | *p* > .05 | | Winslow (2009) |
| **Child’s academic success** | | | | | | | | | | | |
|  | Common Sense Parenting (CSP) | | 157 | Child-report | Child's school grades (no stated direction) | No change | Beta = -.82,  SE = 2.26 | | *p* > .05 | | Fleming (2015) |
|  | Bridges to High School | | 353 | Letter grades aggregated across quarters to yield a GPA | Child GPA (no stated direction) | No change | *b* = .18,  SE = .07,  Beta = .14 | | *p* < .05 | | Carpentier (2007) |
| **Youth unconventionality** | | | | | | | | | | | |
|  | Strong African American Families (SAAF) | 172 | | Assessed by standardising and summing youths’ responses to three measures; willingness to have sex, ability to resist peer pressure, resistance efficacy | Increased youth unconventionality | Decreased | Beta = -.43 (SEM model; χ^2^ (40, N = 164) = 33.36, *p* = .76) | | *p* <.05 | | Brody (2006) |
| **Perceived burden of child's behaviours** | | | | | | | | | | | |
|  | Prevention Program for Externalizing Problem Behaviour (PEP) | | 74 | Teacher-reported burden for themselves due to child's behaviours | Increased burden of child's behaviours | No change | OR = 3.04 | | *p* = .326 | | Plueck (2010) |
|  | Prevention Program for Externalizing Problem Behaviour (PEP) | | 74 | Parent-reported burden for themselves due to child's behaviours | Increased burden of child's behaviours | No change | Stat n/r | | *p* = .672 | | Plueck (2010) |
|  | Prevention Program for Externalizing Problem Behaviour (PEP) | | 74 | Teacher’s reported need for additional assistance in the class room due to child’s problems | Increased need for help | No change | Stat n/r | | *p* = .061 | | Plueck (2010) |
|  | Prevention Program for Externalizing Problem Behaviour (PEP) | | 74 | Parent’s reported need for additional assistance | Increased need for help | No change | Stat n/r | | *p* = 9.08 | | Plueck (2010) |
| **4. Parent/child relational factors** | | | | | | | | | | | |
| **Parent-child affect quality** | | | | | | | | | | | |
|  | Program for mothers who have recently divorced (unnamed) | 321 | | 2 subscales from Child Report of Parenting Behaviour Inventory and Parent-Adolescent Communication Scale | Mother-child relationship quality | No change | *b* = -.04,  SE = .15,  Beta = .03 | | *p* > .05 | | Winslow (2009) |
|  | Common Sense Parenting (CSP) | 157 | | Parent-report of parent-child affective quality (12 survey items pertaining to frequency of behaviours in prior month) | Parent-child affective quality | No change | Beta = -2.13,  SE = 2.36 | | *p* > .05 | | Fleming (2015) |
|  | Strong African American Families (SAAF) | 172 | | Interaction Behaviour Questionnaire (IBQ); mother- and adolescent-report | Poor relationship quality | No change | Stat n/r | | Non-sig | | Brody (2006) |
| **Family management** | | | | | | | | | | | |
|  | Common Sense Parenting (CSP) | 213 | | Alabama Parenting Questionnaire | Family management | No change | Beta = -.53,  SE = 2.13 | | *p* > .05 | | Fleming (2015) |
| **4. Barriers to engagement/service preferences** | | | | | | | | | | | |
| **Personal and family obstacles** | | | | | | | | | | | |
|  | PACE (Parenting Our Children to Excellence) | 114 | | The Obstacles to Engagement Scale (OES) | Increased personal and family obstacles | Decreased | Beta = -.29,  *t* (93) = -2.10 | | *p* = .038 | | Nordstrom (2008) |
| **Time and scheduling difficulties** | | | | | | | | | | | |
|  | PACE (Parenting Our Children to Excellence) | 114 | | The Obstacles to Engagement Scale (OES) | Fewer time and scheduling barriers | Increased | Beta = .74,  *t* (93) = 3.99 | | *p* < .001 | | Nordstrom (2008) |
| **Service preferences** | | | | | | | | | | | |
|  | Common Sense Parenting (CSP) | 213 | | Common Sense Parenting versus Common Sense Parenting Plus | Child attending program | No change | Beta = -4.02,  SE = 4.54 | | *p* > .05 | | Fleming (2015) |
|  | Bridges to High School | 292 | | Moos Group Environment scale | Increased cohesion | Increased | Logit = .25, SE = .12,  OR = 1.28 | | *p* < .05 | | Mauricio (2014) |
|  | Bridges to High School | 292 | | Familism subscale of the Acculturation Rating Scale for Mexican-Americans-II | Increased Perceived familism | Increased | Logit = 1.03, SE = .54,  OR = 2.80 | | *p* < .05 | | Mauricio (2014) |
|  | Chicago Parent Program (CPP) | 292 | | Geographical location | Living within 3 miles of day care centre | Decreased | *t* (153) = -2.3 | | *p* < .05 | | Garvey (2006) |
|  | Parenting our Children to Excellence (PACE) | 114 | | The Obstacles to Engagement Scale (OES) | Increased program relevance/trust | No change | Beta = .02,  SE = .14,  *t* (93) = .17 | | Non-sig | | Nordstrom (2008) |
|  | Triple P | 257 | | Study-specific questions answered by parent | Previous parent service utilisation | No change | OR = .79,  CI = [.23, 2.65] | | *p* > .05 | | Eisner (2011) |
|  | Parenting our Children to Excellence (PACE) | 114 | | The Obstacles to Engagement Scale (OES) | Low intervention demands | No change | Beta = -.04,  SE = .18,  *t* (93) = -.23 | | Non-sig | | Nordstrom (2008) |
| **6. Engagement factors** | | | | | | | | | | | |
| **Intent to enrol** | | | | | | | | | | | |
|  | Bridges to High School | 292 | | Provider ratings of parent’s intentions | Less intention to attend | Decreased | Logit = 0.39, SE = .15,  OR = 1.47 | | *p* < .05 | | Mauricio (2014) |
| **Attendance at first sessions** | | | | | | | | | | | |
|  | Chicago Parent Program (CPP) | 292 | | Attendance records | Attendance at first session | Increased | 91% more likely to attend at least 2 sessions | | n/a | | Garvey (2006) |
| **Engagement in sessions** | | | | | | | | | | | |
|  | Chicago Parent Program (CPP) | 292 | | Attendance records | Increased engagement in sessions | Increased | *r* = .59 | | *p* < .001 | | Garvey (2006) |

Notes:

*Indicates *p*-values selected for Stouffer’s *p* analysis
