# Supplementary material for: Parental engagement in preventive parenting programs for child mental health: a systematic review of predictors and strategies to increase engagement
Source: PeerJ. 2018 Apr 27;6:e4676. doi: 10.7717/peerj.4676 (PMC5926551; doi:10.7717/peerj.4676)
Supplement: Supplemental Information 6 [file peerj-06-4676-s006.docx]

### Online Supplement 5: Descriptive Summary of Risk of Bias of Included Quantitative Studies

Cochrane Collaboration’s tool used to assess risk of bias in quantitative studies (handbook.cochrane.org/chapter_8/table_8_5_a_the_cochrane_collaborations_tools_for_assessing.html)

Table 5

*Descriptive Summary of Risk of Bias of Included Quantitative Studies*

| Study | Selection bias | | Performance bias | Detection bias | Attrition bias | Reporting bias | Other bias |
| --- | --- | --- | --- | --- | --- | --- | --- |
|  | Random sequence generation | Allocation concealment | Blinding of participants and personnel | Blinding of outcome assessment | Incomplete outcome data | Selective reporting | Other sources of bias |
| Aalborg (2012) and Miller (2011) | Risk unclear: specific method n/r, beyond ‘randomly’ assigned to choice or control condition. p. 3. Miller (2011) | Risk unclear: supporting data n/r | High risk: no blinding attempted | Risk unclear | Risk unclear | Risk unclear: insufficient information to permit judgement of low or high risk; no protocol available | Risk unclear |
| Baker (2011) | Risk unclear: specific method n/r, beyond ‘half of the classrooms were randomly  assigned to a parent training intervention group’. p. 129 | Risk unclear: insufficient information to permit judgement of low or high risk | Risk unclear: supporting data n/r | Risk unclear: unclear whether teachers were blinded to group allocation | Risk unclear | Risk unclear: insufficient information to permit judgement of low or high risk | Risk unclear |
| Bjorknes (2011) and Bjorknes (2013) | Low risk: ‘randomization sequences were computer generated  (Microsoft Excel)’. p. 55. Bjorknes (2013) | Low risk: ‘randomization procedures were carried out after the entire group of study participants had completed the baseline interview.’ p. 55. Bjorknes (2013) | Risk unclear: supporting data n/r | Unclear risk: unclear whether teachers were blinded to group allocation | Low risk: missing data imputed using acceptable methods. ‘Missing-completely-at-random  (MCAR) test was carried out for each instrument, and the  statistical method expectation–maximization (EM) was used  to estimate and fill in missing values at the item level.’ p. 57. Bjorknes (2013) | Risk unclear: insufficient information to permit judgement of low or high risk | Risk unclear |
| Brody (2006) | Risk unclear: randomisation method n/r | Risk unclear: insufficient information to permit judgement of low or high risk | Risk unclear: supporting data n/r | Risk unclear | Risk unclear | Risk unclear: insufficient information to permit judgement of low or high risk | Risk unclear |
| Byrnes (2012) | Risk unclear: randomisation method n/r beyond ‘families were  randomly assigned to one of two programs or a control condition’. p. 178 | Risk unclear: supporting data n/r | High risk: no blinding attempted | Risk unclear | Risk unclear: supporting data n/r | Risk unclear: insufficient information to permit judgement of low or high risk | Risk unclear |
| Calam (2008) | Risk unclear: randomisation method n/r. Only reported that  ‘randomization produced  similar, comparable groups’. p. 329 | Low risk: central allocation (web-based randomisation procedure) | Low risk: outcome not likely to be influenced by lack of blinding due to use of web-based platform for study | Low risk | Risk unclear: supporting data n/r | Risk unclear: insufficient information to permit judgement of low or high risk | Risk unclear |
| Carpentier (2007) | Risk unclear: randomisation method n/r. Only reported that  ‘families were randomly assigned to either the control or intervention condition’. p. 528 | Risk unclear: insufficient information to permit judgement of low or high risk | Risk unclear: supporting data n/r | Risk unclear | High risk: ‘listwise deletion excluded participants with missing data from the analyses regarding program enrolment’. p. 533 | Risk unclear: insufficient information to permit judgement of low or high risk; no protocol available | High risk: unequal probability assignment. English families were given a 70% chance of being placed in treatment and 30% chance of being placed in control. Conversely, Spanish families were given a 60% chance of being placed in treatment, and a 40% chance of placement in control |
| Eisner (2011) | Risk unclear | Risk unclear: insufficient information provided | Risk unclear: supporting data n/r | Risk unclear | Risk unclear: supporting data n/r | Risk unclear: insufficient information to permit judgement of low or high risk; no protocol available | Risk unclear |
| Fleming (2015) | Low risk:  ‘…assigned identification  numbers in the order in which participants returned  permission slips and then blocked the participants by  school and adolescent gender. Within blocks…  assigned families sequentially to one of the three  experimental conditions…or a  no-intervention control condition’. p. 108 | Risk unclear: supporting data n/r | Risk unclear: ‘staff person who made assignments to condition had no contact with individual families and had no information on families other than identification numbers, gender of the students, and the students’ schools’. p. 108  However, unclear whether participants were blinded to allocation | Low risk: ‘data collection staff, who were not informed of condition assignments…’.  p. 108 | Risk unclear: supporting data n/r | Risk unclear: insufficient information to permit judgement of low or high risk; no protocol available | Risk unclear |
| Garvey (2006) | Risk unclear: randomisation procedure not clear. ‘7-day care centers were assigned  to one of two equivalent groups that were  matched on size, racial/ethnic composition, median  income, and percent single-family households  and then randomly assigned to the intervention or  waiting list control condition’. p. 205 | Risk unclear | Risk unclear: supporting data n/r | Risk unclear: PT group leaders reported on parents’ engagement in program, but unclear whether leaders themselves were blinded to group allocation | Risk unclear: supporting data n/r | Risk unclear: insufficient information to permit judgement of low or high risk | Risk unclear |
| Heinrichs (2005) | High risk:  ‘families were assigned to the experimental  or control group based on preschool affiliation.  Preschools were randomized to the two conditions  after being matched according to the social structure  of their respective neighbourhoods’. p. 278 | High risk: inadequate concealment of interventions prior to allocation. Participants were aware of possibility they would be allocated to one of two conditions (control or experimental) | High risk | Risk unclear: unclear whether teachers rating participating families were themselves blinded to group allocation | Risk unclear: supporting data n/r | Risk unclear: insufficient information to permit judgement of low or high risk | Risk unclear |
| Heinrichs (2006) | Unclear risk: specific randomisation procedure n/r -‘preschools were first matched based on their size  and then randomly assigned to one of the four recruitment conditions’. p. 349 | Risk unclear: method of concealment is not described in sufficient detail to allow a definitive judgement | High risk:  ‘one of these female recruiters was aware of the  main hypotheses of the study while the other one was blind. Complete blindness was not possible because half of the female recruiters were also responsible for  conducting the prevention program’. p. 349-50 | Risk unclear | Risk unclear | Risk unclear: insufficient information to permit judgement of low or high risk | Risk unclear |
| Helfenbaum-Kun (2007) | Risk unclear: randomisation method n/r, merely that ‘fathers were randomly assigned to a parent-training group consistent with their language preference, or to a  no-treatment control group’. p. 53 | Risk unclear | Risk unclear | Risk unclear:  ‘Head Start teachers completed the Intensity scale of the Sutter-Eyberg  Student Behavior Inventory-Revised’. p. 53  However unclear whether teachers were blinded to group allocation | Risk unclear | Risk unclear: insufficient information to permit judgement of low or high risk | Risk unclear |
| Hellenthal (2009) | Low risk: intervention group only, no control group | Low risk: intervention group only, no control group | Low risk: intervention group only, no control group | Low risk: intervention group only, no control group. Furthermore, data collected in an anonymous fashion as parents utilised a code on all forms except the consent form, which was kept separate from all other forms | Risk unclear: supporting data n/r | Risk unclear: insufficient information to permit judgement of low or high risk | Risk unclear |
| Mauricio (2014) | Risk unclear: randomisation method n/r | Risk unclear | Risk unclear | Risk unclear: ‘teachers completed  paper–pencil questionnaires on child behavior’. p. 373. Unclear whether teachers were blinded to condition allocation | Risk unclear: supporting data n/r | Risk unclear: insufficient information to permit judgement of low or high risk | Risk unclear |
| Mian (2015) | Risk unclear: ‘parents were randomized to two recruitment  strategies’. p. 61. However, specific randomisation method n/r | Risk unclear | Risk unclear | Risk unclear | Low risk: missing data for variables associated with hypotheses were imputed using multiple imputation in PASW Statistics 18 with 20 imputations, informed by sociodemographic and psychological variables | Risk unclear: insufficient information to permit judgement of low or high risk | Risk unclear |
| Nordstrom (2008) | Risk unclear | Risk unclear | Risk unclear | Risk unclear | High risk: nine mothers with missing data were excluded from analyses. Imputation not implemented | Risk unclear: insufficient information to permit judgement of low or high risk | Risk unclear |
| Plueck (2010) | Risk unclear: randomisation method n/r | Risk unclear | Risk unclear | Risk unclear regarding teacher-reported data, and whether they were blinded to group allocation | Risk unclear: supporting data n/r | Risk unclear: insufficient information to permit judgement of low or high risk | Risk unclear |
| Reedtz (2011) | Risk unclear: ‘children and families were randomized to either the shortened basic version (*n*=89), or the control group (*n*=97)’. p. 268. However, randomisation method n/r | Risk unclear | Risk unclear | Risk unclear | Risk unclear | Risk unclear: insufficient information to permit judgement of low or high risk | Risk unclear |
| Skarstrand (2009) | Risk unclear: ‘schools were stratified on socio-economic position: 12 in high-income areas and ten in  low-income areas. Half of the schools in the high-income areas, and half of the schools in  the low-income areas were randomly assigned to form the intervention group or the control group’. p. 386. Specific randomisation method n/r | Risk unclear | Risk unclear: supporting data n/r | Risk unclear | Risk unclear: supporting data n/r | Risk unclear: insufficient information to permit judgement of low or high risk | Risk unclear |
| Winslow (2009) | Risk unclear: specific randomisation method n/r, beyond ‘mothers were told  that they would be randomly assigned to one of three program conditions’. p. 157 | Risk unclear | Risk unclear | Risk unclear | Risk unclear | Risk unclear: insufficient information to permit judgement of low or high risk | Risk unclear |

Notes:

n/r= not reported
