# Supplementary material for: Parental engagement in preventive parenting programs for child mental health: a systematic review of predictors and strategies to increase engagement
Source: PeerJ. 2018 Apr 27;6:e4676. doi: 10.7717/peerj.4676 (PMC5926551; doi:10.7717/peerj.4676)
Supplement: Supplemental Information 7 [file peerj-06-4676-s007.docx]

**The rationale for conducting the meta-analysis**

A meta-analysis was not possible due to substantial differences in interventions, settings, predictor variables, and analytic methods. To partly compensate for this limitation, the Stouffer’s method (Stouffer et al., 1949) of combining *p*-values was identified as an appropriate method for synthesizing the findings of many of the included studies. The rationale for conducting a Stouffer’s P test is to allow for the synthesizing of available information on the predictors of parental engagement across the 3 stages of engagement (intent, enrolment and attendance). It is hoped through the review and synthesis of this literature we can identify both predictors of parental engagement and strategies that could increase parent engagement in preventive parenting programs for child mental health

**The contribution that the meta-analysis makes to knowledge in light of previously published related reports, including other meta-analyses and systematic reviews**

This assumption is developed from the theoretical underpinnings suggested by Sandler and colleagues (2011), that a parenting program improves parenting skills and parental self-efficacy, and causes a reduction in barriers to effective parenting, which in turn allows for long-term benefits for the child. Despite the potential benefits of preventive parenting programs, many studies examining the effectiveness of such programs have reported difficulties in engaging parents. Two previous reviews attempted to collate the extant research on parental engagement. Ingoldsby (2010) reviewed ongoing engagement and retention of families attending both intervention and indicated prevention programs designed to improve child mental health (child age range not specified). Notably this review did not include “…studies that focused on family enrolment unless the investigators also clearly hypothesised that the intervention would improve ongoing engagement or retention” (p.631). While Chacko et al. (2016) included a large number of studies because it examined multiple primary outcomes (including SES and child age), but did not focus on the factors influencing *initial engagement*. Another limitation of this review is that it did not review other types of evidence-based programs that have also reported engagement challenges The current systematic literature review adds to the literature and previously published reports through an examination of both the predictors of engagement and the strategies that researchers have used to increase engagement. Such a synthesis can inform researchers regarding the predictors of *initial* and *ongoing* parental engagement, and suggest some possible theoretical models that could be used in the development of engagement strategies to increase the uptake of evidence-based preventive parenting programs.
